# Different congenital hydrocephalus–associated mutations in Trim71 impair stem cell differentiation via distinct gain-of-function mechanisms

**DOI:** 10.1371/journal.pbio.3001947

**Published:** 2023-02-09

**Authors:** Qiuying Liu, Mariah K. Novak, Rachel M. Pepin, Katharine R. Maschhoff, Wenqian Hu

**Affiliations:** Department of Biochemistry and Molecular Biology, Mayo Clinic, Rochester, Minnesota, United States of America; Max F Perutz Laboratories Center of Molecular Biology, AUSTRIA

## Abstract

Congenital hydrocephalus (CH) is a common neurological disorder affecting many newborns. Imbalanced neurogenesis is a major cause of CH. Multiple CH-associated mutations are within the RNA-binding domain of Trim71, a conserved, stem cell–specific RNA-binding protein. How these mutations alter stem cell fate is unclear. Here, we show that the CH-associated mutations R595H and R783H in Trim71 accelerate differentiation and enhance neural lineage commitment in mouse embryonic stem cells (mESCs), and reduce binding to mRNAs targeted by wild-type Trim71, consistent with previous reports. Unexpectedly, however, each mutant binds an ectopic and distinct repertoire of target mRNAs. R595H-Trim71, but not R783H-Trim71 nor wild-type Trim71, binds the mRNA encoding β-catenin and represses its translation. Increasing β-catenin by overexpression or treatment with a Wnt agonist specifically restores differentiation of R595H-Trim71 mESCs. These results suggest that Trim71 mutations give rise to unique gain-of-function pathological mechanisms in CH. Further, our studies suggest that disruption of the Wnt/β-catenin signaling pathway can be used to stratify disease etiology and develop precision medicine approaches for CH.

## Abstract 2

This study shows that two congenital hydrocephalus (CH)-associated mutations in Trim71 accelerate mouse embryonic stem cells differentiation into neurons; while these mutations reduce binding to known Trim71 target mRNAs, the mutant Trim71s bind new and distinct targets, leading to gain-of-function effects that may contribute to the etiology of CH.

## Introduction

Congenital hydrocephalus (CH) is a common birth defect affecting approximately 1 in every 1,000 newborns [1]. It clinically manifests as the pathological expansion of the cerebral ventricles (ventriculomegaly) and abnormal accumulation of cerebrospinal fluid. Although CH is routinely treated by lifelong neurosurgical shunting, this procedure has numerous complications [2] and does not always restore neurodevelopment, making CH a significant cause of childhood morbidity. The molecular basis of CH pathogenesis is largely unknown. This knowledge is essential to develop new preventive, diagnostic, and therapeutic approaches for CH.

Human exome sequencing studies identified multiple CH-associated mutations in several genes that are specifically expressed in embryonic and neural stem cells [3,4]. These observations argue that impaired/imbalanced neural differentiation is the primary cause of CH and that the abnormal accumulation of cerebrospinal fluid in the brain is a consequence [5].

A number of CH-associated missense mutations have been identified in the gene *Trim71* [3,4]. Trim71 is an evolutionarily conserved RNA-binding protein (RBP) that is abundantly expressed in embryonic and neural stem cells [6,7]. Genetic studies in multiple model organisms revealed that Trim71 is essential for early embryogenesis and proper neural differentiation [6]. Mechanistically, Trim71 binds target mRNAs and triggers translational repression and/or mRNA degradation [8–13]. Trim71-mediated posttranscriptional regulation is critical for proper stem cell differentiation [8,13].

The pathogenesis resulting from the CH-associated mutations in Trim71 is also conserved between mouse and human, as mice with CH-specific Trim71 mutations display ventriculomegaly, cerebrocortical hypoplasia, progressive macrocephaly, and impaired neurogenesis [14]. However, the molecular mechanisms by which CH-associated mutations in Trim71 lead to defects in stem cell differentiation and neurogenesis are still incompletely understood.

All the CH-associated mutations in Trim71 are within its RNA-binding domain (the NHL domain) (S1A Fig) [4], suggesting that these mutations alter the RNA-binding capability of Trim71. We recently showed that one such CH-associated mutation, R783H, significantly changes the repertoire of mRNAs bound by Trim71. R783H-Trim71, but not wild-type (WT) Trim71, binds to *Lsd1* mRNA and represses its translation, resulting in stem cell differentiation defects [15]. The CH-associated R595H-Trim71 has overall weaker binding to mRNA than WT-Trim71 [14]. However, the mRNA substrates of R595H-Trim71 and the pathogenic mechanisms of distinct Trim71 mutants are still unclear.

Here, we found that although R783H-Trim71 and R595H-Trim71 lead to premature differentiation and accelerated neural lineage commitment in mouse embryonic stem cells (mESCs), each Trim71 mutant binds a distinct and ectopic subset of target mRNAs. We discovered that R595H-Trim71, but not R783H-Trim71 nor WT-Trim71, specifically binds and represses the translation of *Ctnnb1* mRNA, which encodes β-catenin. This repression leads to decreased Wnt/β-catenin signaling activity specifically in R595H-Trim71 mutant mESCs. Increasing β-catenin levels alleviates the differentiation defects in the R595H-Trim71 mESCs, but not in R783H mESCs. These data suggest that gain-of-function mutations in the RNA-binding domain of Trim71 cause CH via distinct pathological mechanisms, that CH patients could be stratified based on these distinct mechanisms, and that precise therapeutic strategies could be designed for genetically diverse etiologies of CH.

## Results

### Two CH-associated mutations in Trim71 result in similar stem cell differentiation defects in mESCs

We used mESCs as a model for mechanistic studies of the CH-associated mutations in Trim71 because the pathogenesis caused by Trim71 mutants is conserved between mouse and human [14], and Trim71 is an evolutionarily conserved, stem cell–specific RBP [6,7]. Briefly, we employed FLAG-Trim71 mESCs with biallelic FLAG-tag knock-in at the N-terminus of the endogenous Trim71 loci [8] and used CRISPR/Cas9-mediated genome editing to introduce R595H or R783H in Trim71. We obtained four mutant mESC lines, representing monoallelic (R595H/+, R783H/+) and biallelic (R595H, R783H) mutations (S1B–S1E Fig).

**Fig 1:**
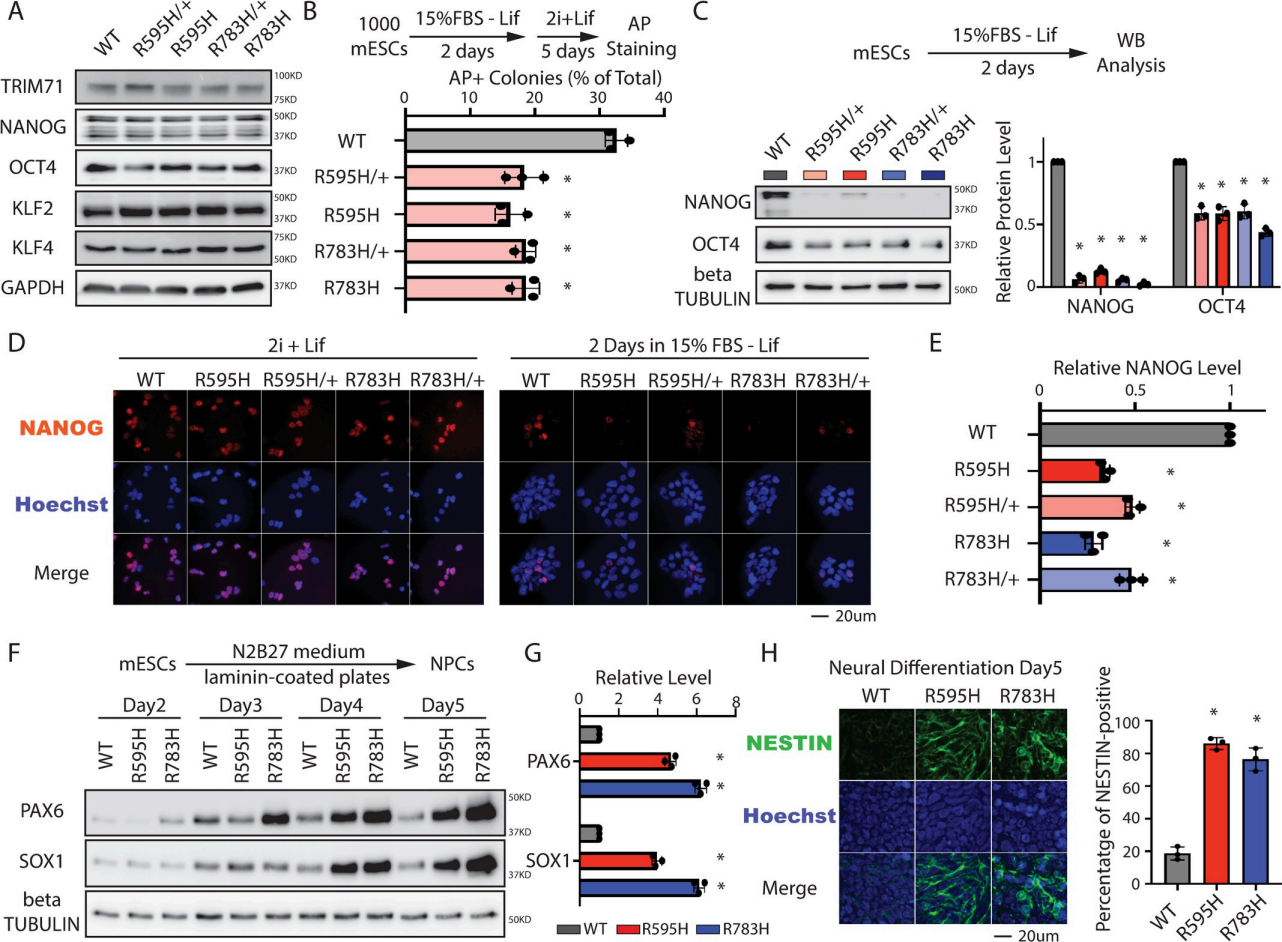
The two CH-associated mutations in Trim71 cause similar differentiation defects in mESCs. (**A**) Western blotting in the WT mESCs and mESCs with the CH-associated mutations in Trim71. The cells were cultured in the 2i + Lif medium. (**B**) Exit pluripotency assay for mESCs. The mESCs were induced to exit pluripotency in medium without Lif for 2 days and then switched to 2i+Lif medium for 5 days. The resultant colonies were fixed and stained for AP. The colony morphology and AP intensity were evaluated through microscopy. To determine the percentage of undifferentiated colonies, 100–200 colonies were examined each time. The results represent the means (± SD) of three independent experiments. (**C**) Western blotting of pluripotency factors during the spontaneous differentiation of mESCs. (**D**) IF staining of NANOG in mESCs cultured in the stemness (2i + Lif) and differentiating (15% FBS–Lif) conditions. (**E**) Relative intensity of IF NANOG signals from individual cells in the differentiating condition. (**F**) Expression of neural lineage markers during the directed neural differentiation. (**G**) Quantification of Pax6 and Sox1 protein level at the neural differentiation Day5. (**H**) IF staining of Nestin. The quantifications represent the means (± SD) of three independent experiments. In C, F, and G, representative western blots are shown, and the quantifications represent the means (± SD) of three independent experiments. *p < 0.05; and n.s. not significant (*p* > 0.05) by one-way ANOVA. See also S1–S4 Figs. The data for B, C, E, G, and H are available in S1 Data. CH, congenital hydrocephalus; IF, immunofluorescence; mESC, mouse embryonic stem cell; WT, wild-type.

The protein levels of pluripotency factors (Fig 1A) and colony formation (S2 Fig) were similar in Trim71 mutant and WT mESCs, suggesting that self-renewal was not impacted by the mutations. However, all four mutant lines displayed differentiation defects. First, when assayed for pluripotency exit [16], all the Trim71 mutant mESCs lost pluripotency faster than WT mESCs (Fig 1B). Second, during spontaneous differentiation, all the Trim71 mutant mESCs displayed lower levels of pluripotency factors, such as Nanog (Fig 1C–1E), when compared to WT mESCs. However, in contrast with Trim71 knockout (KO) mESCs [13], the mutant mESCs had similar rates of proliferation and apoptosis as WT mESCs, under both self-renewal and differentiating conditions (S3 Fig). Thus, the spontaneous differentiation defects in the Trim71 mutant mESCs are not caused by alterations in either proliferation or apoptosis. Given that the heterozygous and homozygous mutants displayed similar cellular and molecular phenotypes (Fig 1A–1E), we used the homozygous mutant mESCs for mechanistic studies.

CH is a neurological disorder that can be caused by impaired neurogenesis [5,14]. We found that neural lineage commitment was enhanced in both the R595H-Trim71 and R783H-Trim71 mESCs compared to WT mESCs, consistent with recent work [14]. Briefly, Sox1 and Pax6, two lineage-specific transcription factors essential for neuroectodermal specification, showed higher expression during directed neural differentiation of the two Trim71 mutant mESCs compared to WT mESCs (Fig 1F and 1G) [17]. Moreover, the neural stem/progenitor cell marker Nestin was also more highly expressed during neural differentiation of the two Trim71 mutant mESCs compared to WT mESCs (Fig 1H). Finally, embryoid bodies (EBs) generated by spontaneous differentiation of Trim71 mutant mESCs expressed higher levels of ectoderm markers, but not of endoderm or mesoderm makers, than EBs generated from either WT or Trim71 KO mESCs (S4 Fig). These results suggest that mESCs with CH-associated mutations in Trim71 are more prone to neural differentiation.

Altogether, these observations indicate that the two different CH-associated mutations in Trim71 lead to similar defects in embryonic stem cell differentiation and neural lineage commitment. However, our data also argue that these CH-associated mutations are gain-of-function mutations, leading to differentiation phenotypes that are not necessarily recapitulated by Trim71 KO.

### The two CH-associated Trim71 mutants bind distinct substrate mRNAs

**Fig 2:**
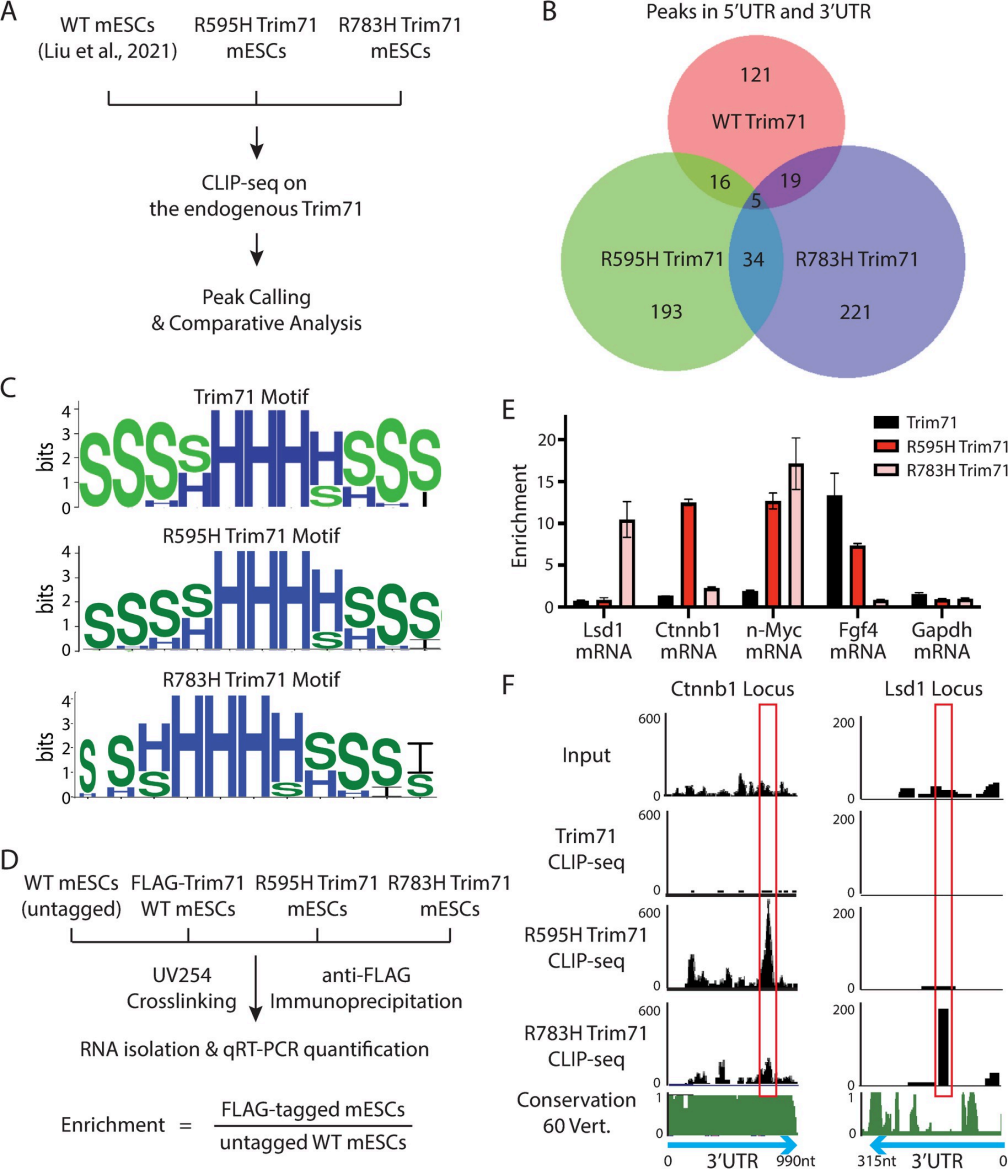
Comparative analysis of the transcriptome-wide target mRNAs of the WT Trim71 and the two Trim71 mutants in mESCs. (**A**) An outline of the comparative analysis on the CLIP-seq datasets. (**B**) Venn diagram showing mRNAs with binding sites from the WT Trim71 and the two Trim71 mutants. (**C**) Comparison of RNA secondary structures overrepresented in the WT Trim71 and the two Trim71 mutants. “H,” “S,” and “I” indicate a nucleotide in a hairpin loop region, a stack region, and an internal loop region, respectively. (**D**) A workflow of the CLIP-qRT-PCR to validate the target mRNAs identified from the CLIP-seq experiments. (**E**) CLIP-qRT-PCR for the identified target mRNAs of the two Trim71 mutants. The results represent the means (± SD) of three independent experiments from the cells cultured in the 2i + Lif medium. (**F**) UCSC genome browser snapshot for the CLIP-seq datasets from the WT Trim71 and the two Trim71 mutants in the 3′ UTRs of the *Ctnnb1* and *Lsd1*. The red boxes indicate the binding regions of the Trim71 mutants. The inputs are from the size-matched input samples in the CLIP-seq analysis. The conservation score determined from the genome sequences of 60 vertebrates is from the default track of the UCSC genome browser. See also S5 Fig and S1 Table. The data for E are available in S2 Data. CLIP-seq, crosslinking immunoprecipitation and sequencing; mESC, mouse embryonic stem cell; qRT-PCR, quantitative real-time PCR; WT, wild-type.

Both of the CH-associated mutations are located in the RNA-binding domain of Trim71. We recently showed that R783H-Trim71 and WT-Trim71 bind distinct subsets of mRNAs [15]. To determine whether R595H-Trim71 binds a different pool of mRNA substrates, we identified transcriptome-wide targets by crosslinking immunoprecipitation and sequencing (CLIP-seq) and performed comparative analysis with the CLIP-seq data from the R783H-Trim71 and the WT-Trim71 (Fig 2A) [8,15]. These analyses revealed three similarities. First, the WT-Trim71 and both Trim71 mutants mainly bound 3′ UTRs of their target mRNAs (S1 Table). Second, the binding sites from WT-Trim71 and the two Trim71 mutants had a similar overrepresentation of predicted stem-loop structures, but no enriched primary sequence motifs, compared to randomized sequences (Fig 2C). These data are consistent with the findings from in vitro studies that Trim71 recognizes structural motifs [18]. Third, similar to the R783H mutation, the R595H mutation also altered the mRNA substrates of Trim71 (Fig 2B).

Intriguingly, however, the two Trim71 mutants bound distinct sets of mRNAs with a very small overlap (Fig 2B). We validated this by CLIP-qRT-PCR (Fig 2D and 2E). For example, *Lsd1* mRNA, a target of the R783H Trim71 [15], was associated with the R783H-Trim71, but not with WT- or the R595H-Trim71; similarly, *Ctnnb1* mRNA was specifically bound by R595H-Trim71, but not by the WT- or R783H-Trim71 (Fig 2E). These results argue that different Trim71 mutants bind different sets of target mRNAs.

The difference in mRNA targets among the WT and the two Trim71 mutants was not due to alterations in cellular developmental state, as all the cells were maintained in the ground state using the 2i + Lif medium [19]. Moreover, mRNA availability does not appear to contribute to the differential RNA binding, because most (464 out of 466) RNA targets of R595H-Trim71 were not differentially expressed in the WT and the mutant mESCs (S5 Fig), similar to the RNA targets of R783H-Trim71 [15]. These observations suggest that different CH-associated mutations in Trim71 have distinct impacts on RNA target recognition by Trim71.

### Identification of potential functional target mRNAs of R595H-Trim71

**Fig 3:**
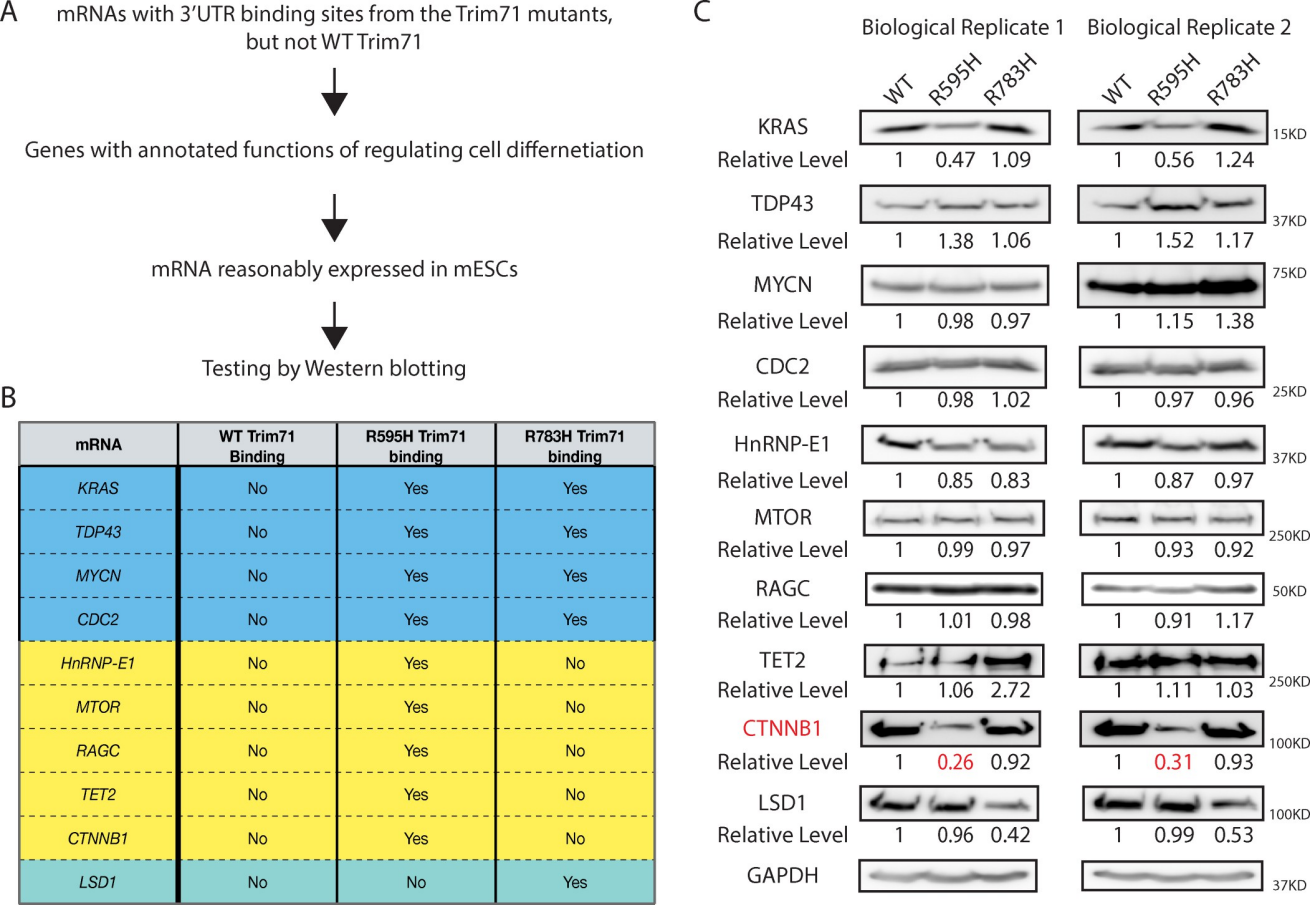
Identification of candidate genes for functional characterization. (**A**) Outline of strategies to identify potential functional targets of the Trim71 mutants. (**B**) A table summarizing the selected candidate mRNAs with binding sites from the Trim71 mutants. (**C**) Western blots in the WT, R595H Trim71, and R783H Trim71 mESCs cultured in 2i + Lif medium. GAPDH was used for normalization in determining the relative protein levels. mESC, mouse embryonic stem cell; WT, wild-type.

To identify the functional mRNA targets of the Trim71 mutants, we initially focused on the mRNAs that were bound by both the Trim71 mutants, but not by WT-Trim71, given that the two mutant mESCs displayed similar differentiation defects that were not evident with Trim71-KO mESCs. Among these 34 common targets, we focused on the ones with reasonable expression levels in mESCs and encoding genes with annotated functions in stem cell biology and neural differentiation (Fig 3A and 3B and S2 Table). However, among the four such mRNA tested, none of them displayed consistent alterations at the protein levels in the WT and two Trim71 mutant mESCs (Fig 3C). These results argue that ectopic binding of the two Trim71 mutants on these mRNAs does not affect transcript stability or translation and suggest that none of these common target mRNAs are the functional targets of the Trim71 mutant.

Given that the CH-associated mutations appear to be distinct gain-of-functions (Fig 2B), we considered the possibility that the similar defects in the two mutant mESCs might arise from binding and dysregulating different target mRNAs. We recently determined that *Lsd1* mRNA is a functional target of R783H-Trim71 [15]. Here, we observed that LSD1 protein was specifically decreased in the R783H mESCs, but not altered in the R595H mESCs (Fig 3C). This result further supported that the notation that the molecular mechanisms of the two Trim71 mutants may be different. Thus, to identify the functional target mRNAs of the R595H-Trim71, we applied the similar rationales (Fig 3A and S2 Table) on the target mRNAs that are specifically bound by the R595H-Trim71. Among the five such candidates we analyzed, only CTNNB1 displayed consistent and specific decrease at the protein level in the R595H mESCs (Fig 3B and 3C), suggesting that *Ctnnb1* mRNA is functional target of the R595H-Trim71. *Ctnnb1* mRNA encodes β-catenin, a key component of the classical Wnt/β-catenin signaling pathway that is essential for proper stem cell differentiation [20]. Interestingly, there is a major R595H-Trim71-specific binding site in the 3′ UTR of *Ctnnb1* mRNA (Fig 2F). Moreover, unlike many mRNAs, the *Ctnnb1* mRNA 3′ UTR is highly conserved among vertebrates (Fig 2F).

### R595H-Trim71 binds *Ctnnb1* mRNA and represses its translation

**Fig 4:**
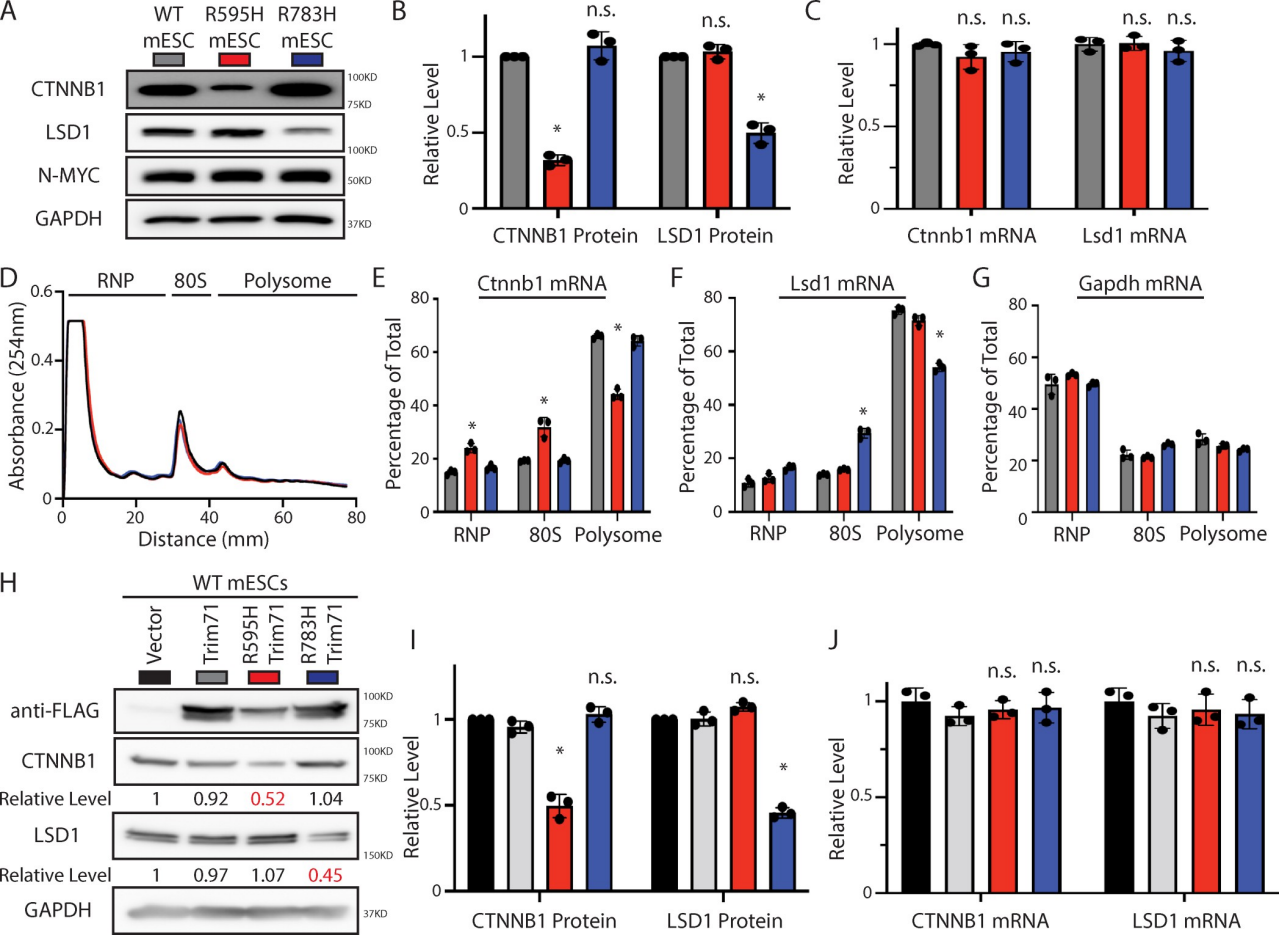
The two Trim71 mutants represses the translation of different mRNAs in mESCs. (**A**) Western blotting in the WT, R595H, and R783H mESCs. The cells were cultured in the 2i + Lif medium. The color code displayed in the panel are used in all the other panels of the figure. (**B**, **C**) Quantification of the protein (B) and mRNA (C) levels of Ctnnb1 and Lsd1 in the cells cultured in the 2i + Lif medium. GAPDH and 18S rRNA were used for normalization in protein and mRNA quantifications, respectively. (**D**) Polysome analysis in the WT and the two mutant mESCs cultured in the 2i + Lif medium. (**E**, **G**) Quantification of the indicated mRNA distribution in the RNP, 80S, and polysome fractions from the WT and the two mutant mESCs. (**H**) Western blotting in the WT mESCs expressing an empty vector, Flag-Trim71, Flag-Trim71(R595H), and Flag-Trim71(R783H). The cells were cultured in the 2i + Lif medium. (**I**, **J**) Quantification of the protein (I) and mRNA (J) levels of Ctnnb1 and Lsd1. GAPDH and 18S rRNA were used for normalization in protein and mRNA quantifications, respectively. The quantification results in B, C, E, F, G, I, and J represent the means (± SD) of three independent experiments. *p < 0.05; and n.s. not significant (*p* > 0.05) by one-way ANOVA. See also S6 Fig. The data for B-G and I-J are available in S3 Data. mESC, mouse embryonic stem cell; WT, wild-type.

Multiple lines of evidence indicated that the R595H-Trim71 specifically represses the translation of *Ctnnb1* mRNA. First, R595H-Trim71 mESCs specifically showed reduced levels of β-catenin with no change in *Ctnnb1* mRNA levels (Fig 4A–4C). Second, the association of Ctnnb1 mRNA with polysomes was specifically decreased in R595H-Trim71 mESCs (Fig 4D and 4E). Third, ectopic expression of R595H-Trim71 in WT mESCs reduced β-catenin levels without decreasing *Ctnnb1* mRNA levels (Fig 4H–4J).

In contrast, the protein levels and polysome association corresponding to *Lsd1* mRNA were specifically affected by R783H-Trim71 but not R595H-Trim71 (Fig 4A–4J). Overall, these data suggest that the two CH-associated Trim71 mutants repress the translation of different, ectopic mRNAs and that R595H-Trim71 specifically represses *Ctnnb1* mRNA translation.

Strikingly, we observed similar results upon ectopic expression of the corresponding human TRIM71 mutants in human embryonal carcinoma cells, suggesting that the regulation of *Ctnnb1* mRNA by R595H-Trim71 is conserved in human (S6 Fig versus Fig 4H–4J). Thus, similar to the regulation of *Lsd1* mRNA by R783H-Trim71, the R595H-Trim71-mediated regulation of *Ctnnb1* mRNA is conserved between mouse and human.

Collectively, these results support the notion that different Trim71 mutants regulate the translation of different, ectopic target mRNAs.

### Eliminating the ectopic R595H-Trim71:*Ctnnb1* mRNA interaction alleviates the mESC differentiation defects

**Fig 5:**
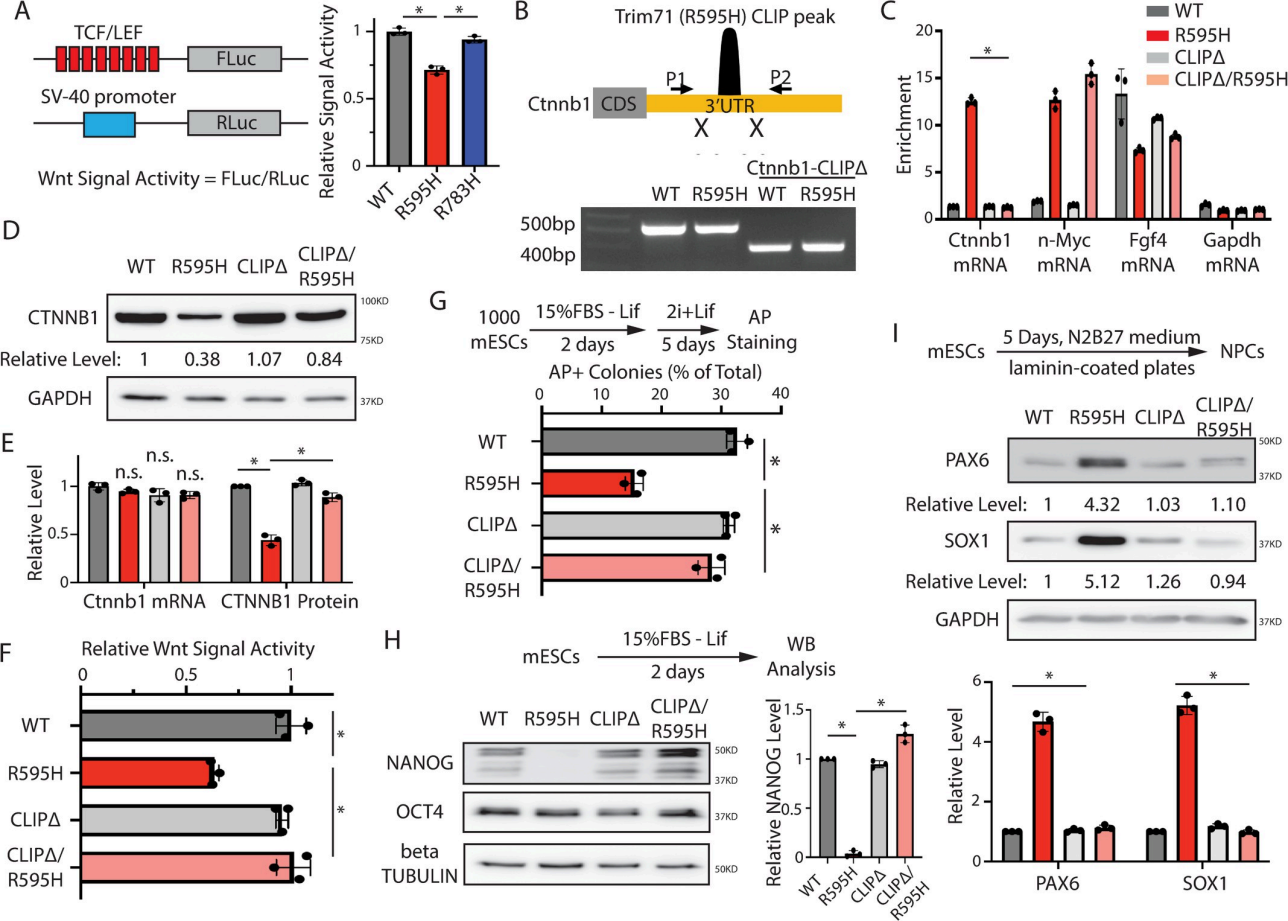
Disrupting the interaction between the *Ctnnb1* mRNA and the R595H Trim71 specifically alleviates the differentiation defects in the R595H mESCs. (**A**) Measuring the Wnt signaling activity in the WT and the two mutant mESCs using a luciferase report. The cells were cultured in the 15% FBS + Lif medium. (**B**) Deletion of the R595H Trim71 binding site in *Ctnnb1* mRNA’s 3′ UTR. (**C**) CLIP-RIP followed by qRT-PCR to examine mRNAs associated with the WT Trim71 and the R595H Trim71 in the WT, R595H, CLIPΔ, and R595H/CLIPΔ mESCs. The mRNA signals from the E14 mESCs were set as 1 for relative comparison. The cells were cultured in 2i + Lif medium. (**D**, **E**) Western blotting and qRT-PCR quantifications in the WT, R595H, CLIPΔ, and R595H/CLIPΔ mESCs. GAPDH and 18S rRNA were used for normalization in protein and mRNA quantifications, respectively. The cells were cultured in 2i + Lif medium. (**F**) Measuring the Wnt signaling activity in WT, R595H, CLIPΔ, and R595H/CLIPΔ mESCs using the luciferase assay. The cells were cultured in 15% FBS + Lif medium. (**G**) Exit pluripotency assay for mESCs. (**H**) Western blotting of pluripotency factors during the spontaneous differentiation of mESCs. (**I**) Western blotting of neural lineage markers during the directed neural differentiation. All the quantification results represent the means (± SD) of three independent experiments. *p < 0.05; and n.s. not significant (*p* > 0.05) by one-way ANOVA. See also S7 Fig. The data for A, C, and E-H are available in S4 Data. mESC, mouse embryonic stem cell; qRT-PCR, quantitative real-time PCR; WT, wild-type.

Because β-catenin is a key component in the Wnt signaling pathway, we determined whether reduced β-catenin levels impact Wnt signaling in R595H-Trim71 mESCs. Briefly, we measured Wnt signaling activity using a luciferase reporter whose expression is controlled by Wnt signaling response elements (TCF/LEF) (Fig 5A) [21]. Wnt signaling activity was reduced in R595H-Trim71 mESCs compared to WT and R783H-Trim71 mESCs (Fig 5A), suggesting that *Ctnnb1* mRNA is a functionally relevant target of R595H-Trim71.

To determine if dysregulation of *Ctnnb1* mRNA contributes to the differentiation defects of R595H-Trim71 mESCs, we deleted the R595H-Trim71 binding site in the 3′ UTR of *Ctnnb1* mRNA (termed “Ctnnb1 CLIPΔ”; Fig 5B). We verified that Ctnnb1-CLIPΔ specifically disrupted the interaction between *Ctnnb1* mRNA and R595H-Trim71 by CLIP-qRT-PCR (Fig 5C). Importantly, β-catenin levels and Wnt signaling activity were restored in R595H-Trim71 mESC expressing Ctnnb1 CLIPΔ, without an obvious alteration of *Ctnnb1* mRNA levels (Fig 5D–5F). This increase of β-catenin levels and Wnt signaling were specific to the R595H-Trim71 mESCs and were not observed upon expression of Ctnnb1 CLIPΔ in the WT mESCs (Fig 5D–5F).

Next, to determine if Ctnnb1 CLIPΔ also alleviates the stem cell and neural differentiation defects in the R595H-Trim71 mESCs, we performed the exit pluripotency assay and examined the expression of pluripotency factors during differentiation. Indeed, the rate of exit pluripotency and levels of Nanog during differentiation were restored in R595H-Trim71 mESCs expressing Ctnnb1 CLIPΔ (Fig 5G and 5H). Similarly, the levels of neural lineage specification factors Pax6 and Sox1 during directed neural differentiation were restored in R595H-Trim71 mESCs expressing Ctnnb1 CLIPΔ (Fig 5I). In contrast, Ctnnb1 CLIPΔ did not mitigate the differentiation defects in the R783H-Trim71 mESCs (S7 Fig). Additionally, Lsd1 CLIPΔ, which alleviated the differentiation defects in R783H-Trim71 mESCs [15], failed to rescue the defects in R595H-Trim71 mESCs (S7 Fig). Thus, the effects of each CH-associated Trim71 mutation on differentiation are target mRNA-specific.

Taken together, these results suggest that *Ctnnb1* mRNA is an important ectopic and functional target of the R595H-Trim71 and that decreased Wnt/β-catenin signaling is a major and specific cause of the differentiation defects s in R595H-Trim71 mESCs.

### Increasing β-catenin levels specifically alleviates the differentiation defects in R595H-Trim71 mESCs

Based on our findings so far, we hypothesized that increasing β-catenin levels would alleviate the differentiation defects in R595H-Trim71 mESCs.

**Fig 6:**
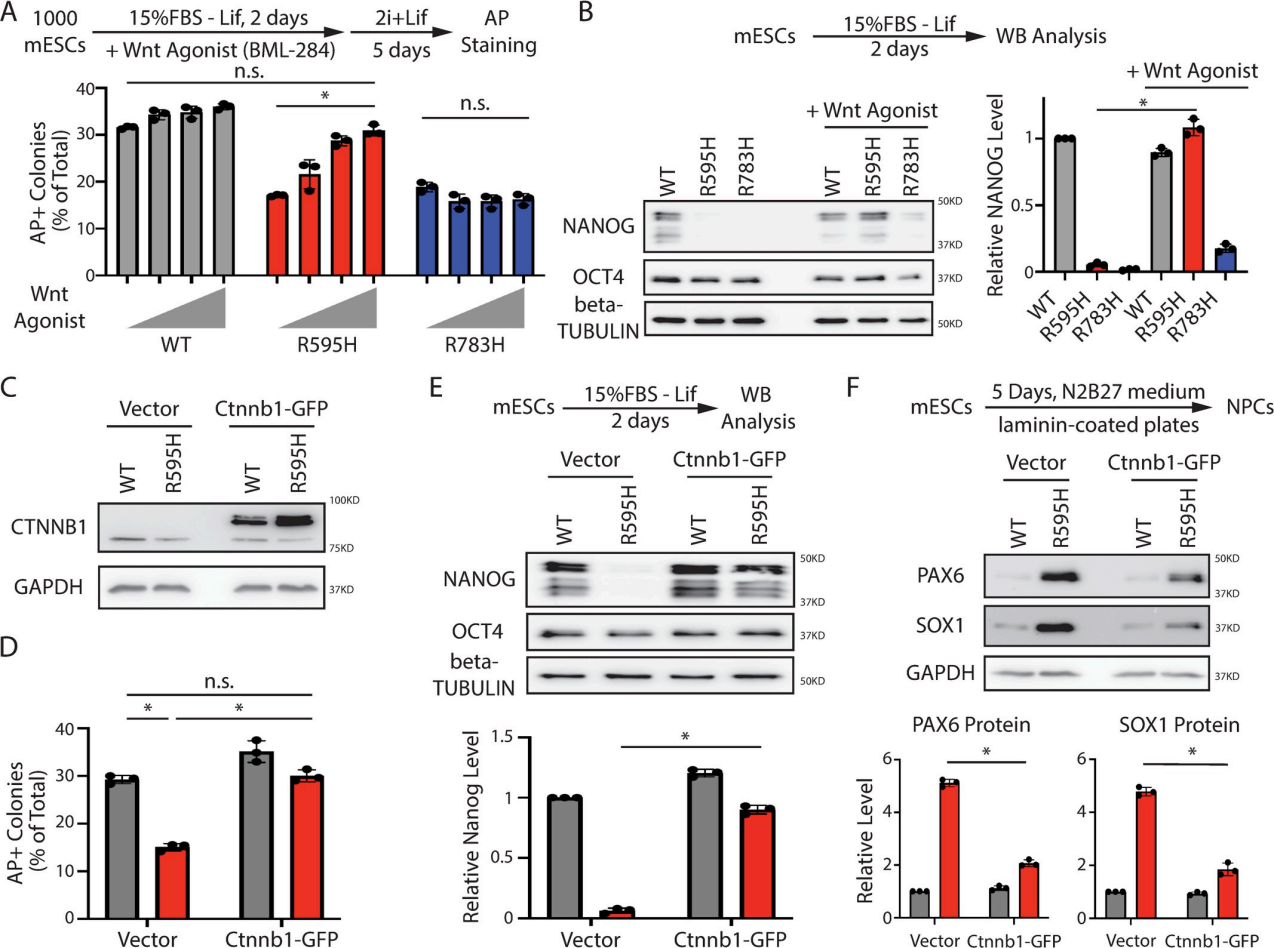
Enhancing the Wnt signaling specifically alleviates the differentiation defects in the R595H mESCs. (**A**) Exit pluripotency assay for mESCs in the presence BML-284. In the assay, 0, 85, 90, 95 nM BML-284 was used. (**B**) Western blotting of pluripotency factors during the spontaneous differentiation of mESCs. During the differentiation, 95 nM BML-284 was used. (**C**) Ectopic expression of Ctnnb1 in the WT and the R595H mESCs. The cells were cultured in the 2i + Lif medium. (**D**) Exit pluripotency assay for mESCs with the ectopically expressed Ctnnb1. (**E**) Western blotting of pluripotency factors during the spontaneous differentiation of mESCs with the ectopically expressed Ctnnb1. (**F**) Western blotting of neural lineage markers during the directed neural differentiation. All the quantification results represent the means (± SD) of three independent experiments. *p < 0.05; and n.s. not significant (*p* > 0.05) by one-way ANOVA. The data for A, B, and D-F are available in S5 Data. mESC, mouse embryonic stem cell; WT, wild-type.

To test this hypothesis, we treated R595H-Trim71 mESCs with a well-characterized Wnt signaling activator, BML-284 [22], to stabilize β-catenin. We found that R595H-Trim71 mESCs but not R783H-Trim71 mESCs treated with BML-284 showed a dose-dependent mitigation of the differentiation defects, as indicated by the exit pluripotency assay (Fig 6A). Treatment with this agonist also restored Nanog levels during spontaneous differentiation of R595H-Trim71 but not of R783H-Trim71 mESCs (Fig 6B). Unfortunately, we observed significant cell death during directed neural differentiation of WT and mutant mESCs in the presence of BML-284, suggesting that constitutive activation of the Wnt signaling is detrimental.

As an orthogonal approach, we increased β-catenin levels by ectopically expressing Ctnn1b-GFP (Fig 6C). The increased level of β-catenin alleviated the differentiation defects of R595H-Trim71 mESCs, as revealed by the restored rate of pluripotency exit (Fig 6D) and levels of Nanog during spontaneous differentiation (Fig 6E). Morever, during directed neural differentiation, the increased β-catenin levels had no effect on WT mESCs but mitigated the premature neuro-lineage commitment of R595H-Trim71 mESCs (Fig 6F).

Altogether, these results strongly suggest that decreased Wnt/β-catenin signaling specifically contributes to the stem cell differentiation defects in R595H-Trim71 mESCs.

### Identification of β-catenin target genes with decreased expression in R595H-Trim71 mESCs

**Fig 7:**
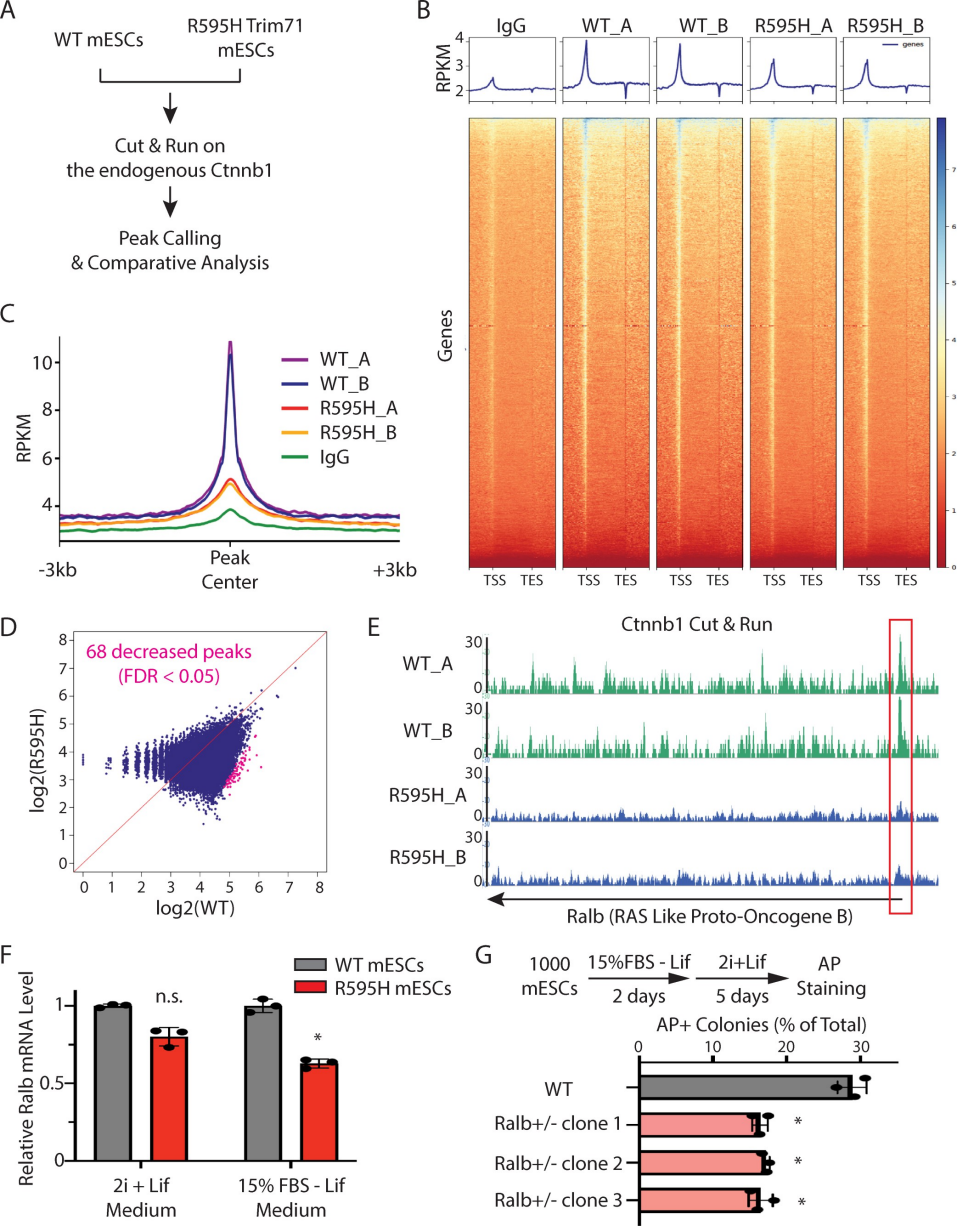
Identification of target genes impacted by the decreased Wnt signaling in the R595H mESCs. (**A**) A workflow for the Cut&Run analysis on the endogenous Ctnnb1 in the WT and the R595H mESCs. The cells were cultured in the 15% FBS + Lif medium for the Cut&Run analysis. (**B**) Metagene analysis of Ctnnb1 binding regions within 3 kb upstream of TSS and 3 kb downstream of TES. (**C**) Ctnnb1 peak signal intensity in the WT and the R595H mESCs. (**D**) Identification of Ctnnb1 peaks with differential intensity between the WT and the R595H mESCs. (**E**) Ctnnb1 Cut&Run signal in the Ralb locus. The red box indicated the region with decreased signaling intensity in the R595H mESCs. (**F**) qRT-PCR quantification of *Ralb* mRNA in the WT and the R595H mESCs cultured in the stemness (2i + Lif) and differentiating (15% FBS–Lif) conditions. 18S rRNA was used for quantification. (**G**) Exit pluripotency assay for the mESCs. In B, C, E, the A and B (e.g., WT_A and WT_B) are Cut&Run data from two biological replicates. The quantifications in F and G represent the means (± SD) of three independent experiments. *p < 0.05; and n.s. not significant (*p* > 0.05) by one-way ANOVA. See also S8 Fig. The data for F and G are available in S6 Data. FDR, false discovery rate; mESC, mouse embryonic stem cell; qRT-PCR, quantitative real-time PCR; RPKM, reads per kilobase per million mapped reads; TES, transcription end site; TSS, transcription start site; WT, wild-type.

To identify the target genes impacted by the decreased Wnt/β-catenin signaling in R595H-Trim71 mESCs, we determined the chromatin regions associated with endogenous β-catenin in WT and R595H-Trim71 mESCs. β-catenin does not directly bind DNA/chromatin but regulates target gene expression by functioning as a scaffold to recruit other transcriptional factors, such as TCF and LEF [21]. We considered that the formaldehyde crosslinking in the ChIP-seq may not efficiently trap all the β-catenin-associated chromatin regions, so we used the Cut&Run assay to identify target sites (Fig 7A). Cut&Run isolates the chromatin regions associated with the target protein through proximity-mediated nuclease cleavage without crosslinking [23]. Consistent with previous findings [24], β-catenin-associated chromatin regions (peaks) identified by Cut&Run were enriched around promoter regions in WT mESCs (Figs 7B and S8A). Moreover, β-catenin peaks were observed in a number of well-characterized target genes of the Wnt signaling pathway, as such Sp6 (S8B Fig and S3 Table), validating our approach.

Comparing β-catenin peaks from WT with those from R595H-Trim71 mESCs revealed a global decrease in their intensity in R595H-Trim71 mESCs (Fig 7B and 7C), consistent with attenuated Wnt signaling activity. Differential analysis revealed 68 chromatin regions, including 9 promoters, with a significant decrease of β-catenin association in R595H-Trim71 mESCs (Fig 7D and S3 Table), suggesting that those genes may be dysregulated. We focused on one such gene, *Ralb*, because the β-catenin peak in its promoter region was dramatically decreased in R595H-Trim71 cells (Fig 7E) and Ralb mRNA was significantly decreased during differentiation of R595H-Trim71 mESCs compared to WT mESCs (Fig 7F). Moreover, *Ralb* encodes a GTPase with implicated functions in stem cell biology [25]. To determine whether decreased Ralb can cause stem cell differentiation defects, we examined pluripotency exit of *Ralb* heterozygous mESCs. Similar to R595H-Trim71 mESCs, *Ralb* heterozygous mESCs also displayed an accelerated exit from pluripotency (Fig 7G). Collectively, these results argue that reduced *Ralb* expression due to decreased Wnt/β-catenin signaling may contribute to the differentiation defects in R595H-Trim71 mESCs.

### Dysregulated β-catenin underlies differentiation defects in mESCs with monoallelic R595H-Trim71

**Fig 8:**
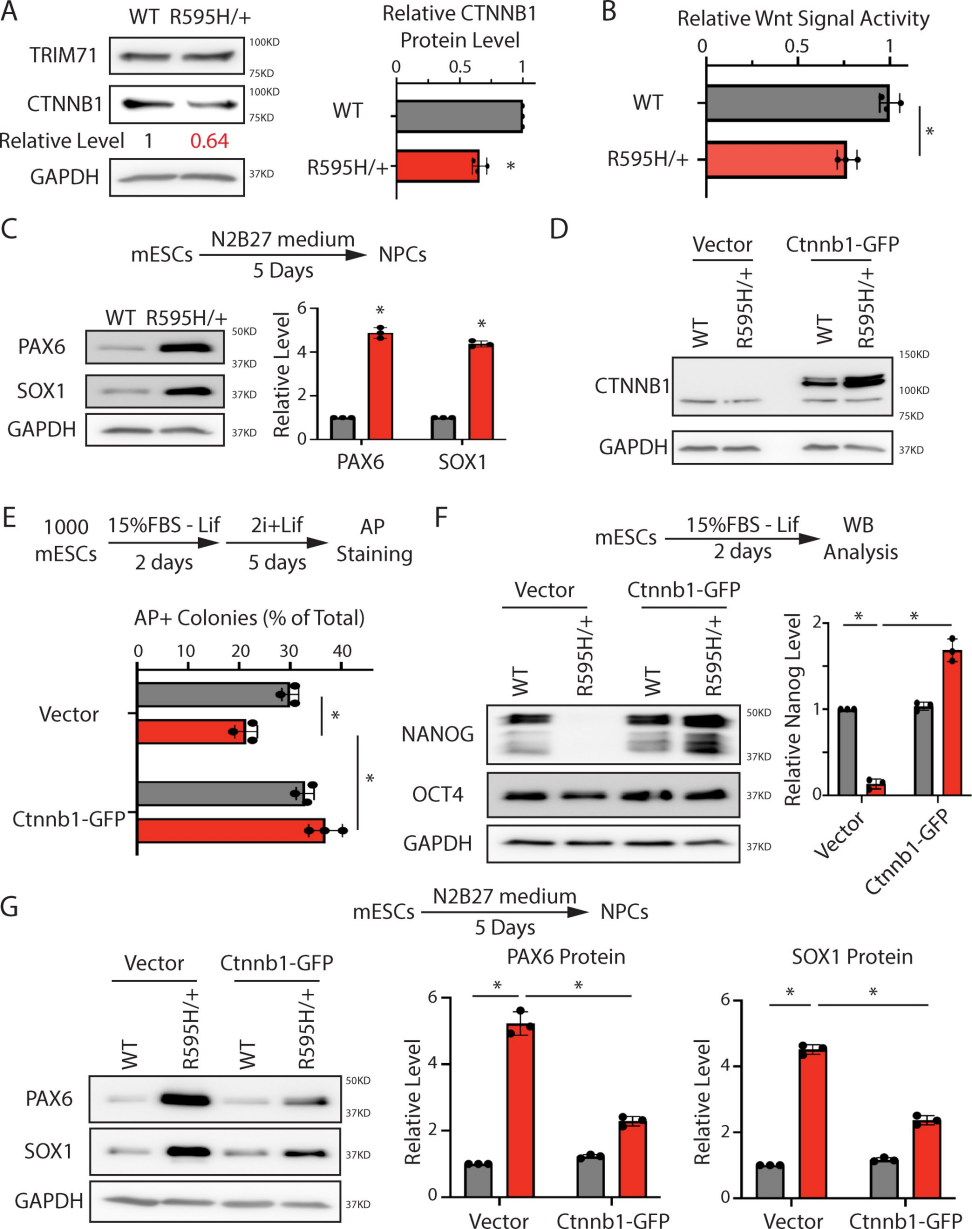
The down-regulated Ctnnb1 is critical for the differentiation defects in mESCs with monoallelic R595H mutation in Trim71. (**A**) Representative western blotting and quantification in the WT and the R595H/+ mESCs cultured in the 2i + Lif medium. (**B**) Measuring the Wnt signaling in the WT and the R595H/+ mESCs cultured in the 15% FBS + Lif medium using the luciferase assay. (**C**) Representative western blotting and quantification of neural lineage markers during the directed neural differentiation. (**D**) Western blotting in the WT and the R595H/+ mESCs cultured in the 2i + Lif medium with the ectopically expressed Ctnnb1. (**E**) Exit pluripotency assay for the WT and the R595H/+ mESCs with the ectopically expressed Ctnnb1. (**F**) Representative western blotting and quantification of pluripotency factors during the spontaneous differentiation of the WT and the R595H/+ mESCs with the ectopically expressed Ctnnb1. (**G**) Representative western blotting and quantification of neural lineage markers during the directed neural differentiation of the WT and the R595H/+ mESCs with the ectopically expressed Ctnnb1. All the quantification results represent the means (± SD) of three independent experiments. *p < 0.05; and n.s. not significant (*p* > 0.05) by one-way ANOVA. The data for A-C and E-G are available in S7 Data. mESC, mouse embryonic stem cell; WT, wild-type.

To evaluate whether the mechanistic insights we obtained using biallelic R595H mESCs are pathologically relevant, we examined mESCs with a monoallelic R595H mutation in Trim71 (R595H/+) (S1 Fig), which mimics the genetic setting of CH in humans. Similar to homozygous R595H-Trim71 mESCs, heterozygous R595H-Trim71 mESCs also had lower β-catenin levels (Fig 8A), decreased Wnt signaling activity (Fig 8B), and enhanced neural lineage commitment (Fig 8C) when compared to WT mESCs. Moreover, ectopic expression of β-catenin (Fig 8D) alleviated all the differentiation defects in heterozygous R595H-Trim71 mESCs, as indicated by the restored rate of pluripotency exit (Fig 8E), restored expression of Nanog during the spontaneous differentiation (Fig 8F), and restored expression of neural lineage markers during directed neural differentiation (Fig 8G). Thus, in a heterozygous setting mimicking CH, elevating β-catenin levels can mitigate the stem cell differentiation defects caused by the R595H mutation in Trim71.

## Discussion

We discovered that although two different CH-associated mutations in Trim71 lead to similar differentiation defects in mESCs, they do so via distinct mechanisms, namely, the two Trim71 mutants ectopically bind to and translationally repress different mRNAs. We determined that R595H-Trim71 disrupts mESC differentiation because it specifically represses the translation of *Ctnnb1* mRNA, leading to attenuated Wnt/β-catenin signaling. These results strongly suggest that dysregulation of the Wnt/β-catenin signaling pathway at the posttranscriptional level is important for the pathogenesis of CH mediated by the R595H mutation in Trim71. Moreover, our findings indicate the two CH-associated mutations are gain-of-function mutations, consistent with monoallelic mutations in Trim71 causing CH in human.

### CH-associated mutations in Trim71

Recent studies in mice showed that CH-associated mutations result in prenatal hydrocephalus in mice [14], indicating conserved pathogenesis. Detailed phenotypic analysis of the mouse models established that defects in neurogenesis and differentiation underlie the abnormal accumulation of cerebrospinal fluid in the brain. This work also showed that Trim71 KO, Trim71^R595H/R595H^, and Trim71^R595H/+^ mESCs were less proliferative than WT-mESCs upon N2B27-induced neural differentiation [14].

Our findings complement this study [14]. Instead of focusing on the generation of mature neurons from neural progenitor/stem cells as the recent study did [14], our study focused on the neural lineage specification from mESCs (i.e., from mESCs to neural progenitor/stem cells). We found that heterozygous and homozygous R595H-Trim71 mESCs and R783H-Trim71 mESCs, but not Trim71-KO mESCs, were more prone spontaneous differentiation, particularly into the neural ectoderm lineage, revealing a novel gain-of-function defect. This defect is not due to altered proliferation, because heterozygous and homozygous R595H-Trim71 mESCs and R783H-Trim71 mESCs displayed similar proliferation as WT-mESCs under self-renewal and spontaneous differentiation conditions. Together, our findings and the recent studies on mouse models with CH-associated mutations suggest that the CH-associated mutations in Trim71 cause differentiation defects in multiple and potentially continuous developmental processes, including germ layer specification and neurogenesis. This is consistent with specific and abundant Trim71 expression in embryonic stem cells, neural progenitor cells, and the developing brain.

Monoallelic mutations in Trim71 result in CH in human [4], suggesting that those mutations could be gain-of-function mutations or, alternatively, that CH could arise from haploinsufficiency of Trim71 due to the loss-of-function mutations. Trim71^R595H/+^ mice and mice with neuroprogenitor-specific KO of Trim71 survive until birth, with approximately 16% to 25% displaying ventriculomegaly and progressive macrocephaly [14], whereas Trim71^R595H/R595H^ and Trim71-KO mice are embryonic lethal and displayed defects in neural tube formation [14]. In addition, R595H-Trim71 has reduced binding to mRNA targets of WT-Trim71 in mESC, as we also found here. However, heterozygous Trim71^−/+^ mice are viable, fertile, and lack abnormalities [26], suggesting that R595H is not a null allele. Moreover, our results here on the R595H mutation, together with our recent findings on the R783H mutation [15], argue that these two mutations are gain-of-functions because they (1) cause Trim71 to bind different, ectopic target mRNAs and (2) induce specific differentiation defects that are not observed with Trim71-KO mESCs. These results can help explain the phenotypic differences between Trim71 heterozygous mice and mice with CH-associated mutations in Trim71.

Our studies and others showed that R595H-Trim71 and R783H-Trim71 have reduced binding to many WT-Trim71 target mRNAs. However, our results indicate that each of the two CH-associated Trim71 mutants also bind a new set of target mRNAs. Consistent with this finding, the RNA binding results from the recent study [14] indicated that although the R595H Trim71 mutant binds less RNA compared to the WT Trim71, the amount of the RNA bound by this mutant is still higher than the background signal and comparable to that of the positive control (DHX36) (Fig 7F in [14]), suggesting the mutant Trim71 can still bind and potentially regulate target mRNAs. Our recent study on R783H-Trim71 [15] and the results in this study on R595H-Trim71 further showed that the differentiation defects in the two mutant mESCs arise from the repression of new target mRNAs, specifically regulated by each of gain-of-function Trim71 mutant.

### Target mRNA recognition by Trim71 and its mutants

Trim71 interacts with RNA targets through the NHL domain [7]. In vitro RNA-binding studies revealed that the binding to the NHL domain is mediated by an RNA stem-loop structure rather than primary sequence motifs [18]. The results from our CLIP-seq analysis revealed that in mESCs, the two CH-associated mutations in the NHL domain do not abolish but rather almost completely change the mRNAs bound by Trim71 (Fig 2B). Interestingly, however, similar stem-loop secondary structures are overrepresented in the RNA regions bound by the WT or mutant Trim71 (Fig 2C). These observations raise the intriguing questions: (a) how Trim71 recognizes and binds its target RNAs; (b) how the CH-associated mutations in Trim71 alter its target recognition. Interestingly, the CH-associated mutations are localized on the surface, but not in the deep region of the RNA-binding pocket of the NHL domain of Trim71, as indicated by both the AlphaFold prediction and recent crystal structures of the NHL domain of Trim71 [27]. This structural information provides an explanation on the observation that among the enriched motifs from the WT Trim71 and the two Trim71 mutants, the core stem-loop region (regions close to H in Fig 2C) are highly similar, while the trailing regions (the ends of the motifs) display increased variation. We anticipate that future structural studies of Trim71:RNA complexes will reveal how this developmentally important and disease-relevant RBP recognizes its target RNAs.

### The Wnt/β-catenin pathway in CH

The Wnt/β-catenin signaling pathway plays important roles in diverse developmental processes [28]. Our results revealed that R595H-Trim71 attenuates this pathway by repressing the translation of *Ctnnb1* mRNA and reducing the levels of β-catenin, resulting in stem cell differentiation defects. Intriguingly, it was recently shown that deletion of β-catenin in neural stem/progenitor cells also leads to CH in mice [29]. These observations collectively argue that Trim71 mutations can cause CH pathogenesis due to dysregulation of Wnt/β-catenin signaling. β-catenin does not directly bind to DNA/chromatin but functions as a scaffold to recruit other transcription factors to modulate gene expression [21], therefore identifying its direct targets is challenging. Here, we used Cut&Run followed by sequencing to identify 68 genomic regions with significantly decreased β-catenin association in R595H-Trim71 mutant mESCs. We showed that *Ralb* has decreased β-catenin association in R595H-Trim71 mutant mESCs and that reduced expression of *Ralb* also leads to differentiation defects. We anticipate that uncovering additional Wnt/β-catenin target genes that are dysregulated in R595H-Trim71 mESCs will reveal new insights into CH pathogenesis.

## Materials and methods

All the antibodies, oligonucleotides, and plasmids used in this study are listed in S4 Table.

### Cell culture

All the mESCs were derived from the ES-E14TG2a line (ATCC CRL-1821) and were cultured in either 15% FBS + Lif or 2i + Lif medium on 0.5% gelatin-coated plates at 37°C with 5% CO2.

### CRISPR-mediated genome editing in mESCs

sgRNA-expressing vector and donor oligo/plasmid were cotransfected into mESCs using Fugene6. Transfected cells were single-cell sorted to 96-well plates, 48 hours posttransfection. The desired clones were selected based on the PCR genotyping, Sanger sequencing, and western blot analysis.

### Western blot analysis

Total proteins were extracted by RIPA buffer, quantified using BCA assay kit, and then resolved by SDS-PAGE gels. Western blotting was performed using a BlotCycler (Precision Biosystems) with the indicated antibodies. The signals on the membranes were generated with the Western ECL substrate (Bio-Rad) and imaged with an ImageQuant LAS 500 instrument (GE Healthcare).

### RNA isolation and RT-qPCR

RNA was isolated using Trizol with DNase1 treatment and then reverse transcribed into cDNA using random hexamers and Superscript2 reverse transcriptase. Quantitative real-time PCR was performed in triplicate for each sample by CFX96 real-time PCR detection system (Bio-Rad) with SsoAdvanced Universal SYBR Green Supermix (Bio-Rad).

### Assays for mESC self-renewal and differentiation

The colony formation assay and the exit pluripotency assay were performed as described previously [8]. For the monolayer differentiation, 20,000 mESCs were plated on gelatinized-6 well plate in the differentiation medium. Cells were harvested at day 2 for western blot analysis. The directed neural differentiation was performed as described previously [17].

### Immunofluorescence staining

For immunofluorescence staining, cells cultured on chamber sliders were fixed with 4% PFA for 20 minutes at room temperature and then permeabilized with 0.2% Triton X-100 in PBS. Cells were blocked with 3% BSA in PBS and subjected to staining with the indicated antibodies diluted in blocking solution supplemented with 0.1% Tween-20 for 2 hours at room temperature. Cells were then washed and stained with Hoechst Staining Dye for 10 minutes and then mounted with mounting medium. The images were acquired by a Nikon Eclipse fluorescence microscope and analyzed by ImageJ.

### Luciferase reporter assay

The luciferase assays were performed using the Dual-Glo Luciferase Assay System (Promega) on a GloMax 20/20 Luminometer (Promega).

### Polysome analysis

Polysome analysis was performed using the methods described previously [30].

### CLIP-seq, RNA-seq, Cut&Run, and computational analysis

CLIP-seq and peaking calling was performed using the methods described previously [8,31]. Poly(A)+ RNA was used for standard RNA-seq. The Cut&Run assay was performed using the CUT&RUN Assay Kit (Cell Signaling Technology, Cat#86652) in accordance with the provided protocol. To identify the peaks form the sequencing data, the raw reads were mapped to the mouse mm10 genome assembly using Bowtie2 (v2.4.5) with options:—local—very-sensitive-local—no-unal—no-mixed—no-discordant—phred33 -I 10 -X 700. Then, reads that mapped to the mitochondrial DNA were removed. PCR duplicates were removed by Picard (v.2.26.11), and peak calling was performed using SEACR (v1.3) in “stringent” mode. Differential binding peaks were identified with DiffBind (v3.2.7) with FDR cutoff of 0.05, and peaks were annotated using ChIPSeeker (v1.28.3). Motif discovery was performed using HOMER (v4.10). The high-throughput sequencing datasets generated in this study are available at GEO: GSE201945.

## Supporting information

S1 Fig Generation of the CH-associated mutations in FLAG-Trim71 mESCs. (**A**) The locations of the two CH-associated in Trim71. (**B**) Outline of the genome editing procedure. (**C**) Work flow of generating the two CH-associated mutations in mESCs. (**D**) Sanger sequencing results verifying the R595H mutation in mESCs. (**E**) Sanger sequencing results verifying the R783H mutation in mESCs. CH, congenital hydrocephalus; mESC, mouse embryonic stem cell.
(EPS)

S2 Fig The CH-associated mutations in Trim71 do not impact mESC self-renewal. The mESCs were cultured in 15% FBS + Lif for 7 days, and the resultant colonies were fixed and stained for AP. The colony morphology and AP intensity were evaluated through microscopy. To determine the percentage of undifferentiated colonies, 100–200 colonies were examined each time. The results represent the means (± SD) of three independent experiments. One-way ANOVA was used to determine the significance of the difference, n.s. not significant (*p* > 0.05). The data are available in S8 Data. CH, congenital hydrocephalus; mESC, mouse embryonic stem cell.
(EPS)

S3 Fig The CH-associated mutations in Trim71 does not change the proliferation and apoptosis of mESCs. (**A**) Proliferation of mESCs under the stemness condition. The cells were cultured in the 2i + Lif medium, and the cell proliferation was monitored by the CellTiter 96 AQueous One Solution Cell Proliferation Assay (Promega). (**B**) Proliferation of mESCs under the differentiating condition. The cells were cultured in the 15% FBS—Lif medium, and the cell proliferation was monitored by the CellTiter 96 AQueous One Solution Cell Proliferation Assay (Promega). (**C**) Apoptosis of mESCs under the stemness condition. The cells were cultured in the 2i + Lif medium, and the cellular apoptotic state was monitored by annexin-V and PI staining followed by flow cytometry analysis. (**D**) Apoptosis of mESCs under the differentiating condition. The cells were cultured in the 2i + Lif medium, and the cellular apoptotic state was monitored by annexin-V and PI staining followed by flow cytometry analysis. The results represent the means (± SD) of three independent experiments. *p < 0.05, n.s. not significant (*p* > 0.05) by one-way ANOVA. The data for A-D are available in S9 Data. CH, congenital hydrocephalus; mESC, mouse embryonic stem cell.
(EPS)

S4 Fig The mESCs with CH-associated mutations are more prone differentiate into the ectoderm lineage during EB formation. 18S rRNA was used for the normalization in the gene quantification by qRT-PCR. The qRT-PCR results represent the means (± SD) of three independent experiments. *p < 0.05, n.s. not significant (*p* > 0.05) by one-way ANOVA. The data are available in S10 Data. CH, congenital hydrocephalus; EB, embryoid body; mESC, mouse embryonic stem cell; qRT-PCR, quantitative real-time PCR.
(EPS)

S5 Fig Transcriptome profiling in the WT and the R595H mESCs. (**A**) Work flow of the RNA-seq analysis. (**B**) Differentially expressed genes in the WT and R595H mESCs. (**C**) Expression of the target RNAs of the R595H Trim71 mutant in the WT and the R595H mESCs. The differentially expressed genes were identified by the edgeR package. The expression level of each gene is the average of the three biological replicates. CPM, counts per million reads; mESC, mouse embryonic stem cell; WT, wild-type.
(EPS)

S6 Fig Regulation of the target mRNAs by the CH-associated Trim71 mutants is conserved between mouse and human. A representative western blotting the NCCIT cells expressing an empty vector, HA-hTRIM71, HA-hTRIM71(R608H), and HA-hTrim71(R796H). GAPDH was used for normalization in the quantification of Lsd1 protein levels, and 18S rRNA was used for normalization in the qRT-PCR quantification of Lsd1 mRNA levels. The quantification results represent the means (± SD) of three independent experiments. *p < 0.05, n.s. not significant (*p* > 0.05) by one-way ANOVA. CH, congenital hydrocephalus; qRT-PCR, quantitative real-time PCR.
(EPS)

S7 Fig Inhibition of the interaction between *Ctnnb1* mRNA and the R595H Trim71 can only alleviate the differentiation defects in the R595H mESCs, but not in the R783H mESCs. The exit pluripotency assay was performed on the indicated mESCs, and the resultant colonies were fixed and stained for AP. The colony morphology and AP intensity were evaluated through microscopy. To determine the percentage of undifferentiated colonies, 100–200 colonies were examined each time. The results represent the means (± SD) of three independent experiments. One-way ANOVA was used to determine the significance of the difference, n.s. not significant (*p* > 0.05). The data are available in S11 Data. mESC, mouse embryonic stem cell.
(EPS)

S8 Fig Identification of genomic regions associated with Ctnnb1 in mESCs. (**A**) Distribution of the Ctnnb1-associated genomic regions in the mouse genome. Only the genomic regions identified in two independent Cut&Run assays were retained for the analysis. (**B**) Cut&Run and ChIP-seq data at the Sp5 locus, a classical target gene of the Wnt/beta-Catenin signaling. The ChIP-seq data were from GSE131119 of Gene Expression Omnibus. The data for A are available in S12 Data. mESC, mouse embryonic stem cell.
(EPS)

S1 Table CLIP-seq peaks from the R595H Trim71 in mESCs. (XLSX)

S2 Table Selected candidate genes from the common targets of the R595H Trim71 and the R783H Trim71, and selected candidate genes specific to the R595H Trim71. (XLSX)

S3 Table Ctnnb1 binding regions determined by Cut&Run in mESCs. (CSV)

S4 Table Antibodies, cell lines, oligoes, plasmids, and chemicals used in this study. (XLSX)

S1 Raw Images Original images for blots and gels. (PDF)

S1 Data Numeric data for Fig 1B, 1C, 1E, 1G and 1H. (XLSX)

S2 Data Numeric data for Fig 2E. (XLSX)

S3 Data Numeric data for Fig 4B–4G, 4I and 4J. (XLSX)

S4 Data Numeric data for Fig 5A, 5C and 5E–5H. (XLSX)

S5 Data Numeric data for Fig 6A, 6B and 6D–6F. (XLSX)

S6 Data Numeric data for Fig 7F and 7G. (XLSX)

S7 Data Numeric data for Fig 8A–8C and 8E–8G. (XLSX)

S8 Data Numeric data for S2 Fig. (XLSX)

S9 Data Numeric data for S3 Fig. (XLSX)

S10 Data Numeric data for S4 Fig. (XLSX)

S11 Data Numeric data for S7 Fig. (XLSX)

S12 Data Numeric data for S8A Fig. (XLSX)

## Abbreviations

CH: congenital hydrocephalus
CLIP-seq: crosslinking immunoprecipitation and sequencing
EB: embryoid body
KO: knockout
mESC: mouse embryonic stem cell
qRT-PCR: quantitative real-time PCR
RBP: RNA-binding protein
WT: wild-type

## References

[1] Tully HM, Dobyns WB. Infantile hydrocephalus: a review of epidemiology, classification and causes. Eur J Med Genet. 2014;57(8):359–68. Epub 2014/06/17. doi: 10.1016/j.ejmg.2014.06.002 ; PubMed Central PMCID: PMC4334358. 24932902 PMC4334358

[2] Hanak BW, Bonow RH, Harris CA, Browd SR. Cerebrospinal Fluid Shunting Complications in Children. Pediatr Neurosurg. 2017;52(6):381–400. Epub 2017/03/02. doi: 10.1159/000452840 ; PubMed Central PMCID: PMC5915307. 28249297 PMC5915307

[3] Jin SC, Dong W, Kundishora AJ, Panchagnula S, Moreno-De-Luca A, Furey CG, et al. Exome sequencing implicates genetic disruption of prenatal neuro-gliogenesis in sporadic congenital hydrocephalus. Nat Med. 2020;26(11):1754–65. Epub 2020/10/21. doi: 10.1038/s41591-020-1090-2 ; PubMed Central PMCID: PMC7871900. 33077954 PMC7871900

[4] Furey CG, Choi J, Jin SC, Zeng X, Timberlake AT, Nelson-Williams C, et al. De Novo Mutation in Genes Regulating Neural Stem Cell Fate in Human Congenital Hydrocephalus. Neuron. 2018;99(2):302–14 e4. Epub 2018/07/10. doi: 10.1016/j.neuron.2018.06.019 ; PubMed Central PMCID: PMC7839075. 29983323 PMC7839075

[5] Kahle KT, Kulkarni AV, Limbrick DD Jr., Warf BC. Hydrocephalus in children. Lancet. 2016;387(10020):788–99. Epub 2015/08/11. doi: 10.1016/S0140-6736(15)60694-8 . 26256071

[6] Ecsedi M, Grosshans H. LIN-41/TRIM71: emancipation of a miRNA target. Genes Dev. 2013;27(6):581–9. Epub 2013/03/21. doi: 10.1101/gad.207266.112 ; PubMed Central PMCID: PMC3613605. 23512656 PMC3613605

[7] Connacher RP, Goldstrohm AC. Molecular and biological functions of TRIM-NHL RNA-binding proteins. Wiley Interdiscip Rev RNA. 2021;12(2):e1620. Epub 2020/08/02. doi: 10.1002/wrna.1620 ; PubMed Central PMCID: PMC7855385. 32738036 PMC7855385

[8] Liu Q, Chen X, Novak MK, Zhang S, Hu W. Repressing Ago2 mRNA translation by Trim71 maintains pluripotency through inhibiting let-7 microRNAs. Elife. 2021;10. Epub 2021/02/19. doi: 10.7554/eLife.66288 ; PubMed Central PMCID: PMC7906602. 33599613 PMC7906602

[9] Welte T, Tuck AC, Papasaikas P, Carl SH, Flemr M, Knuckles P, et al. The RNA hairpin binder TRIM71 modulates alternative splicing by repressing MBNL1. Genes Dev. 2019;33(17–18):1221–35. Epub 2019/08/03. doi: 10.1101/gad.328492.119 ; PubMed Central PMCID: PMC6719626. 31371437 PMC6719626

[10] Aeschimann F, Kumari P, Bartake H, Gaidatzis D, Xu L, Ciosk R, et al. LIN41 Post-transcriptionally Silences mRNAs by Two Distinct and Position-Dependent Mechanisms. Mol Cell. 2017;65(3):476–89 e4. Epub 2017/01/24. doi: 10.1016/j.molcel.2016.12.010 . 28111013

[11] Worringer KA, Rand TA, Hayashi Y, Sami S, Takahashi K, Tanabe K, et al. The let-7/LIN-41 pathway regulates reprogramming to human induced pluripotent stem cells by controlling expression of prodifferentiation genes. Cell Stem Cell. 2014;14(1):40–52. Epub 2013/11/19. doi: 10.1016/j.stem.2013.11.001 ; PubMed Central PMCID: PMC3982312. 24239284 PMC3982312

[12] Loedige I, Gaidatzis D, Sack R, Meister G, Filipowicz W. The mammalian TRIM-NHL protein TRIM71/LIN-41 is a repressor of mRNA function. Nucleic Acids Res. 2013;41(1):518–32. Epub 2012/11/06. doi: 10.1093/nar/gks1032 ; PubMed Central PMCID: PMC3592402. 23125361 PMC3592402

[13] Chang HM, Martinez NJ, Thornton JE, Hagan JP, Nguyen KD, Gregory RI. Trim71 cooperates with microRNAs to repress Cdkn1a expression and promote embryonic stem cell proliferation. Nat Commun. 2012;3:923. Epub 2012/06/28. doi: 10.1038/ncomms1909 ; PubMed Central PMCID: PMC3518406. 22735451 PMC3518406

[14] Duy PQ, Weise SC, Marini C, Li XJ, Liang D, Dahl PJ, et al. Impaired neurogenesis alters brain biomechanics in a neuroprogenitor-based genetic subtype of congenital hydrocephalus. Nat Neurosci. 2022;25(4):458–73. Epub 2022/04/06. doi: 10.1038/s41593-022-01043-3 . 35379995 PMC9664907

[15] Liu Q, Novak MK, Pepin RM, Maschhoff KR, Worner K, Chen X, et al. A congenital hydrocephalus-causing mutation in Trim71 induces stem cell defects via inhibiting *Lsd1* mRNA translation. EMBO Rep. 2022:e55843. Epub 2022/12/28. doi: 10.15252/embr.202255843 36573342 PMC9900330

[16] Betschinger J, Nichols J, Dietmann S, Corrin PD, Paddison PJ, Smith A. Exit from pluripotency is gated by intracellular redistribution of the bHLH transcription factor Tfe3. Cell. 2013;153(2):335–47. Epub 2013/04/16. doi: 10.1016/j.cell.2013.03.012 ; PubMed Central PMCID: PMC3661979. 23582324 PMC3661979

[17] Mulas C, Kalkan T, von Meyenn F, Leitch HG, Nichols J, Smith A. Defined conditions for propagation and manipulation of mouse embryonic stem cells. Development. 2019;146(6). Epub 2019/03/28. doi: 10.1242/dev.173146 ; PubMed Central PMCID: PMC6451320. 30914406 PMC6451320

[18] Kumari P, Aeschimann F, Gaidatzis D, Keusch JJ, Ghosh P, Neagu A, et al. Evolutionary plasticity of the NHL domain underlies distinct solutions to RNA recognition. Nat Commun. 2018;9(1):1549. Epub 2018/04/21. doi: 10.1038/s41467-018-03920-7 ; PubMed Central PMCID: PMC5908797. 29674686 PMC5908797

[19] Ying QL, Wray J, Nichols J, Batlle-Morera L, Doble B, Woodgett J, et al. The ground state of embryonic stem cell self-renewal. Nature. 2008;453(7194):519–23. Epub 2008/05/24. doi: 10.1038/nature06968 ; PubMed Central PMCID: PMC5328678. 18497825 PMC5328678

[20] Nusse R. Wnt signaling and stem cell control. Cell Res. 2008;18(5):523–7. Epub 2008/04/09. doi: 10.1038/cr.2008.47 . 18392048

[21] MacDonald BT, Tamai K, He X. Wnt/beta-catenin signaling: components, mechanisms, and diseases. Dev Cell. 2009;17(1):9–26. Epub 2009/07/22. doi: 10.1016/j.devcel.2009.06.016 ; PubMed Central PMCID: PMC2861485. 19619488 PMC2861485

[22] Liu J, Wu X, Mitchell B, Kintner C, Ding S, Schultz PG. A small-molecule agonist of the Wnt signaling pathway. Angew Chem Int Ed Engl. 2005;44(13):1987–90. Epub 2005/02/23. doi: 10.1002/anie.200462552 . 15724259

[23] Skene PJ, Henikoff S. An efficient targeted nuclease strategy for high-resolution mapping of DNA binding sites. Elife. 2017;6. Epub 2017/01/13. doi: 10.7554/eLife.21856 ; PubMed Central PMCID: PMC5310842. 28079019 PMC5310842

[24] Guo Q, Kim A, Li B, Ransick A, Bugacov H, Chen X, et al. A beta-catenin-driven switch in TCF/LEF transcription factor binding to DNA target sites promotes commitment of mammalian nephron progenitor cells. Elife. 2021;10. Epub 2021/02/16. doi: 10.7554/eLife.64444 ; PubMed Central PMCID: PMC7924951. 33587034 PMC7924951

[25] Johansson J, Naszai M, Hodder MC, Pickering KA, Miller BW, Ridgway RA, et al. RAL GTPases Drive Intestinal Stem Cell Function and Regeneration through Internalization of WNT Signalosomes. Cell Stem Cell. 2019;24(4):592–607 e7. Epub 2019/03/12. doi: 10.1016/j.stem.2019.02.002 ; PubMed Central PMCID: PMC6459002. 30853556 PMC6459002

[26] Cuevas E, Rybak-Wolf A, Rohde AM, Nguyen DT, Wulczyn FG. Lin41/Trim71 is essential for mouse development and specifically expressed in postnatal ependymal cells of the brain. Front Cell Dev Biol. 2015;3:20. Epub 2015/04/18. doi: 10.3389/fcell.2015.00020 ; PubMed Central PMCID: PMC4382986. 25883935 PMC4382986

[27] Apirat Chaikuad RZ, Tredupa C, Knapp S. Comparative structural analyses of the NHL domains from the human E3 ligase TRIM-NHL family. IUCrJ. 2022;9(6). doi: 10.1107/S2052252522008582 36381143 PMC9634614

[28] Steinhart Z, Angers S. Wnt signaling in development and tissue homeostasis. Development. 2018;145(11). Epub 2018/06/10. doi: 10.1242/dev.146589 . 29884654

[29] Ma L, Du Y, Xu X, Feng H, Hui Y, Li N, et al. beta-Catenin Deletion in Regional Neural Progenitors Leads to Congenital Hydrocephalus in Mice. Neurosci Bull. 2022;38(1):81–94. Epub 2021/08/31. doi: 10.1007/s12264-021-00763-z ; PubMed Central PMCID: PMC8782971. 34460072 PMC8782971

[30] Zhang X, Chen X, Liu Q, Zhang S, Hu W. Translation repression via modulation of the cytoplasmic poly(A)-binding protein in the inflammatory response. Elife. 2017;6. Epub 2017/06/22. doi: 10.7554/eLife.27786 ; PubMed Central PMCID: PMC5507668. 28635594 PMC5507668

[31] Chen X, Castro SA, Liu Q, Hu W, Zhang S. Practical considerations on performing and analyzing CLIP-seq experiments to identify transcriptomic-wide RNA-protein interactions. Methods. 2019;155:49–57. Epub 2018/12/12. doi: 10.1016/j.ymeth.2018.12.002 ; PubMed Central PMCID: PMC6387833. 30527764 PMC6387833

