# Supplementary material for: Different congenital hydrocephalus–associated mutations in Trim71 impair stem cell differentiation via distinct gain-of-function mechanisms
Source: PLoS Biol. 2023 Feb 9;21(2):e3001947. doi: 10.1371/journal.pbio.3001947 (PMC9910693; doi:10.1371/journal.pbio.3001947)
Supplement: S1 Raw Images — (PDF) [file pbio.3001947.s013.pdf]

Figure 1A

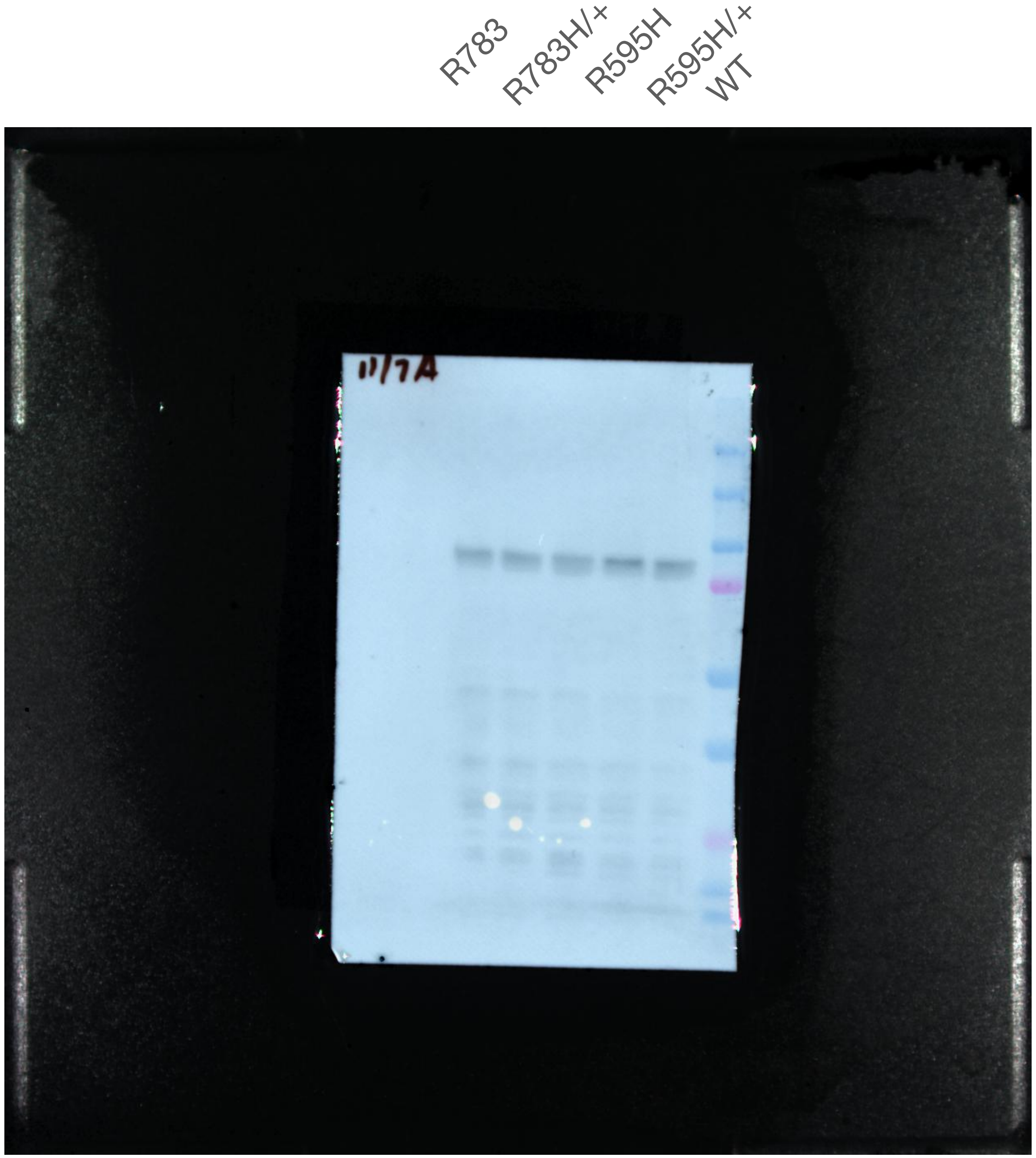

TRIM71

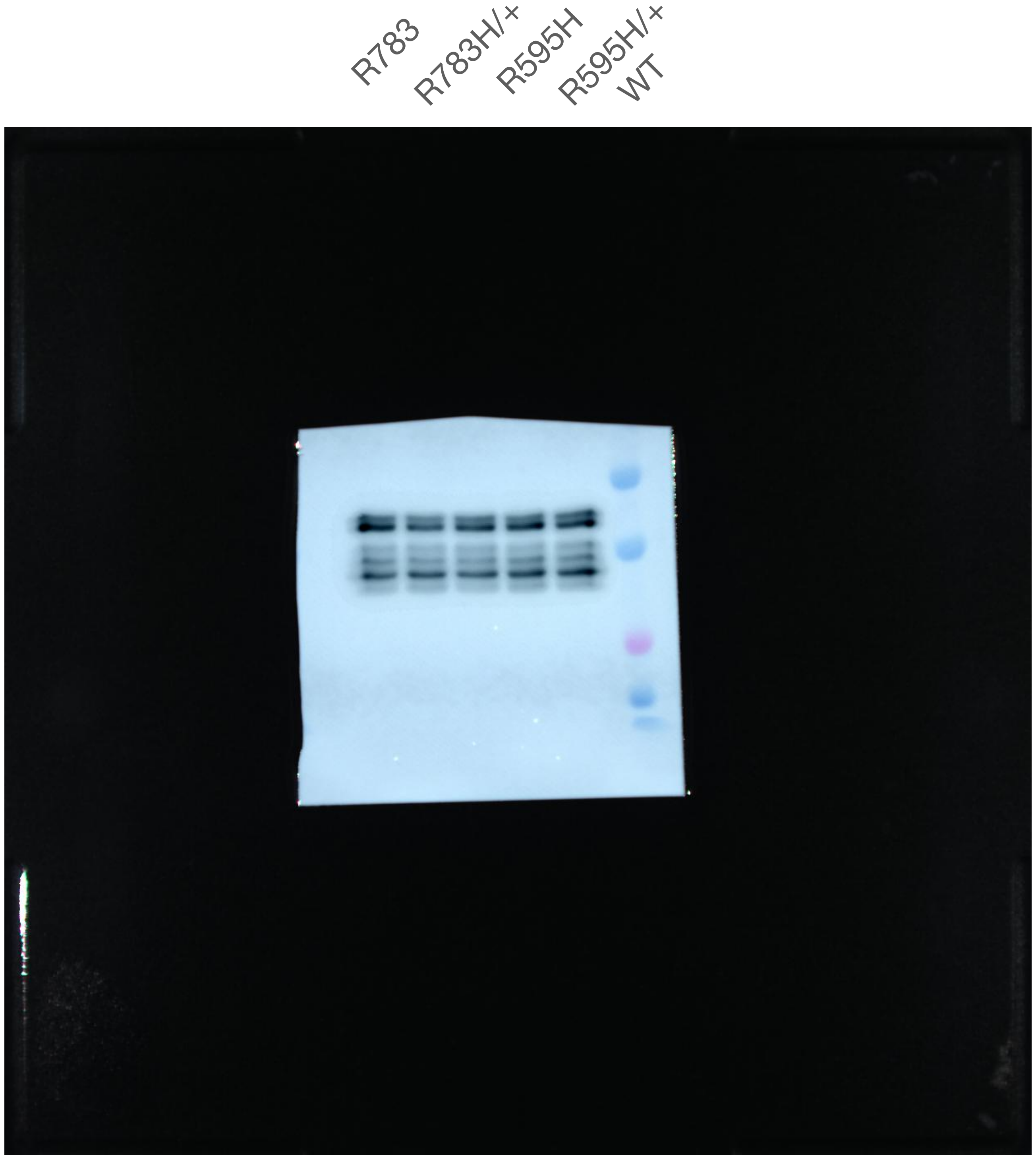

NANOG

Figure 1A

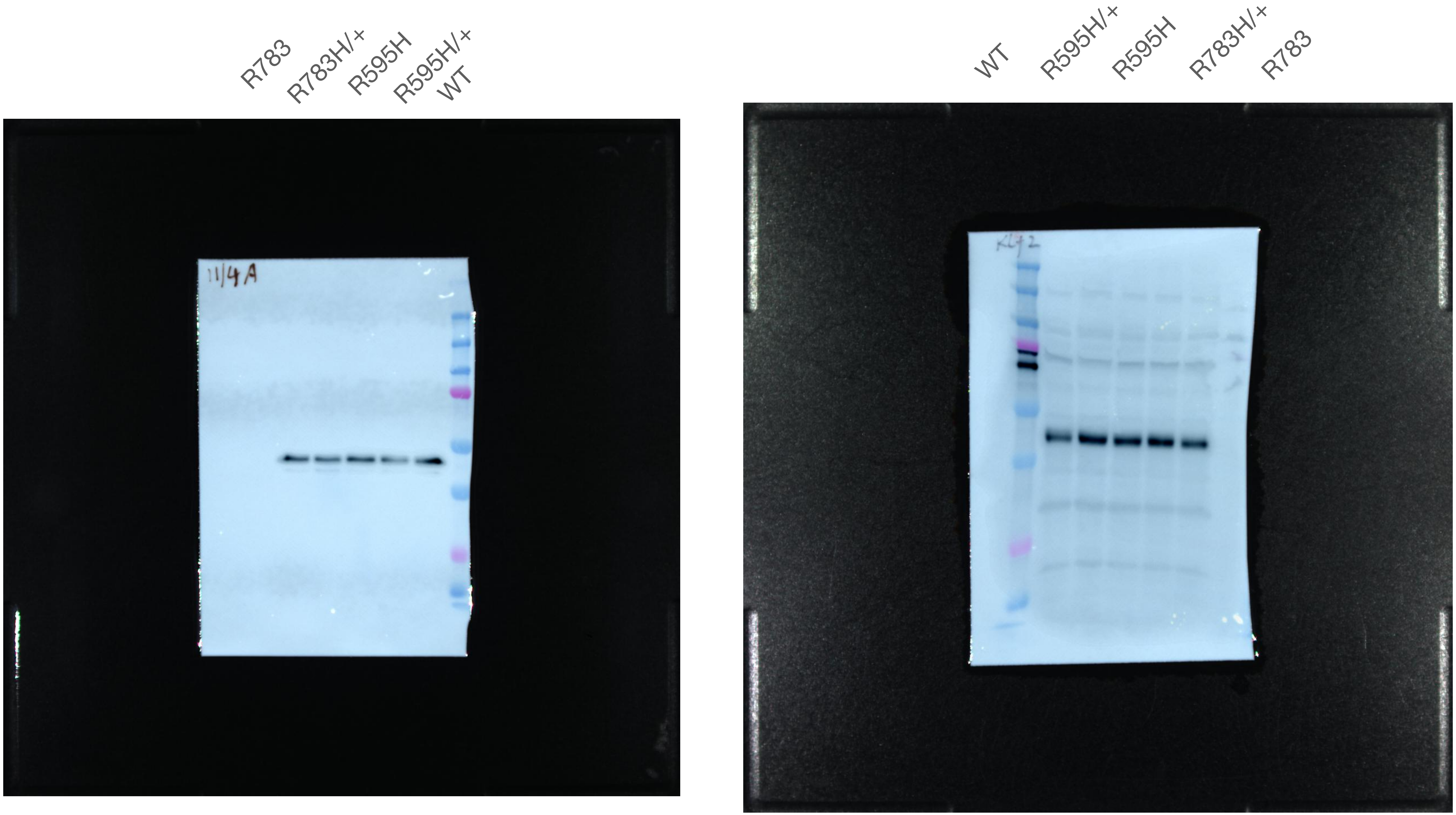

OCT4

KLF2

Figure 1A

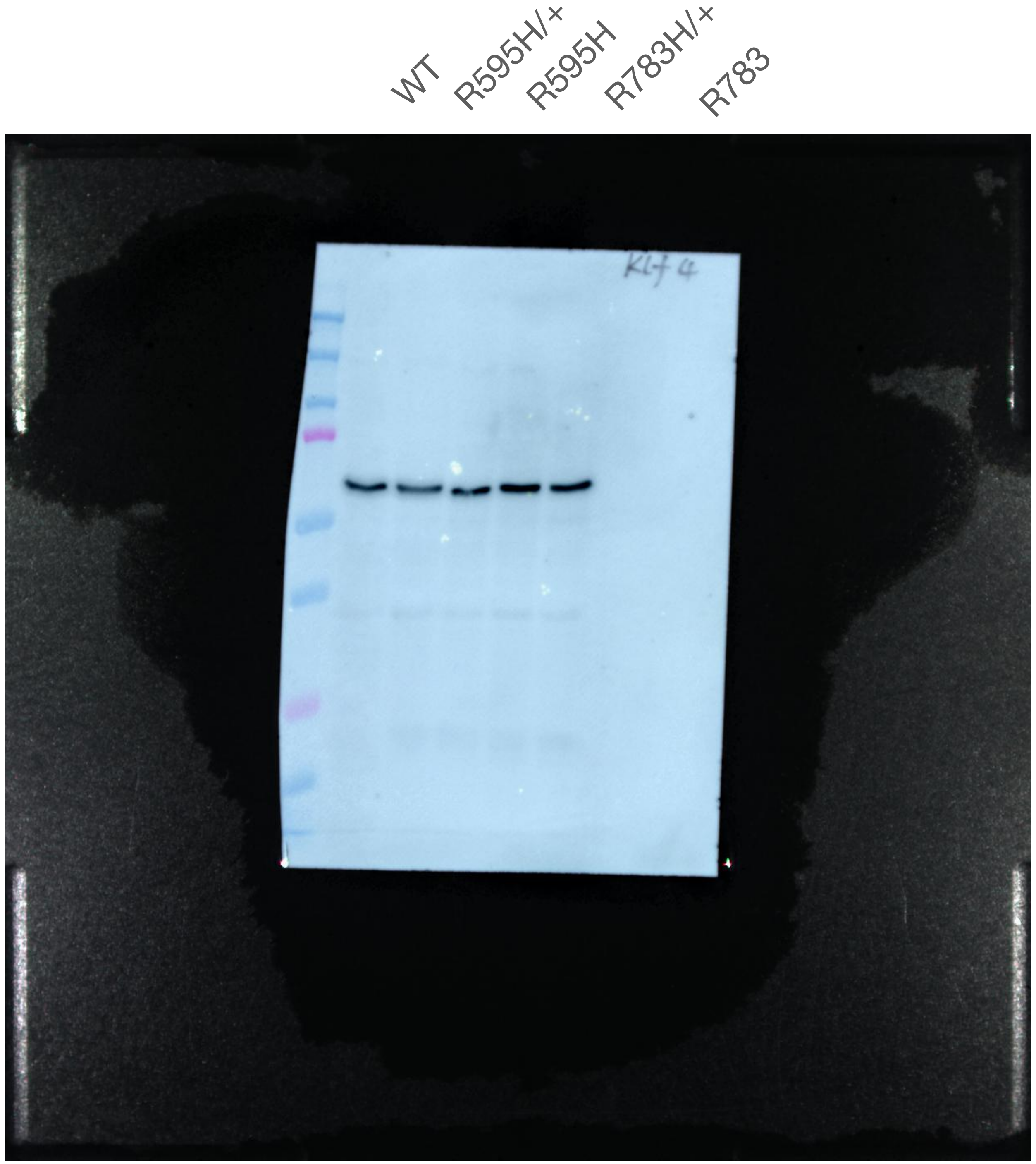

KLF4

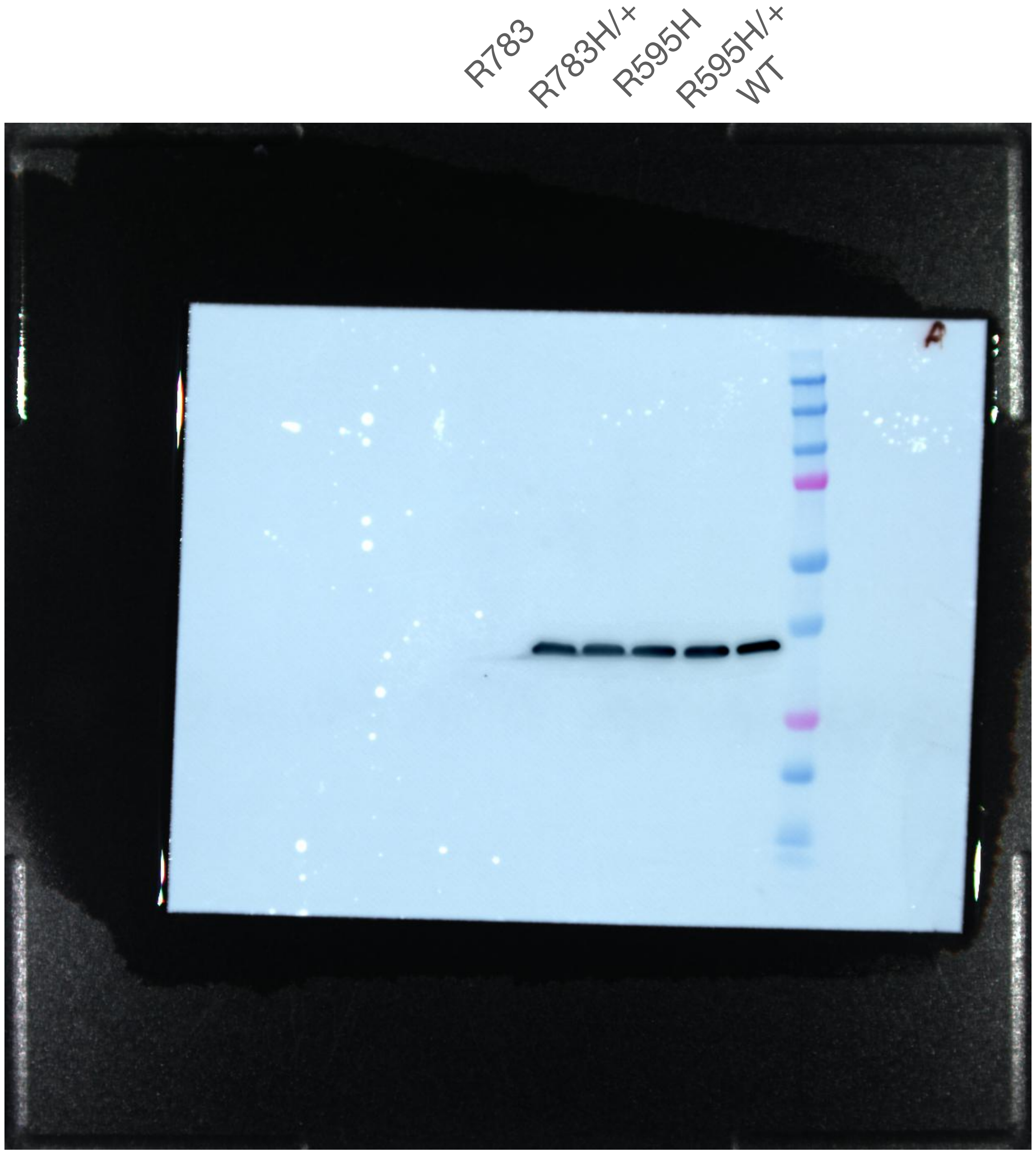

GAPDH

Figure 1C

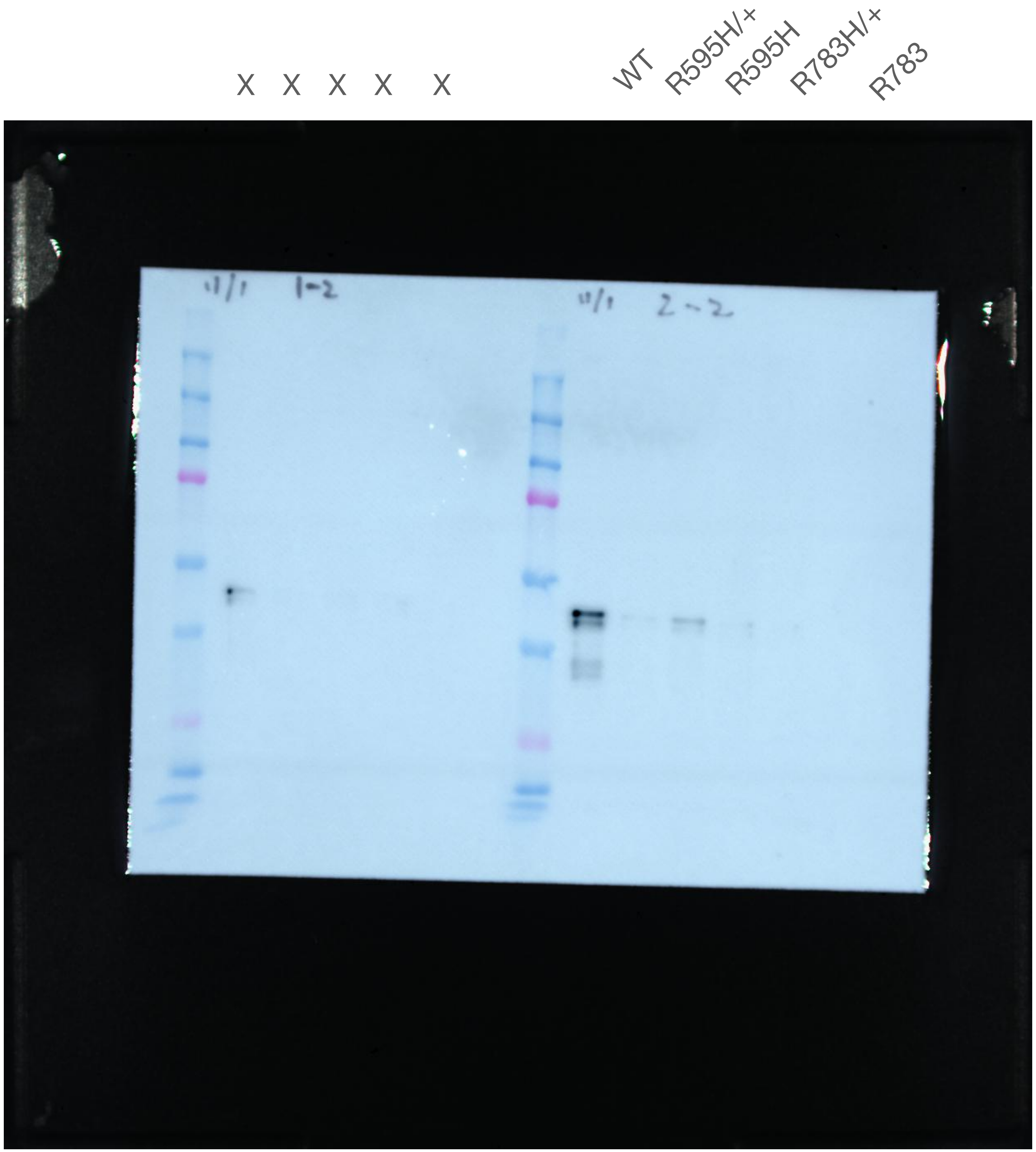

NANOG

Figure 1C

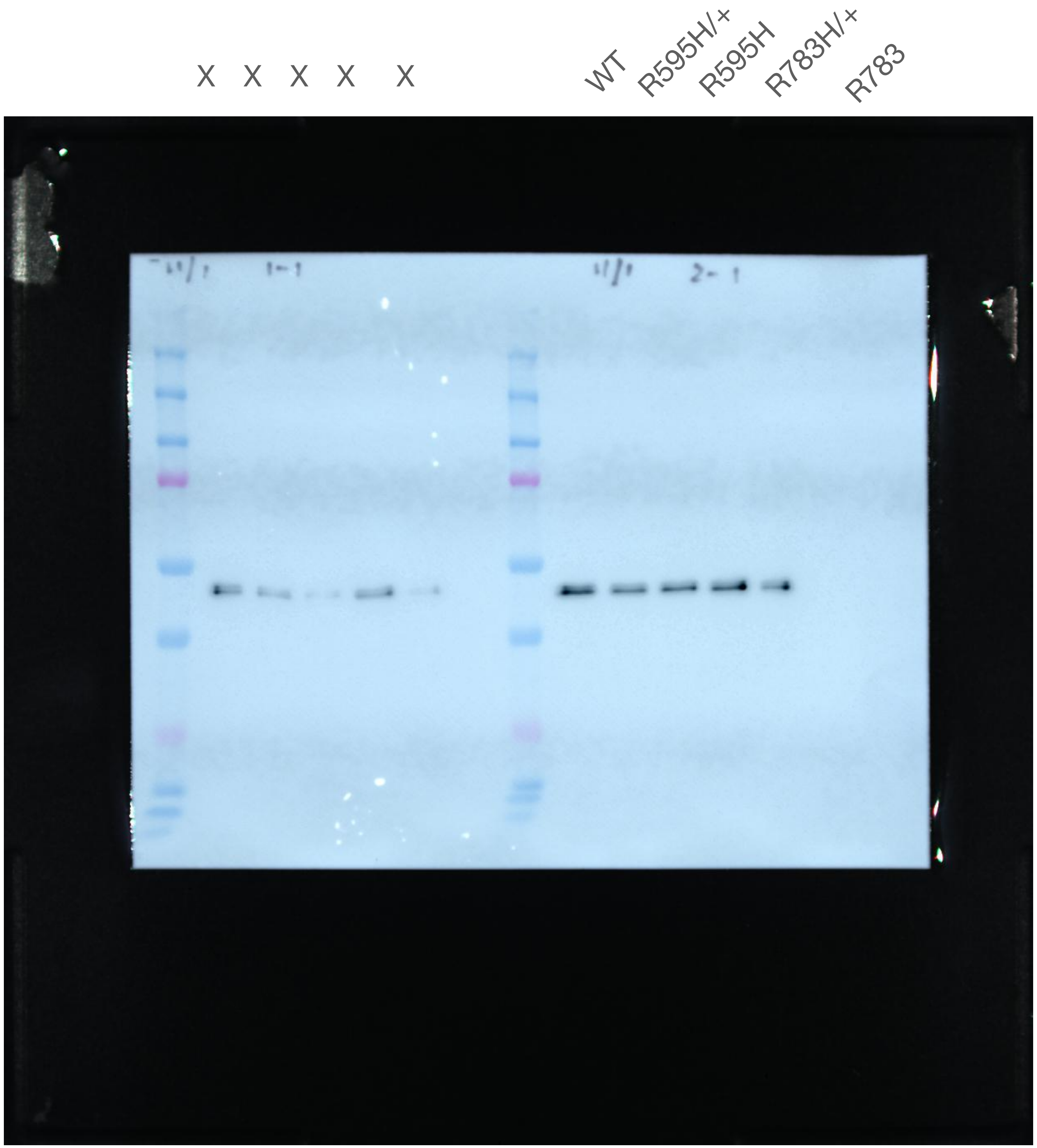

Oct4

Figure 1C

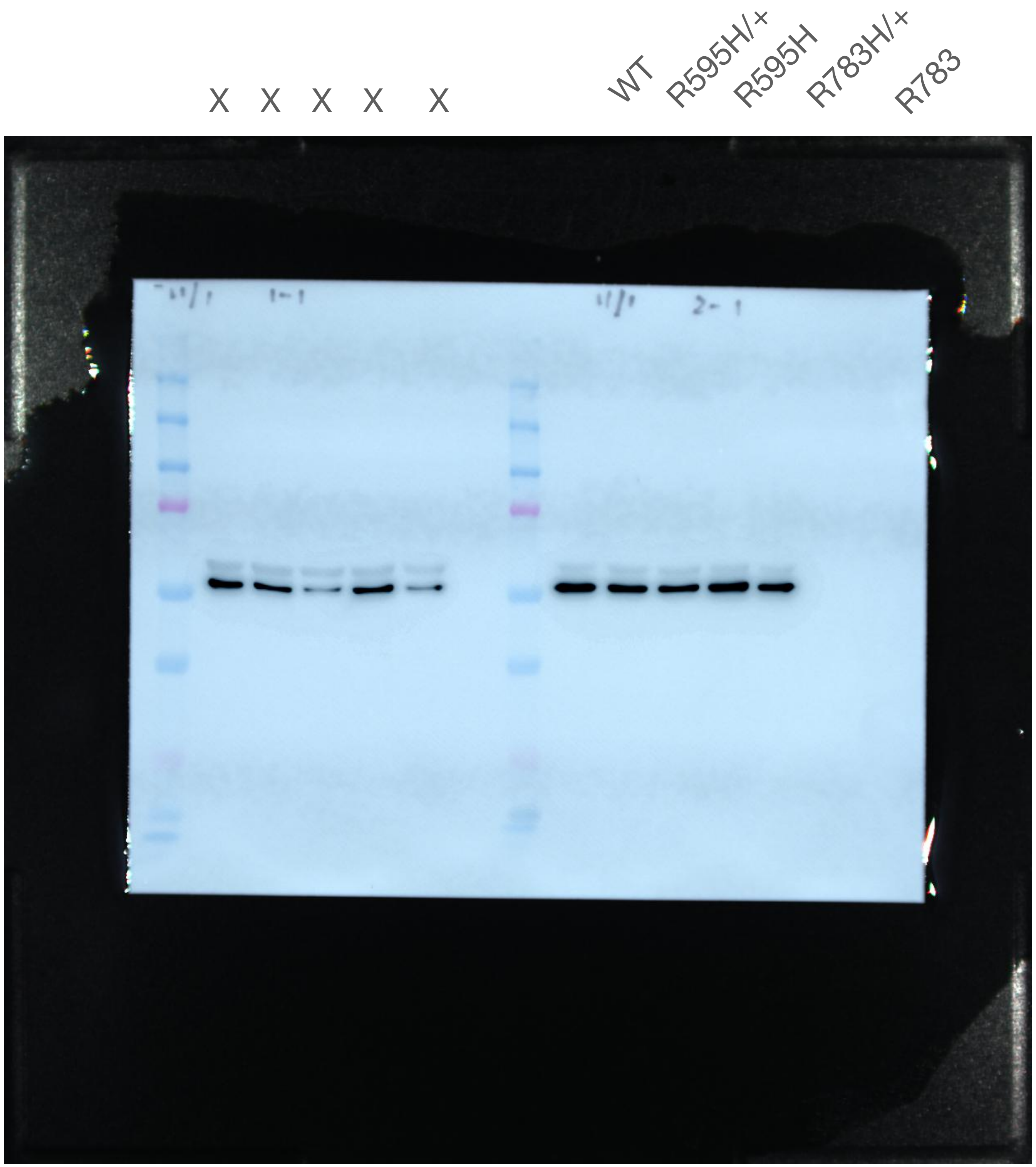

beta-TUBULIN

Figure 1F

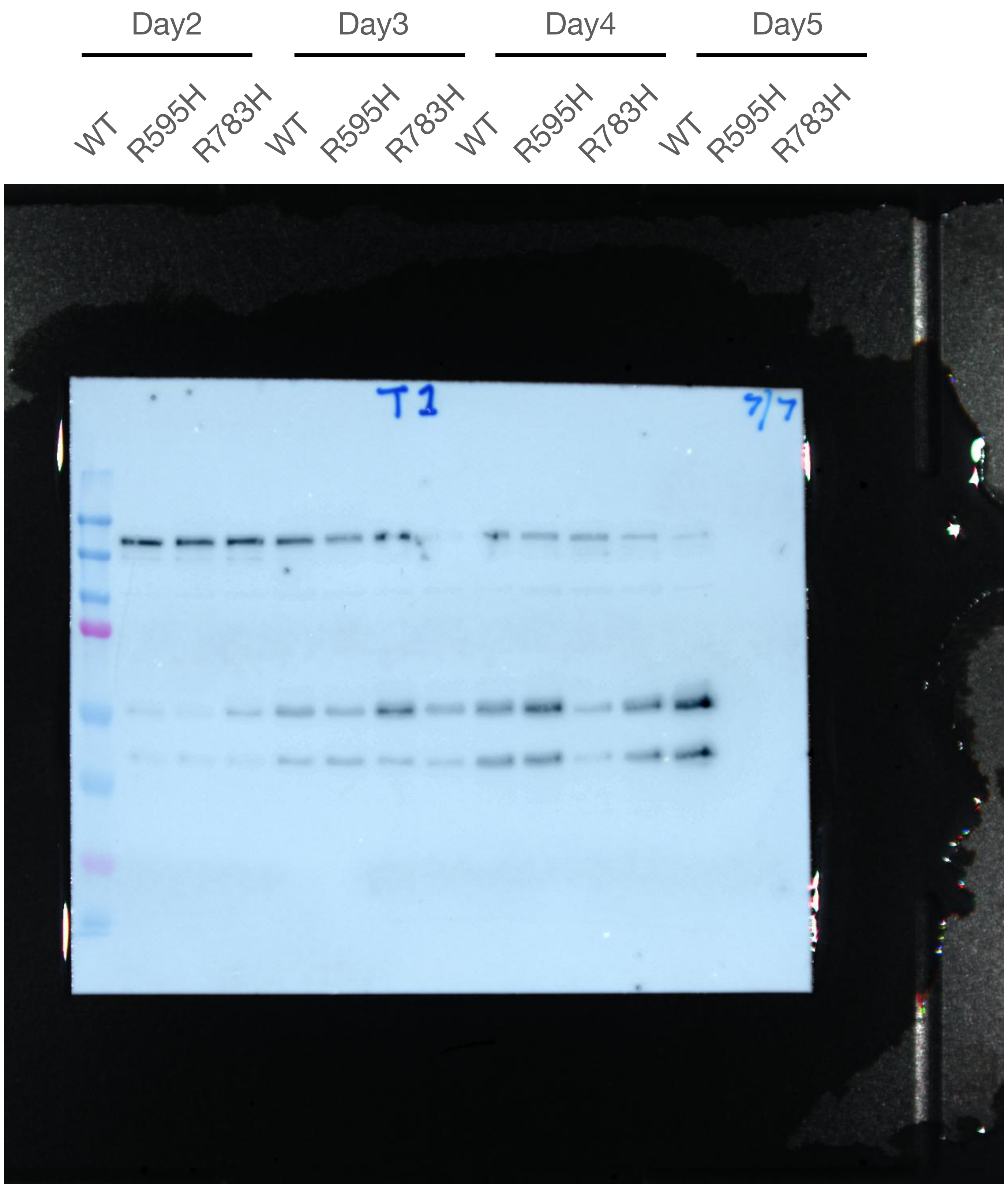

PAX6

Figure 1F

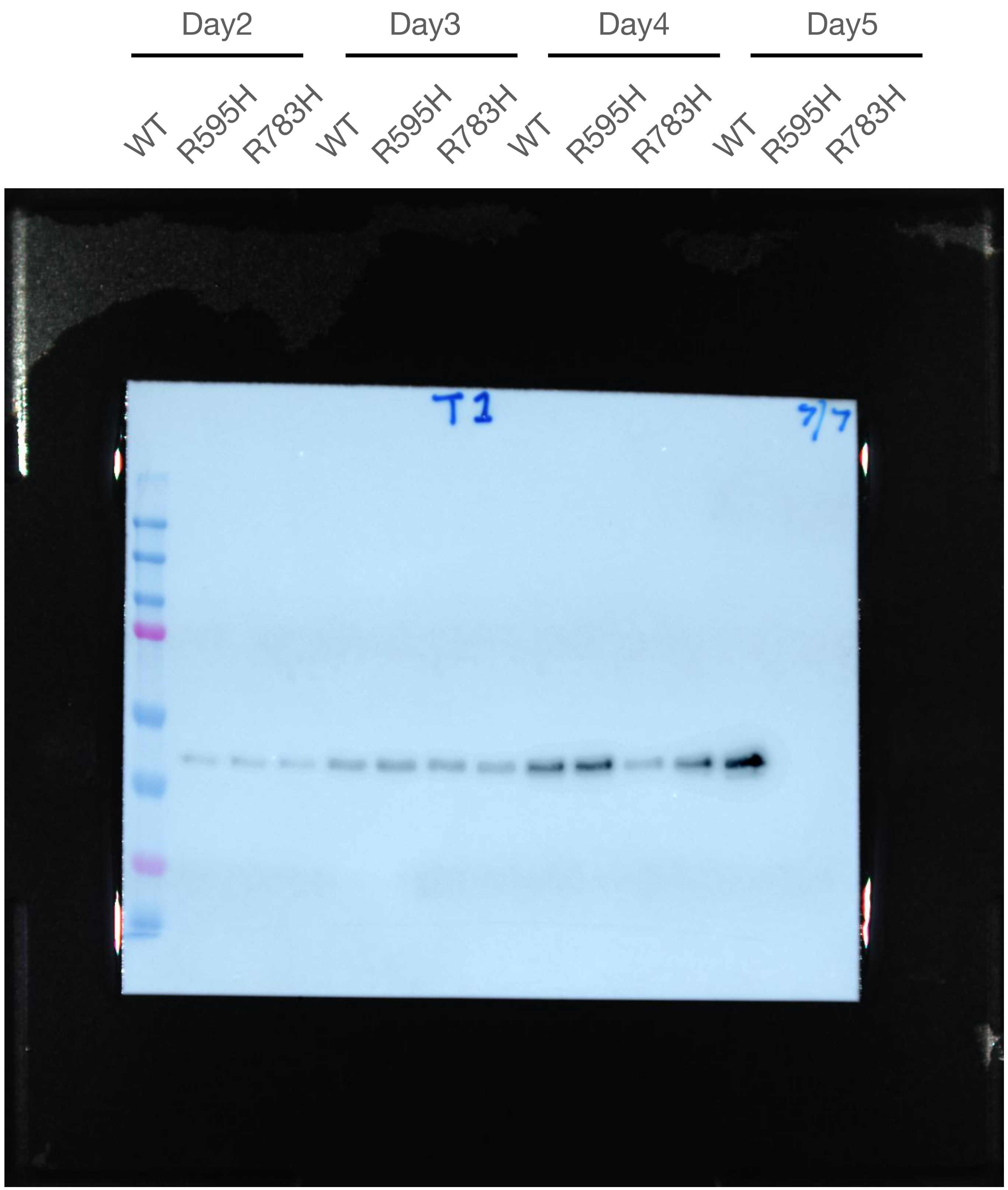

Sox1

Figure 1F

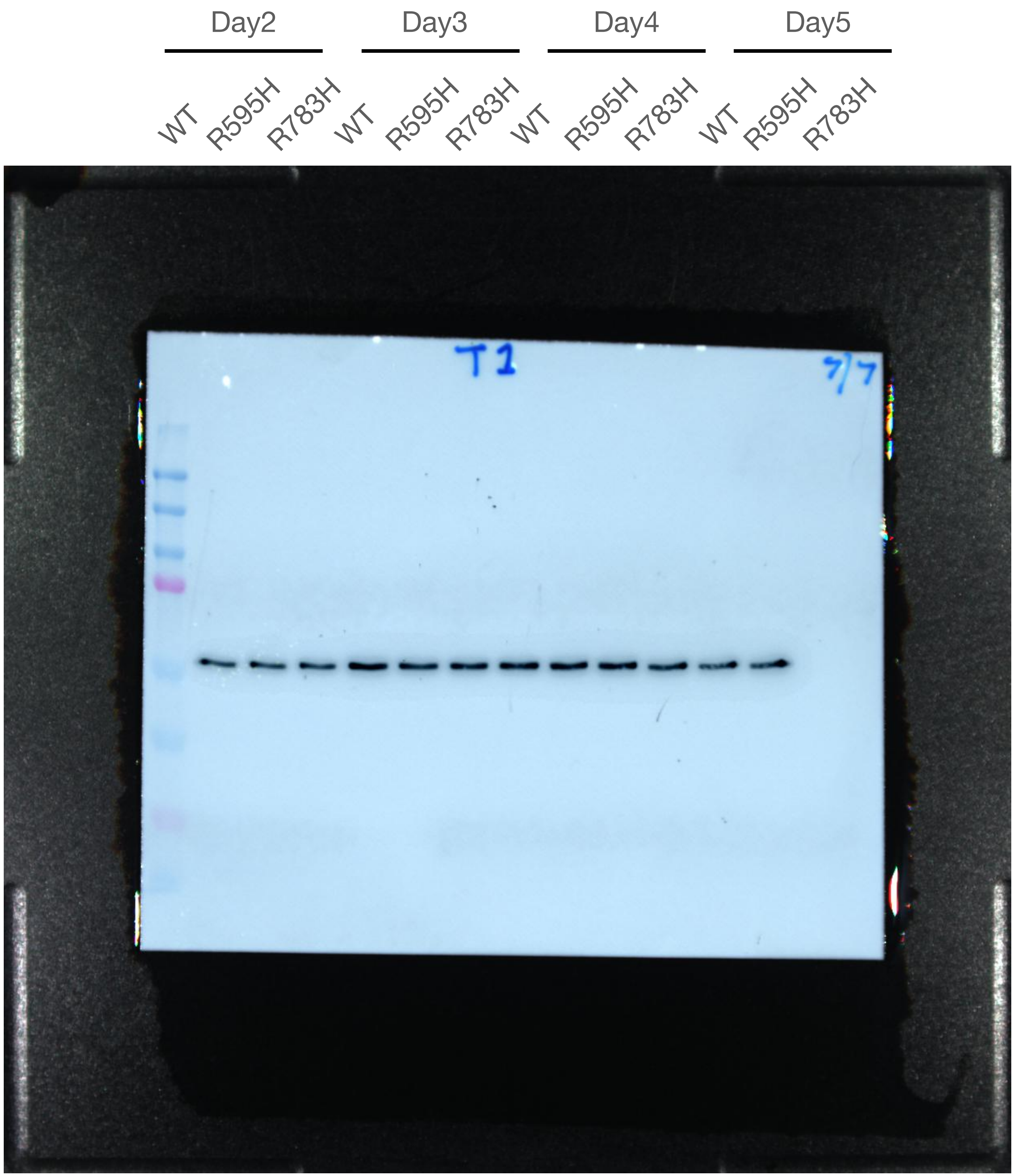

beta-TUBULIN

Figure 3C

Biological Replicate 1 | Biological Replicate 2

R783H R595H WT | R783H R595H WT

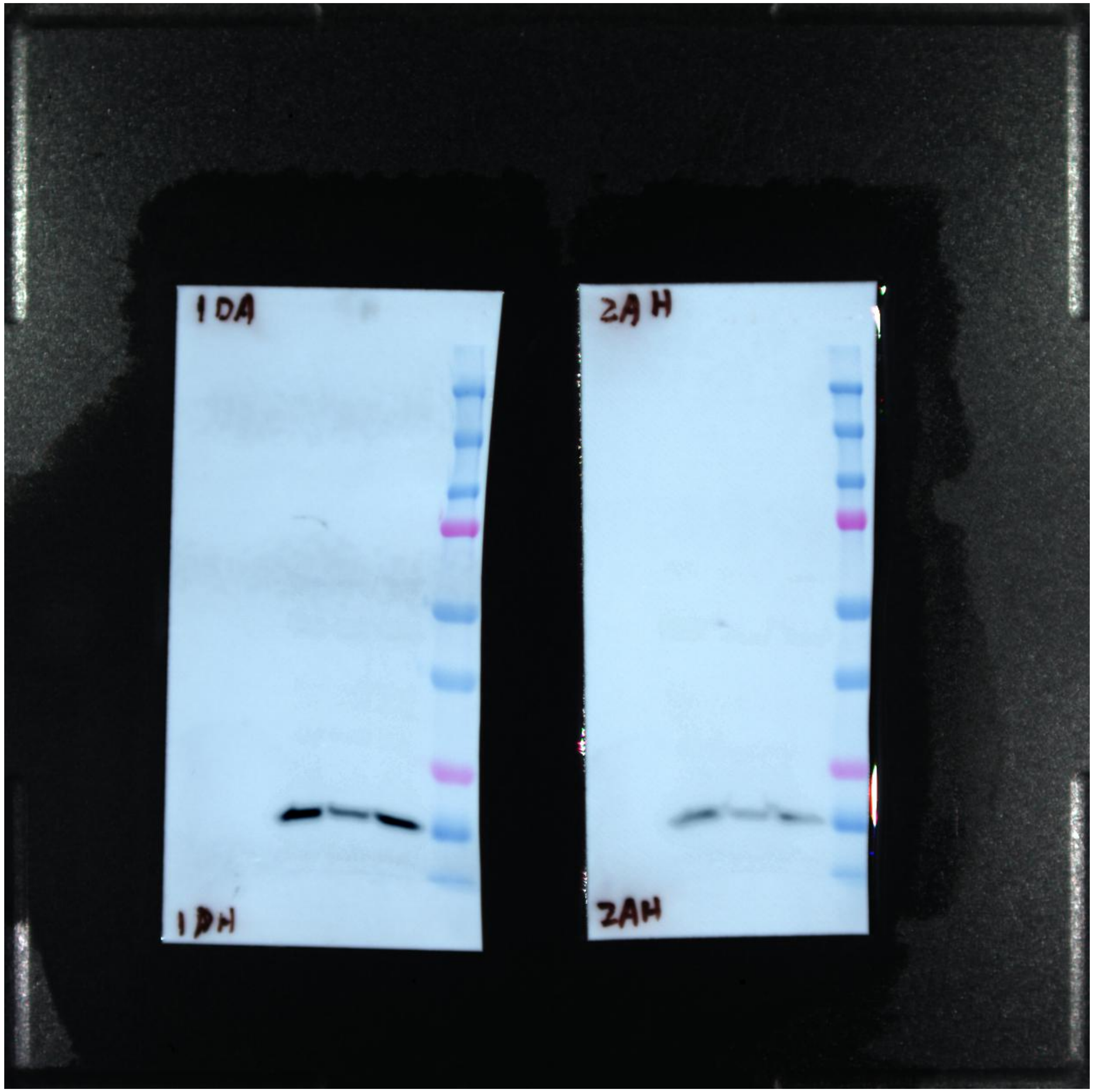

KRAS

Figure 3C

Biological Replicate 1 Biological Replicate 2

R783H R595H WT R783H R595H WT

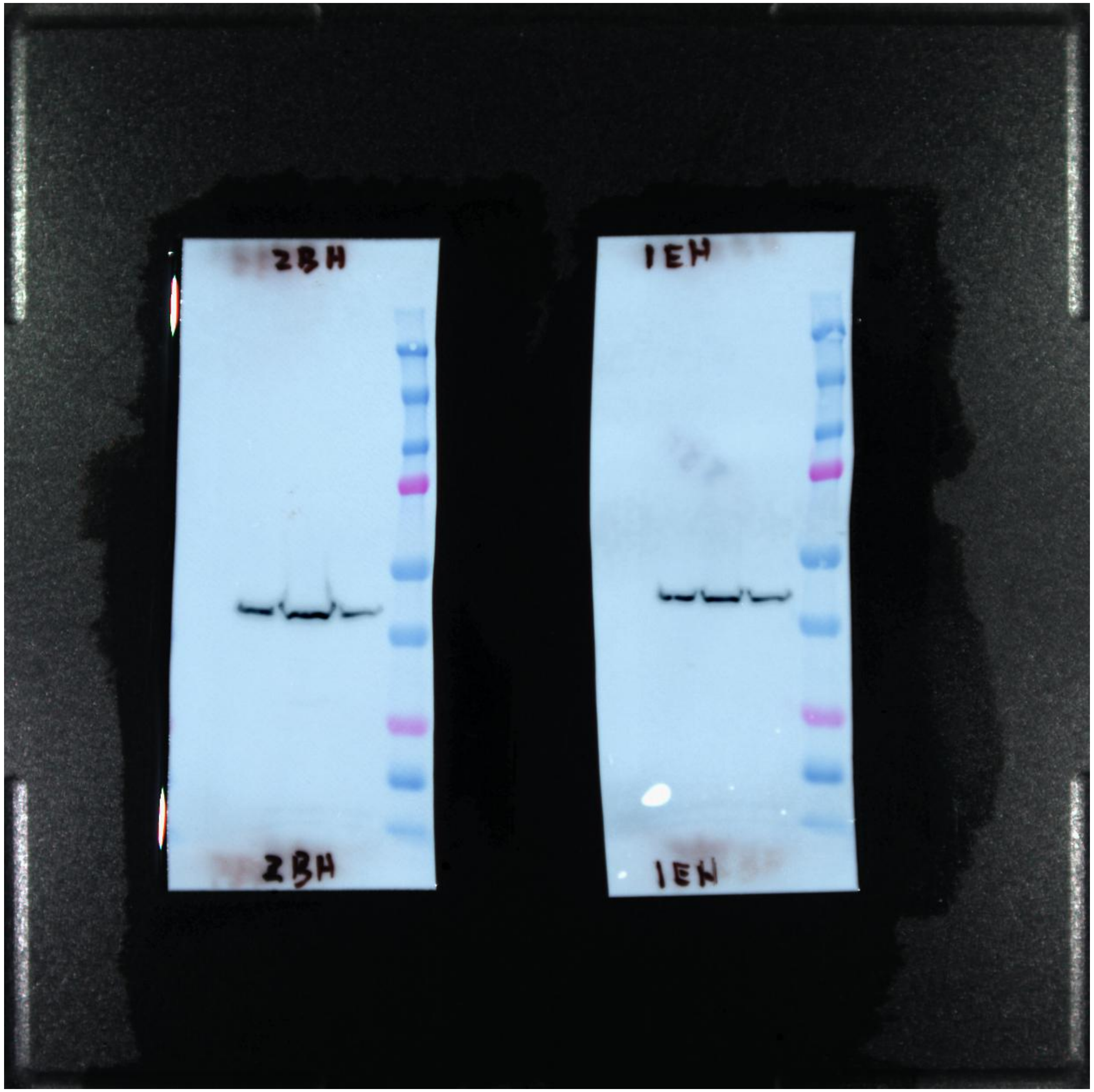

TDP43

Figure 3C

Biological Replicate 1 Biological Replicate 2

R783H R595H WT R783H R595H WT

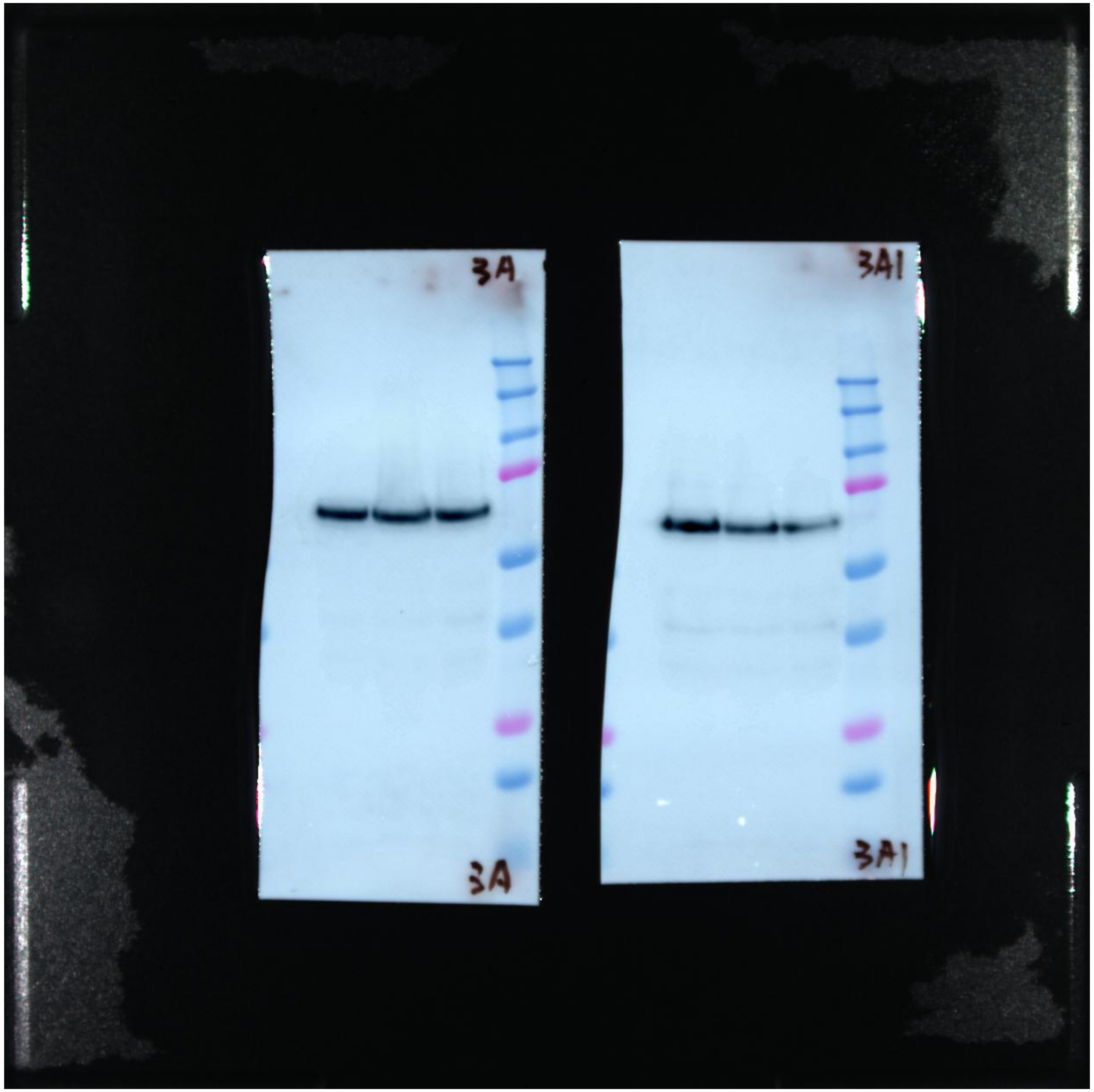

NMYC

Figure 3C

Biological Replicate 1 Biological Replicate 2

R783H R595H WT R783H R595H WT

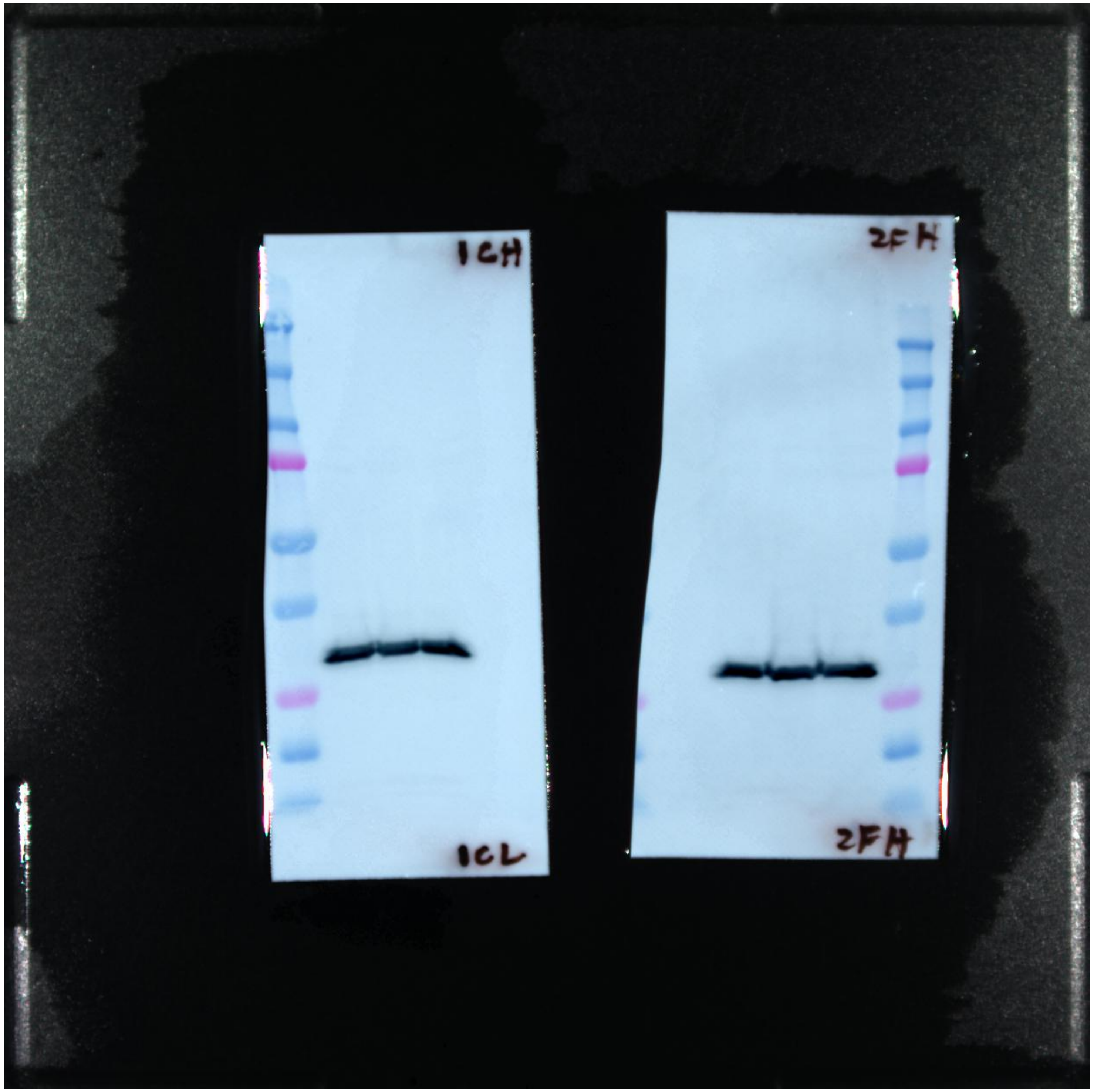

CDC2

Figure 3C

Biological Replicate 2 Biological Replicate 1

R783H R595H WT R783H R595H WT

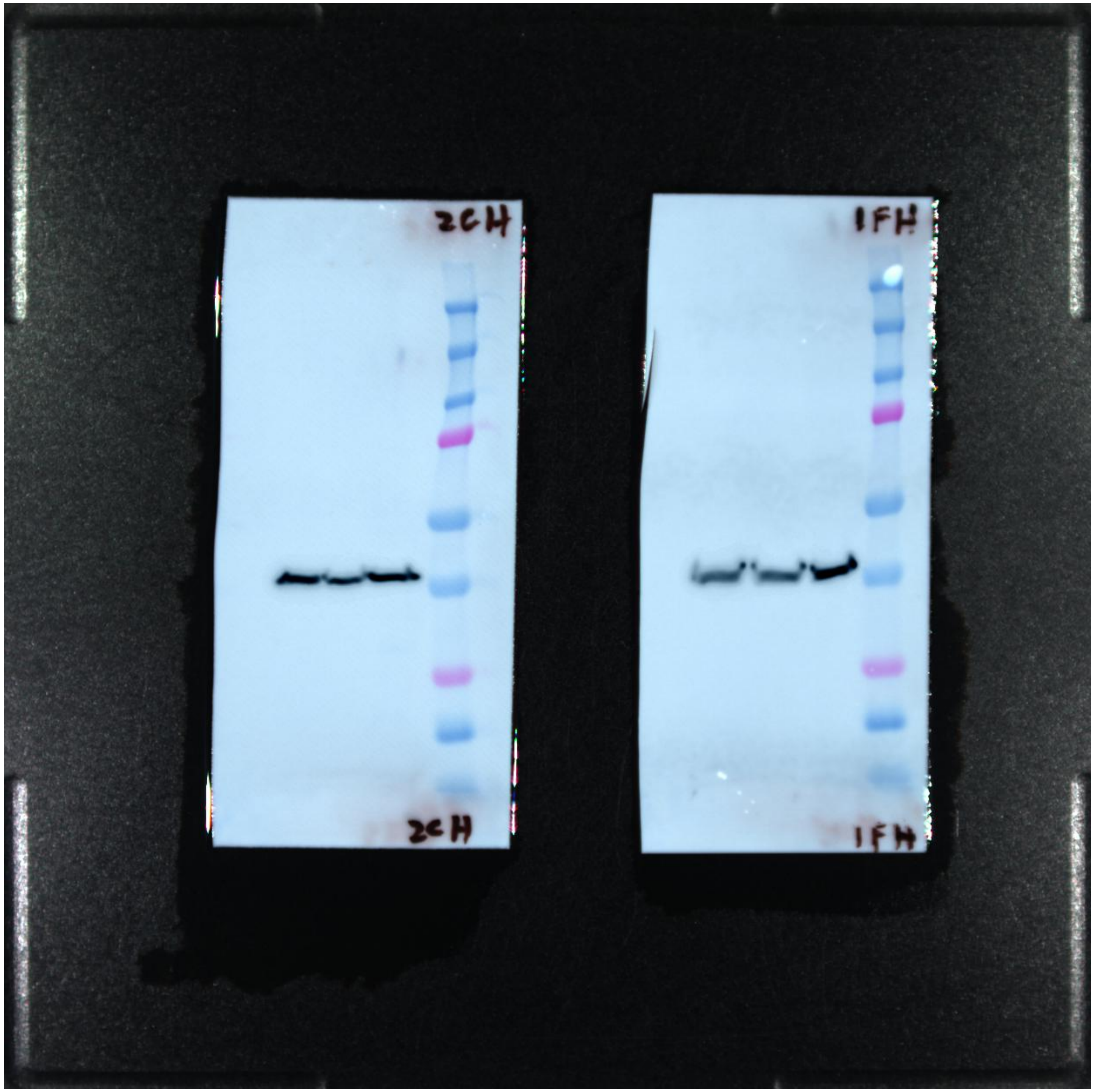

hnRNP-E1

Figure 3C

Biological Replicate 1 Biological Replicate 2

WT R595H R783H WT R595H R783H

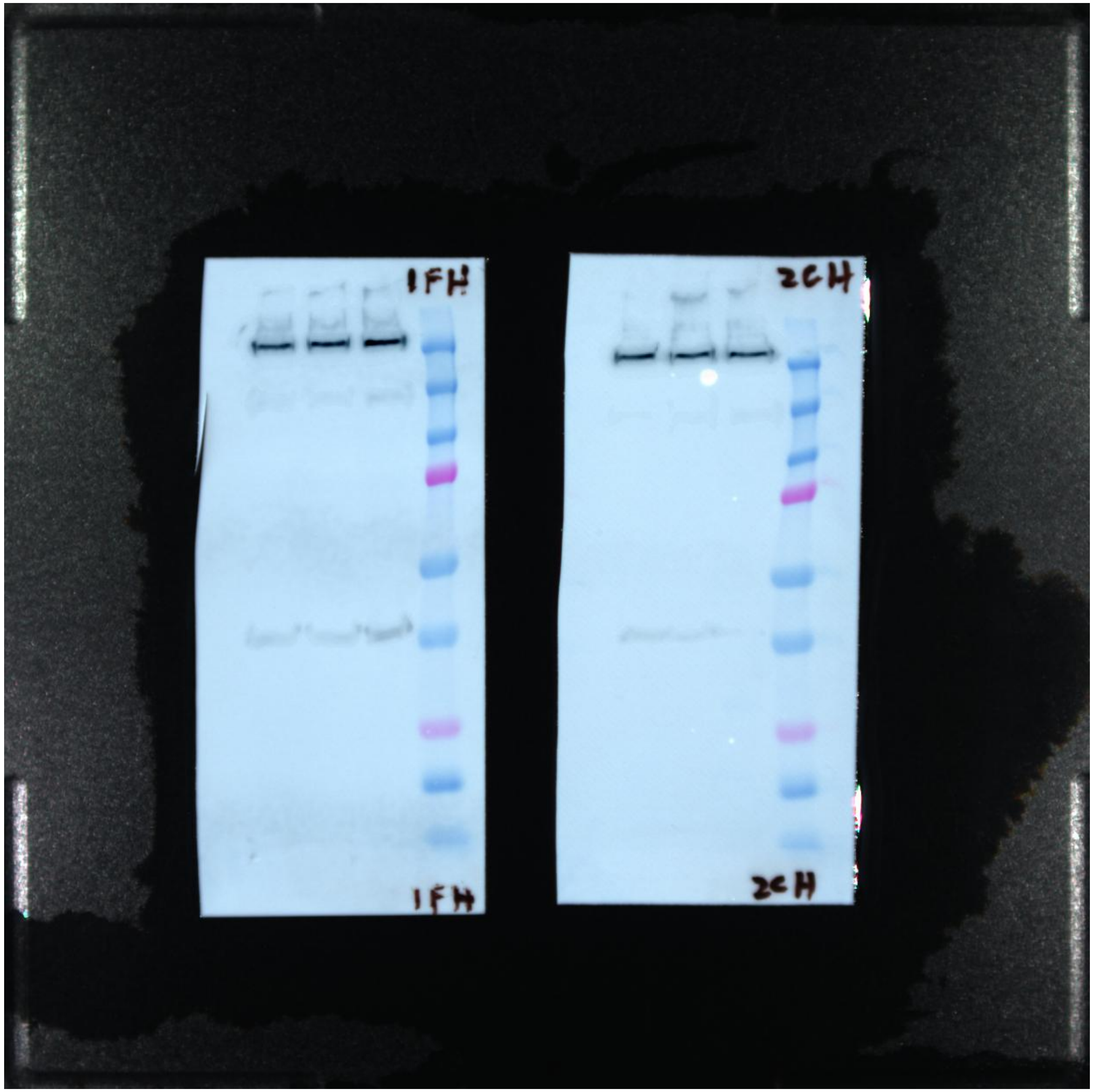

MTOR

Figure 3C

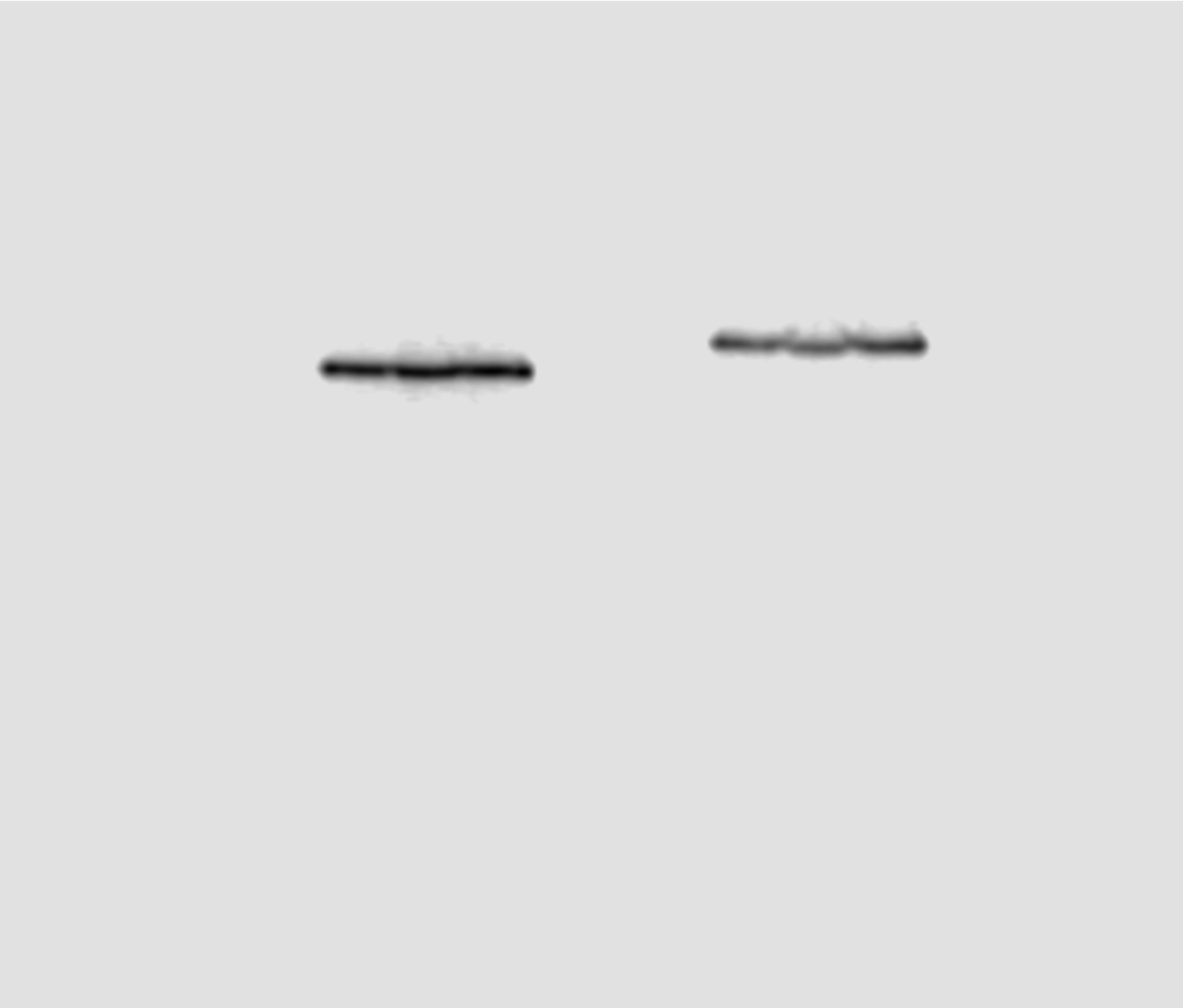

RAGC

Note: these two blots were scanned on a Li-Cor C-Digit Blot Scanner

Figure 3C

Biological Replicate 1 | Biological Replicate 2

WT R595H R783H | R783H R595H WT

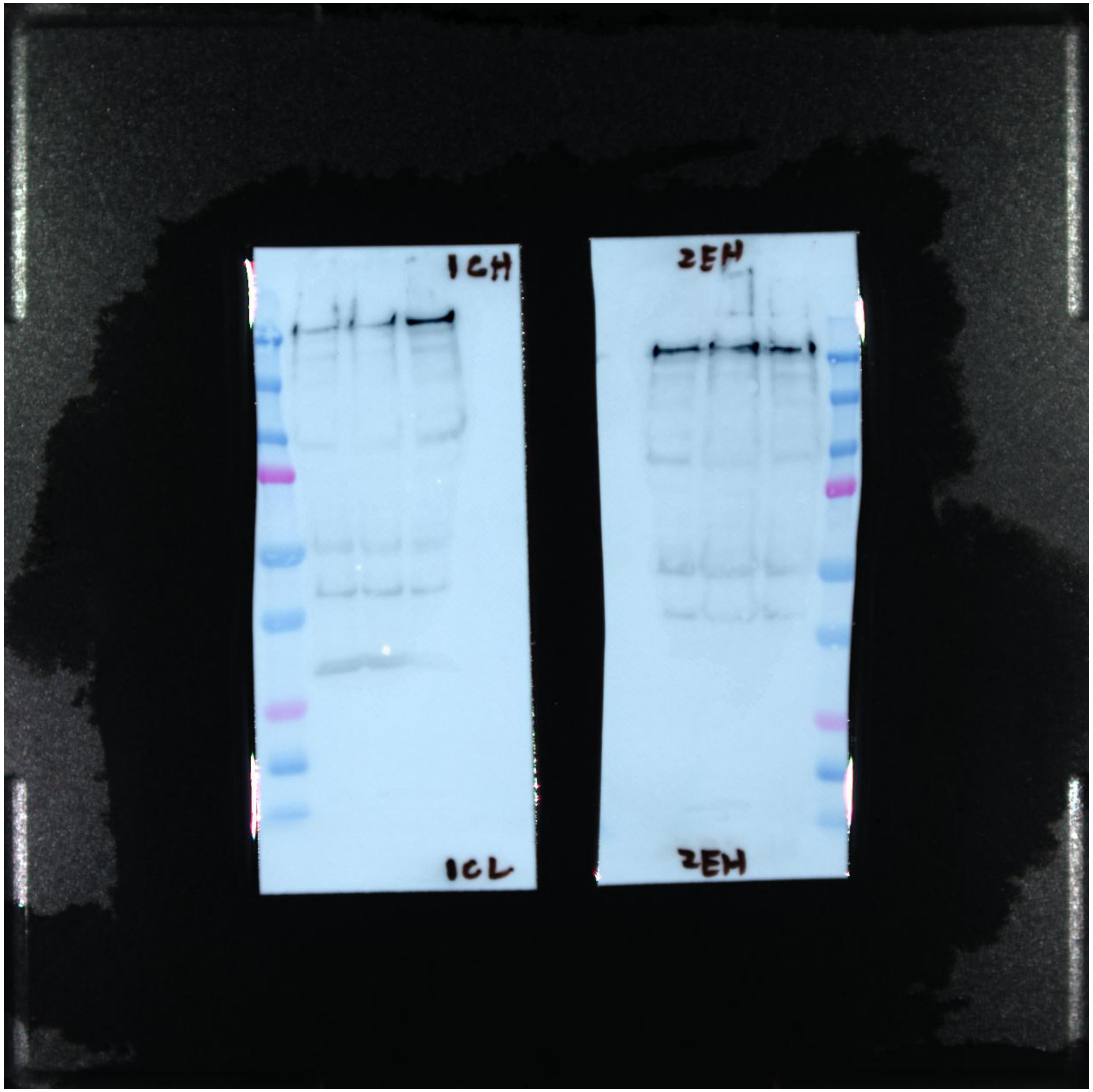

TET2

Figure 3C

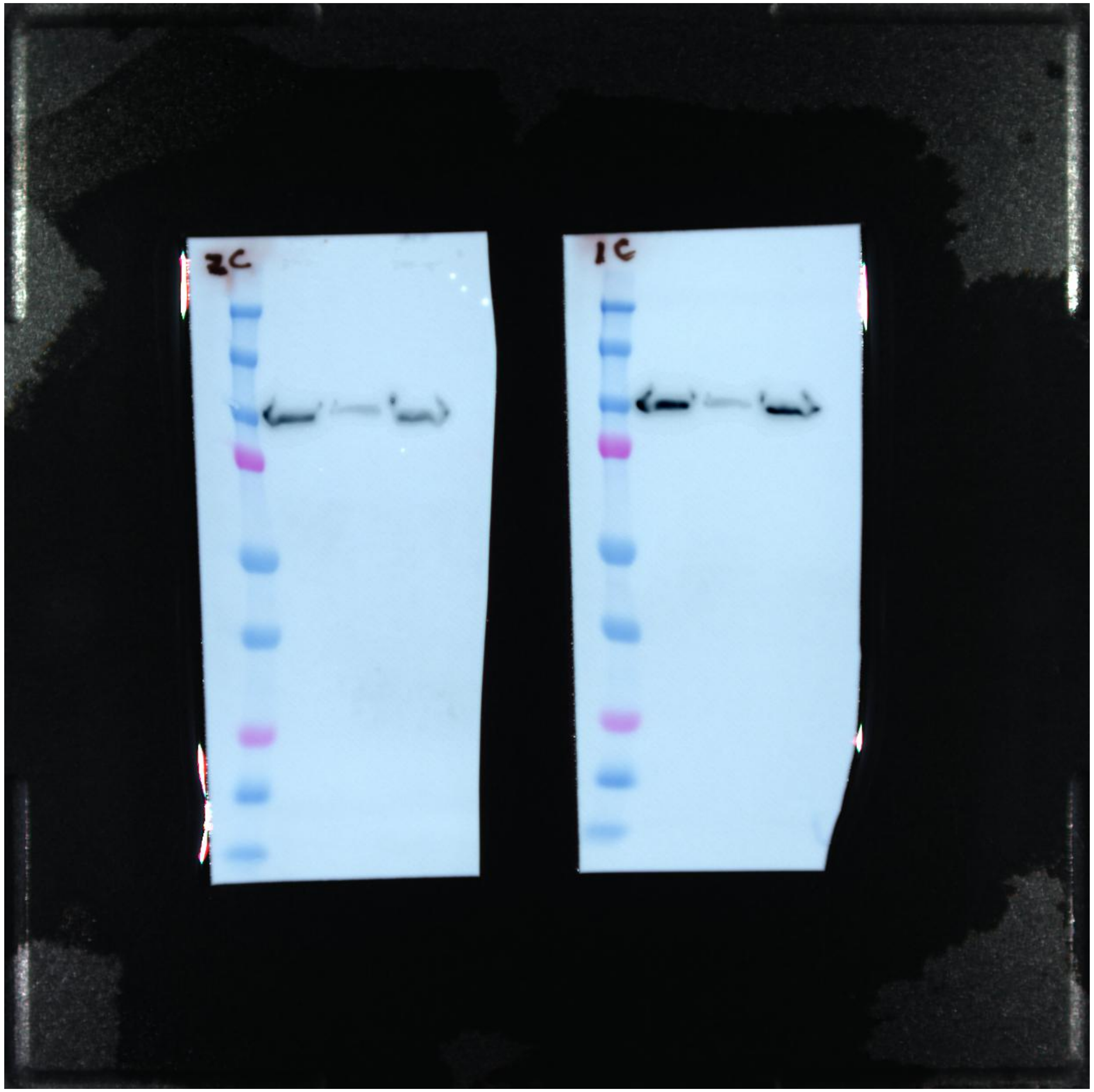

CTNNB1

Figure 3C

Biological Replicate 1 | Biological Replicate 2

WT R595H R783H | WT R595H R783H

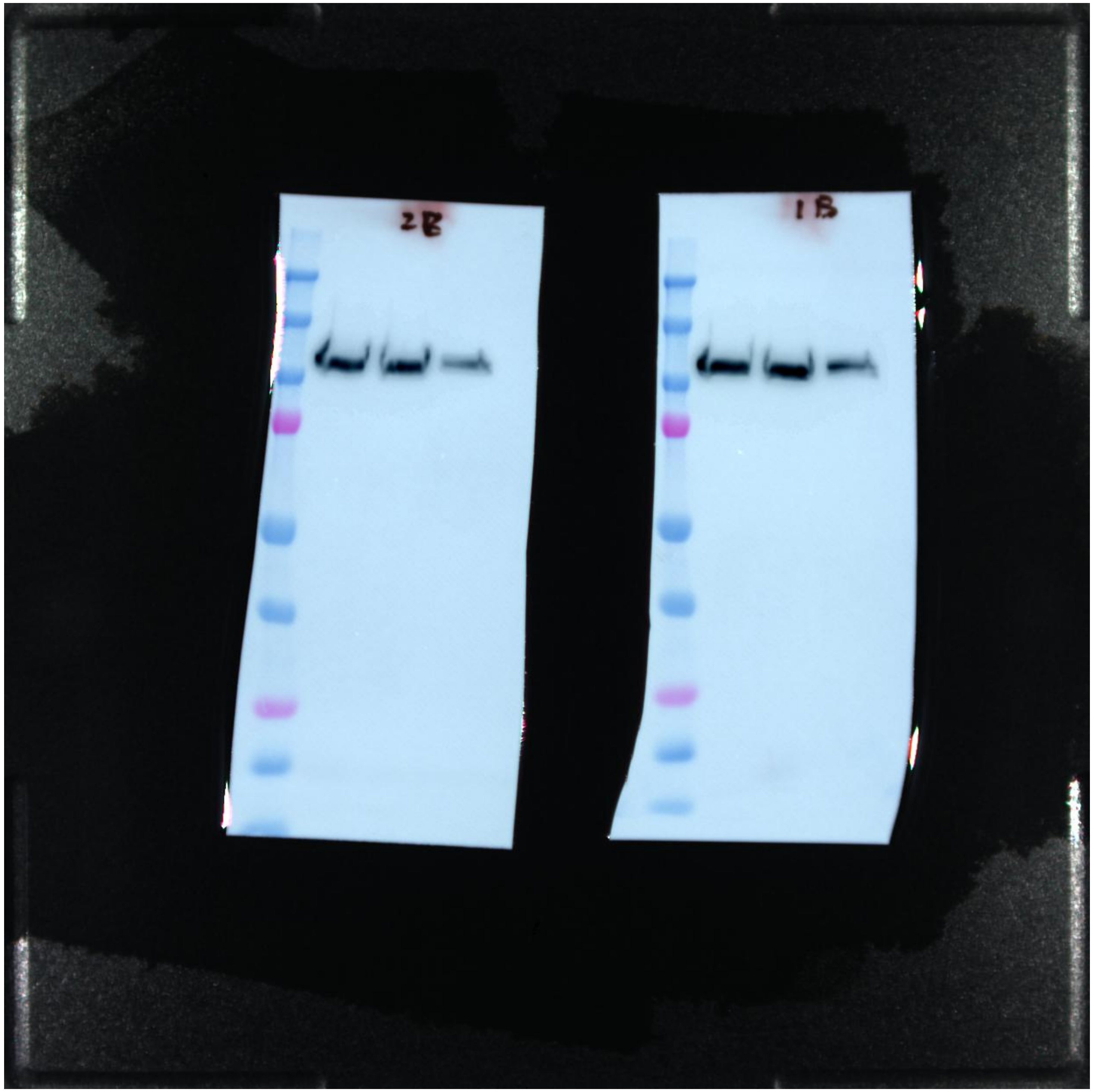

LSD1

Figure 3C

Biological Replicate 2 | Biological Replicate 1

R783H R595H WT | R783H R595H WT

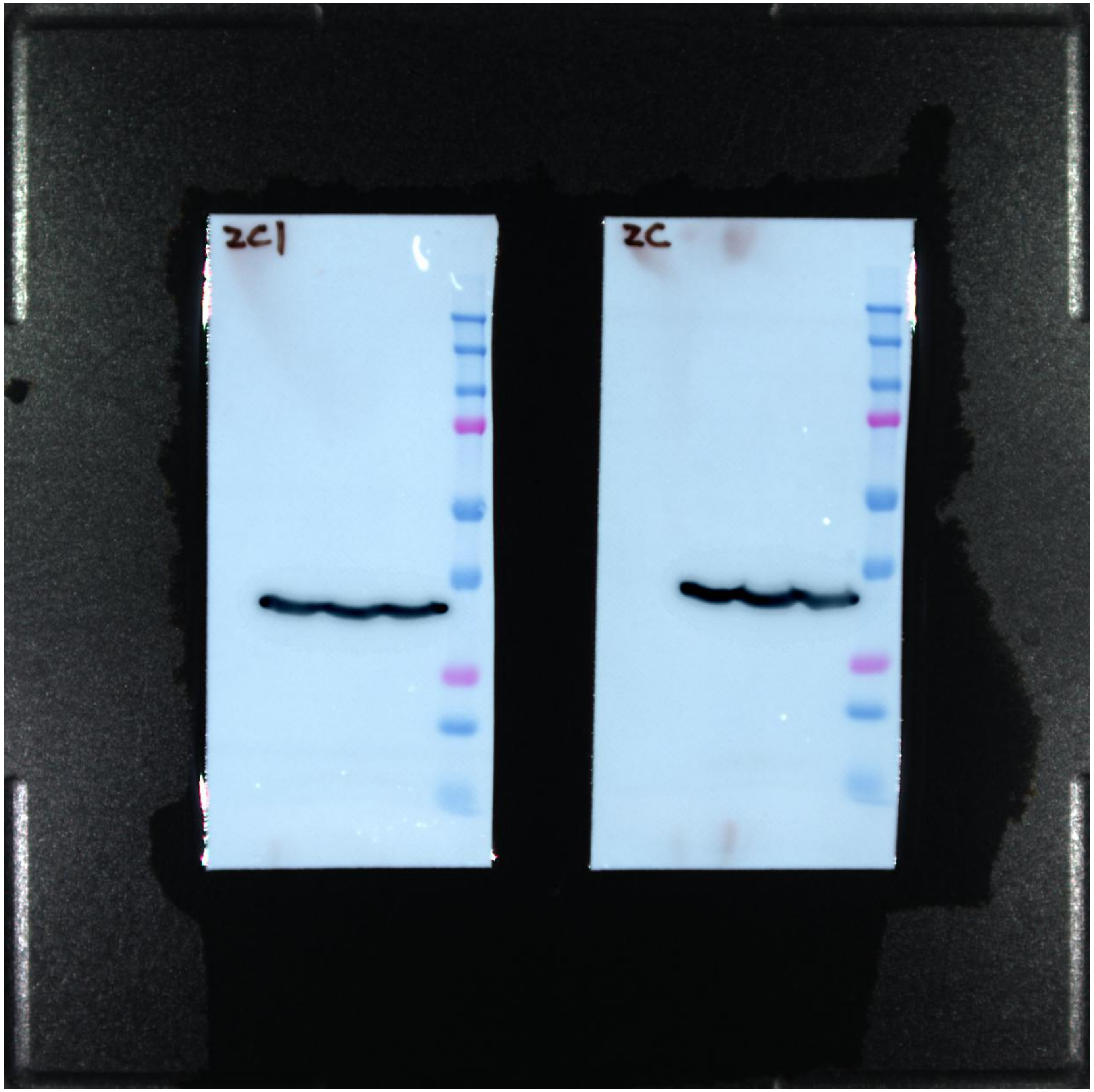

GAPDH

Figure 4A

R783H R595H WT

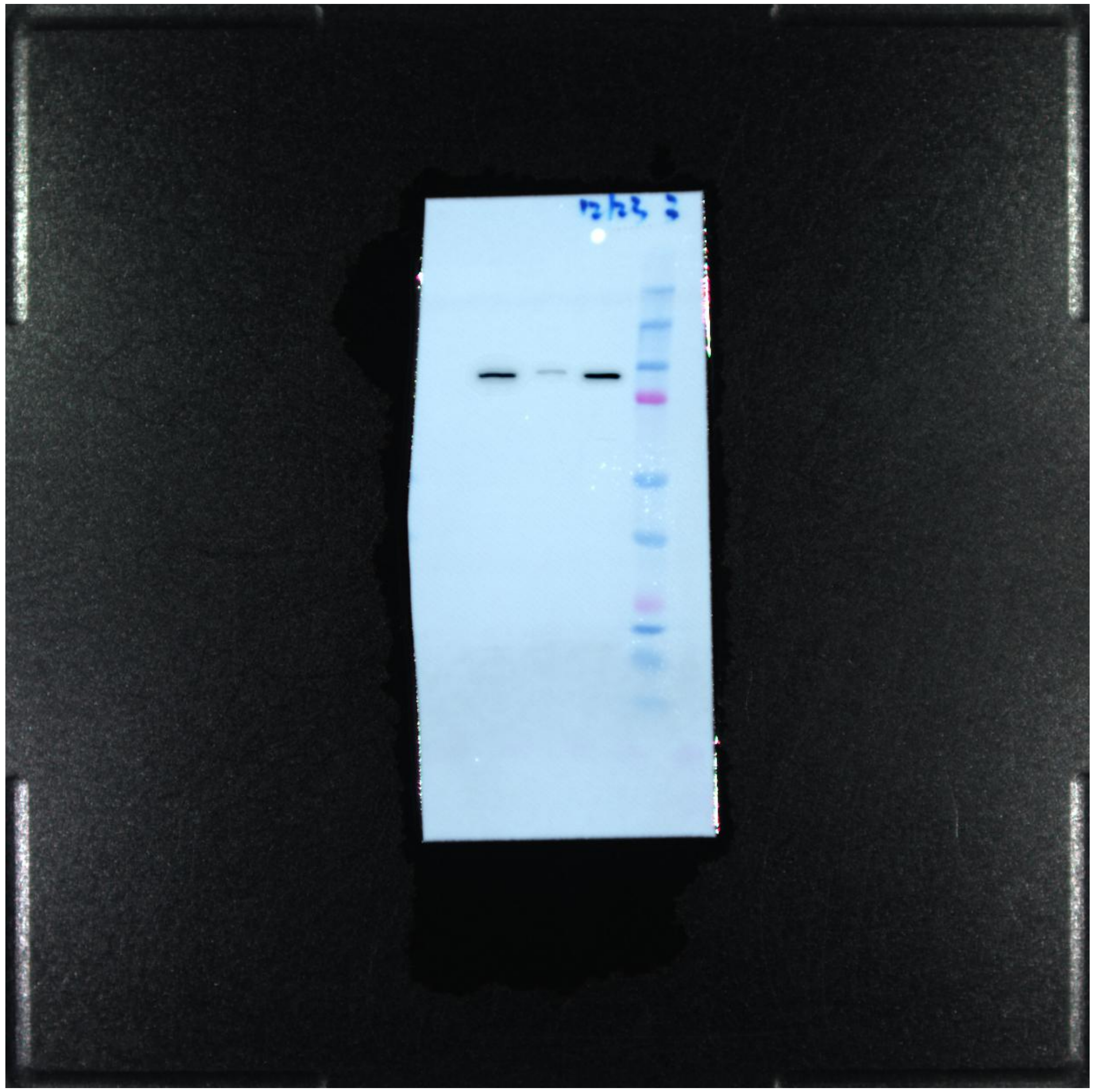

CTNNB1

R783H R595H WT

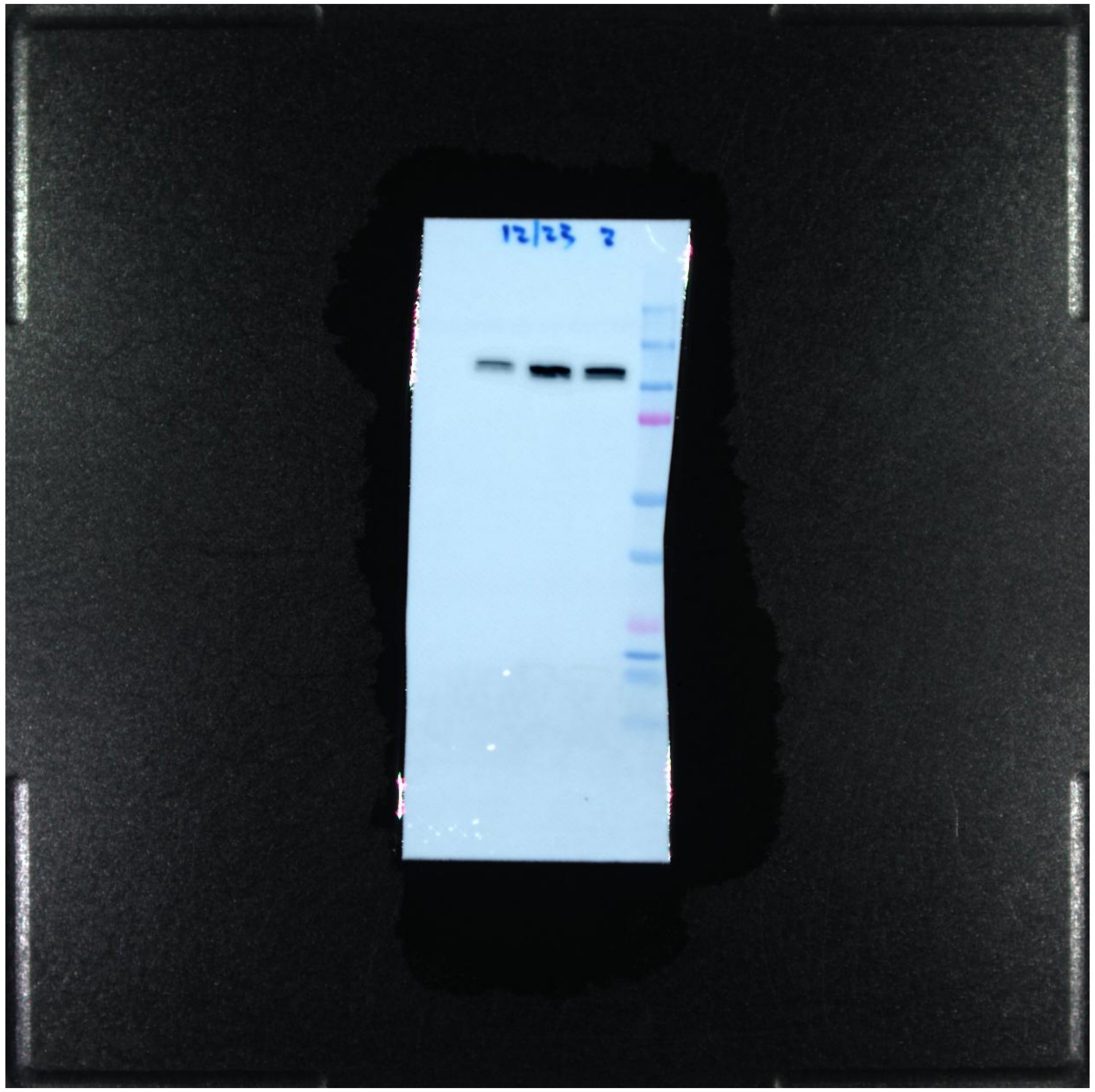

LSD1

Figure 4A

R783H R595H WT

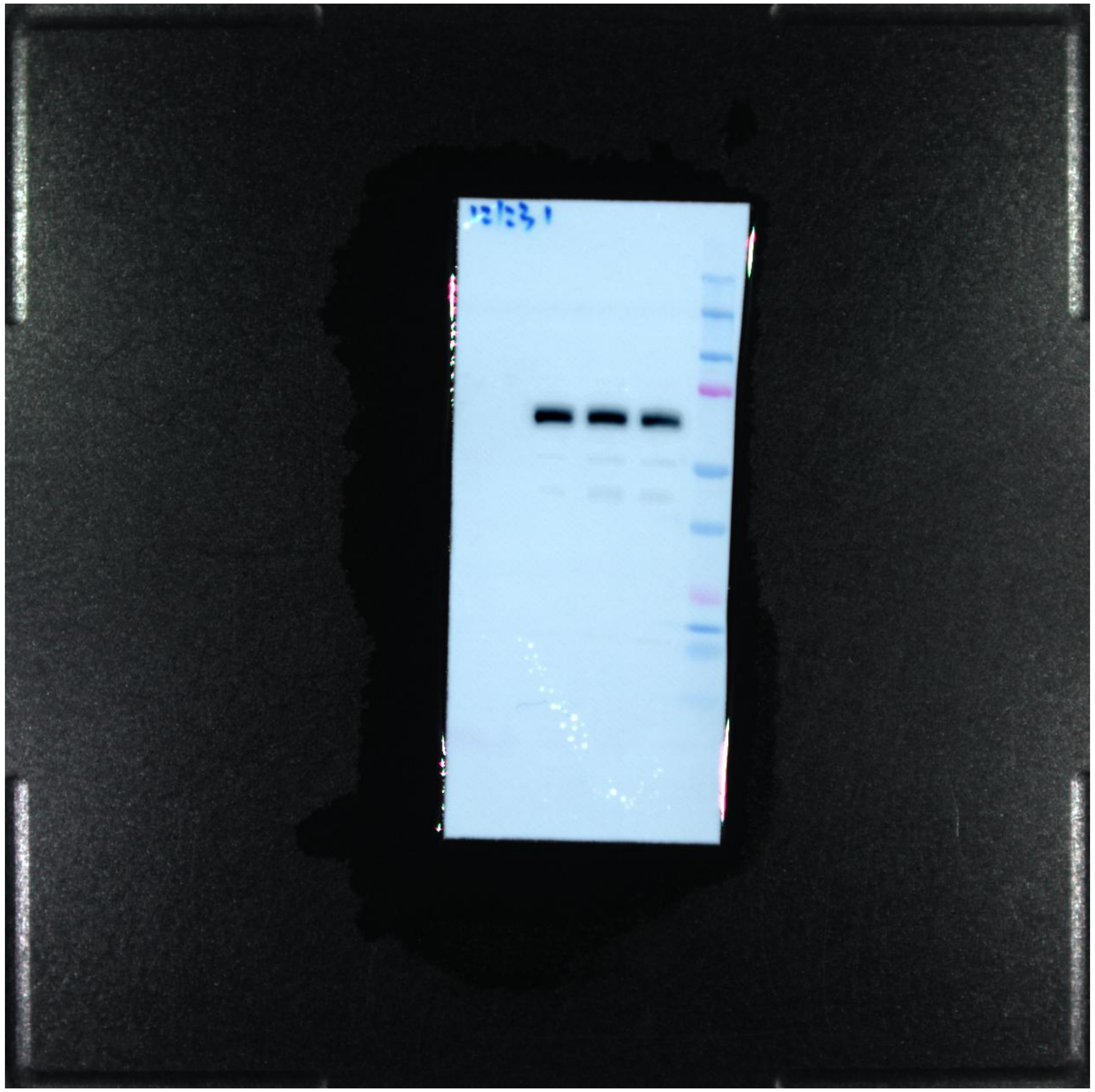

NMYC

R783H R595H WT

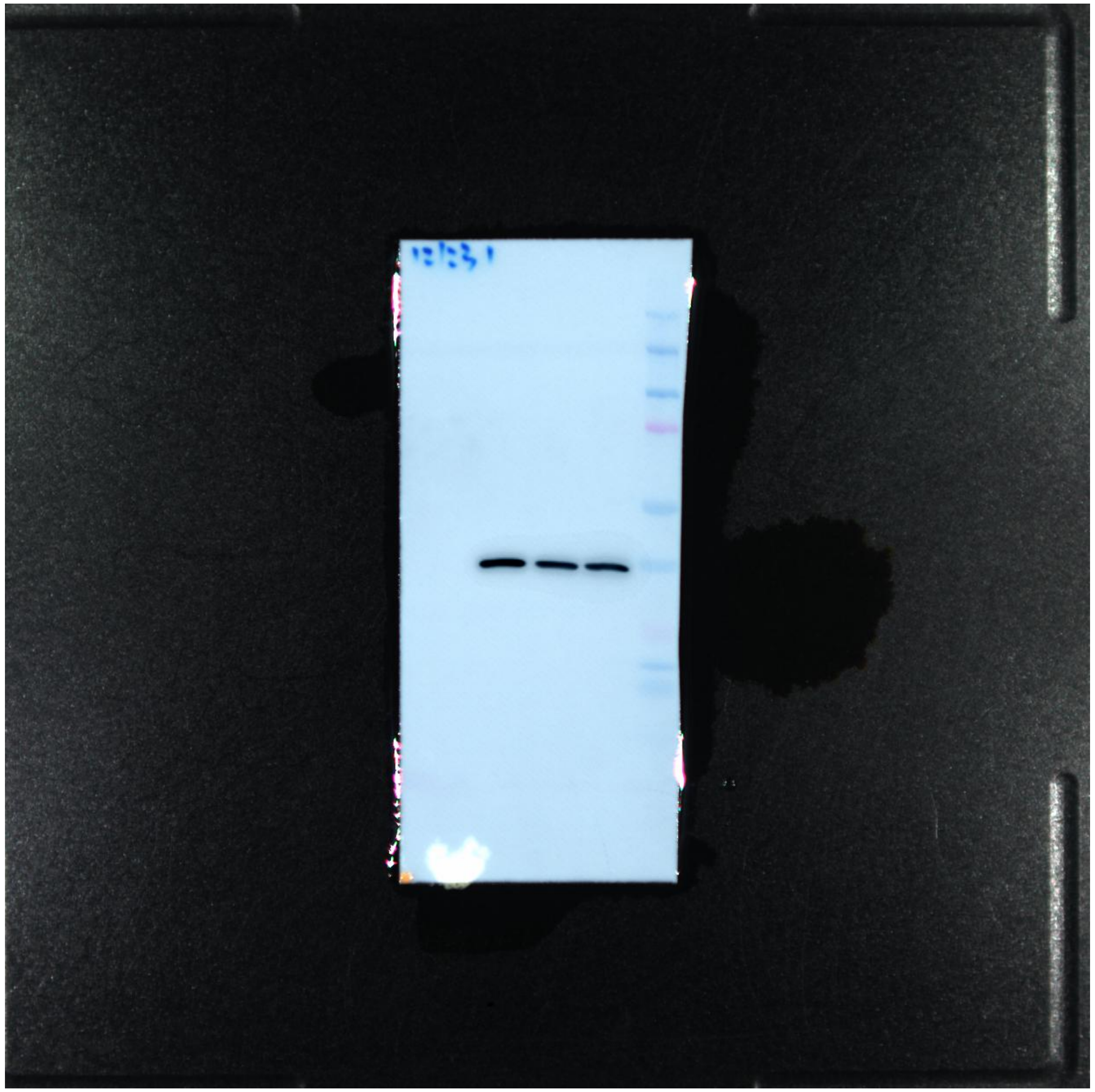

GAPDH

Figure 4H

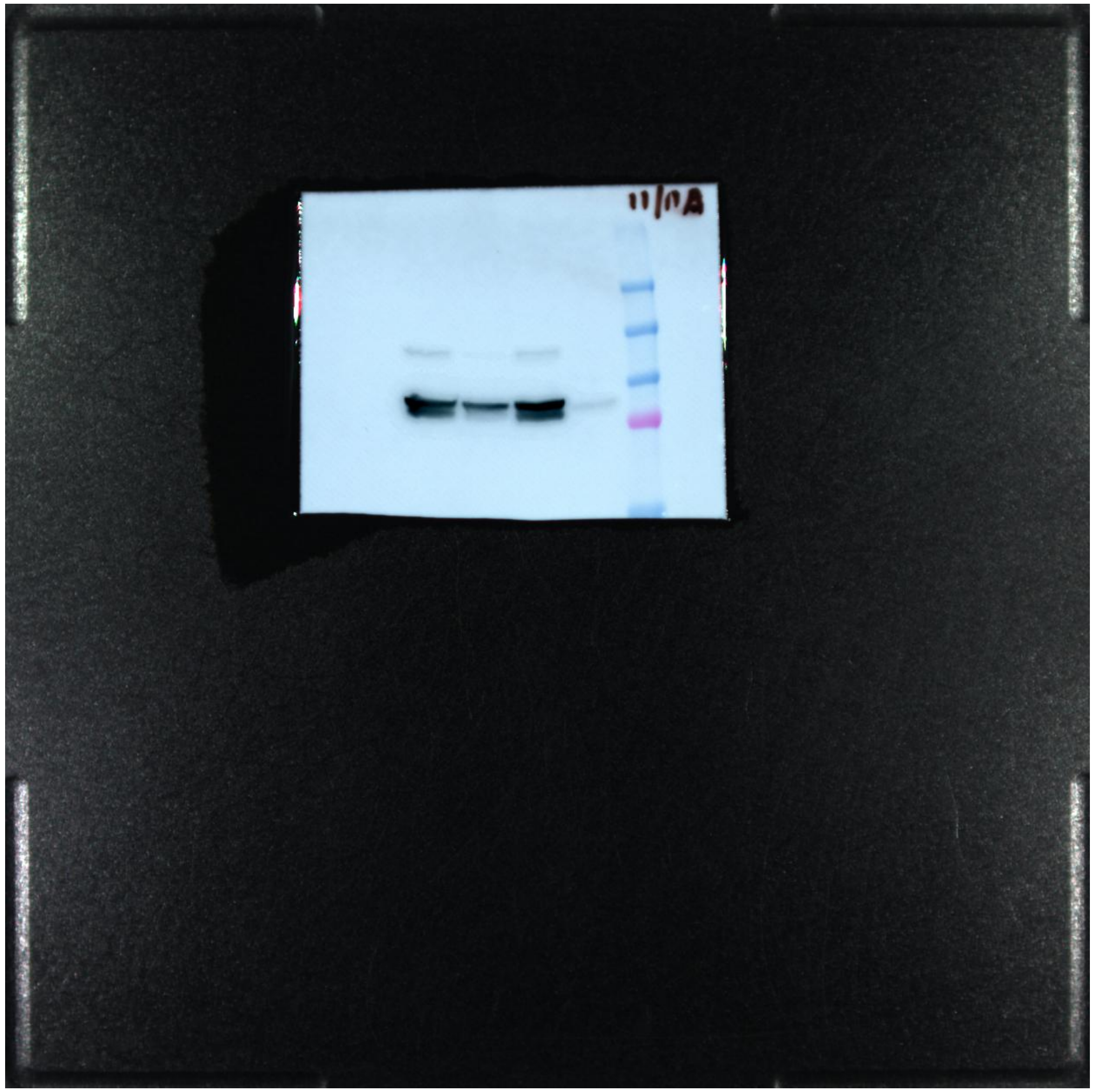

anti-FLAG
Note: the membrane was cut into halves
for probing proteins of different sizes

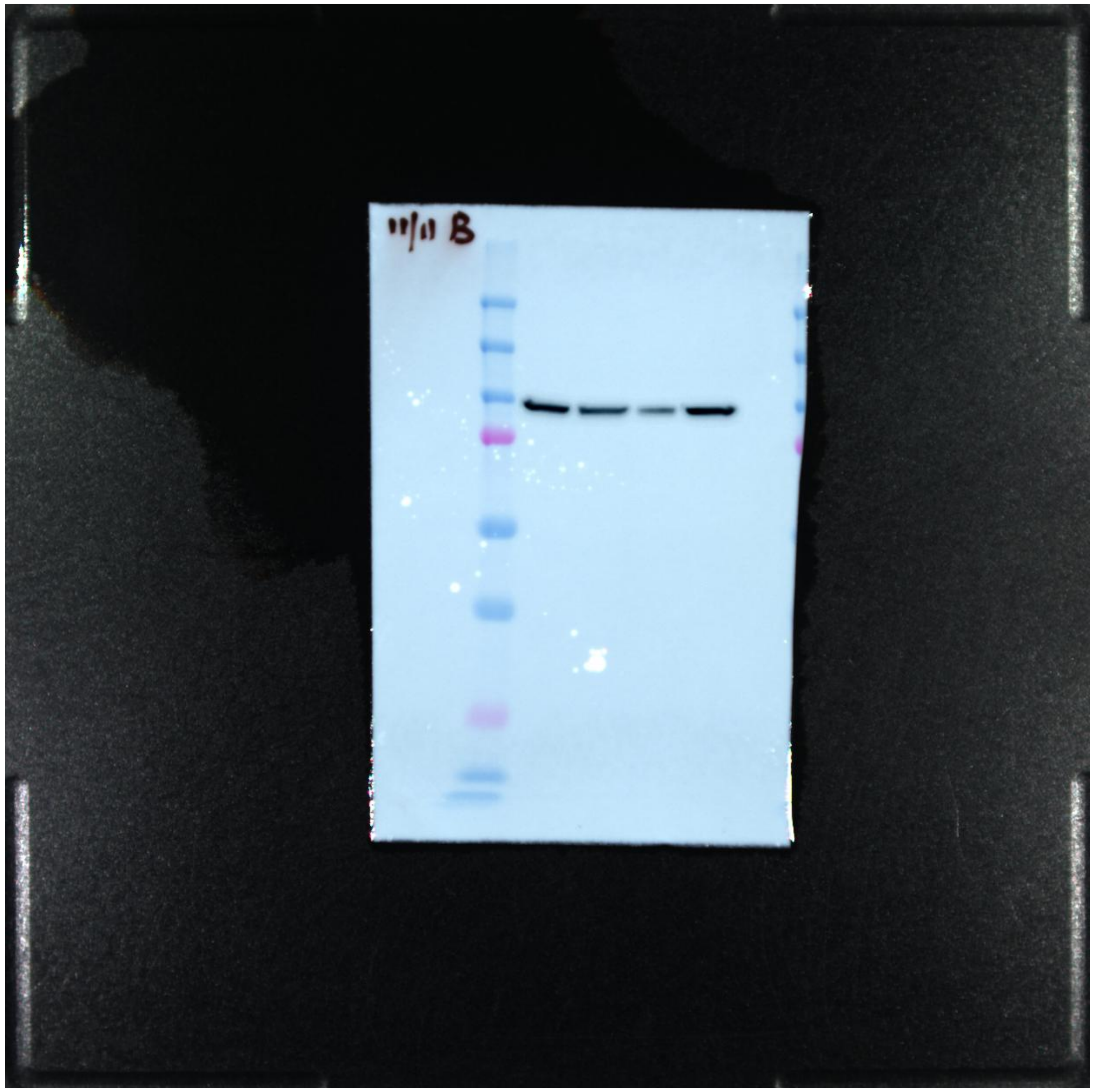

CTNNB1

Figure 4H

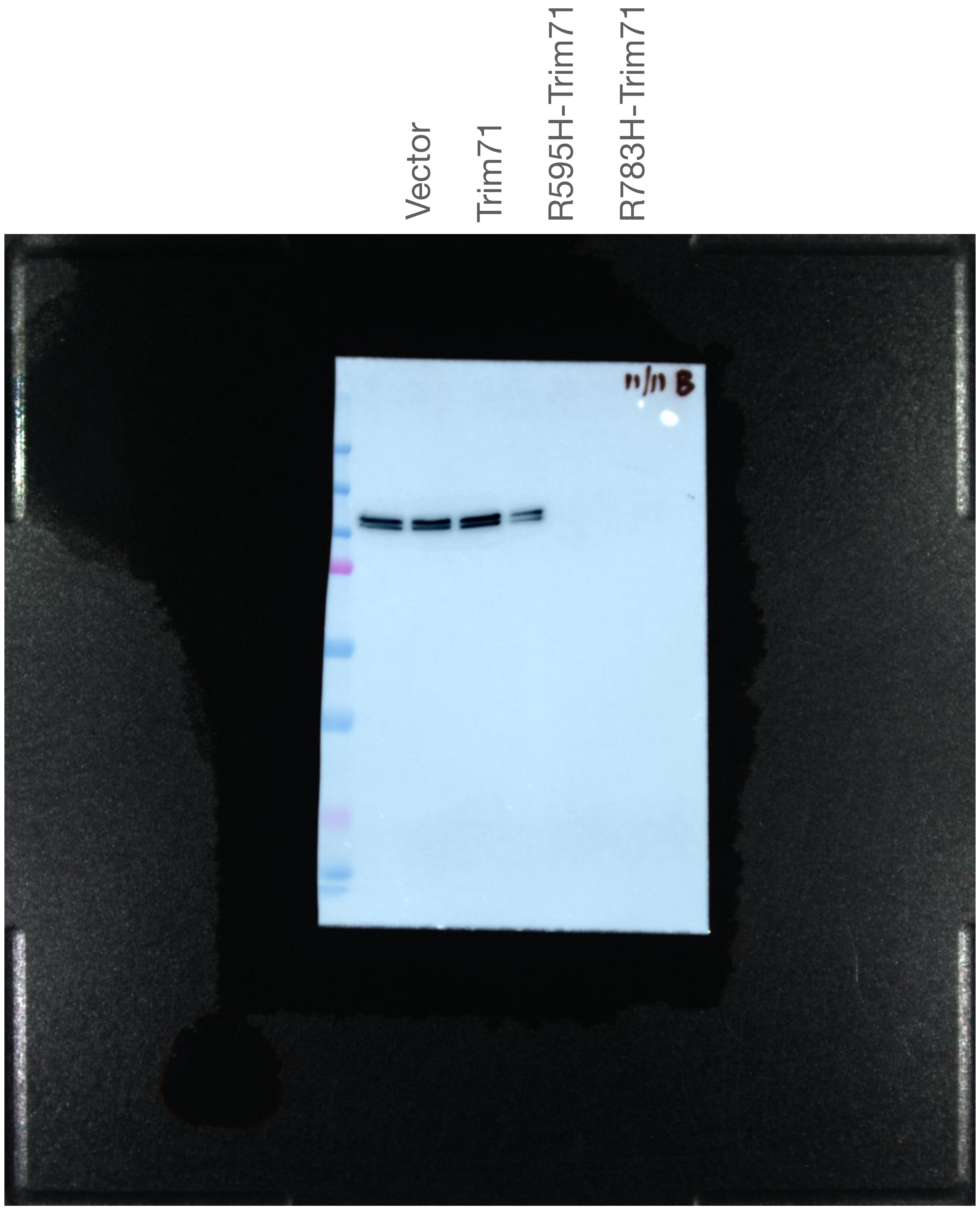

LSD1

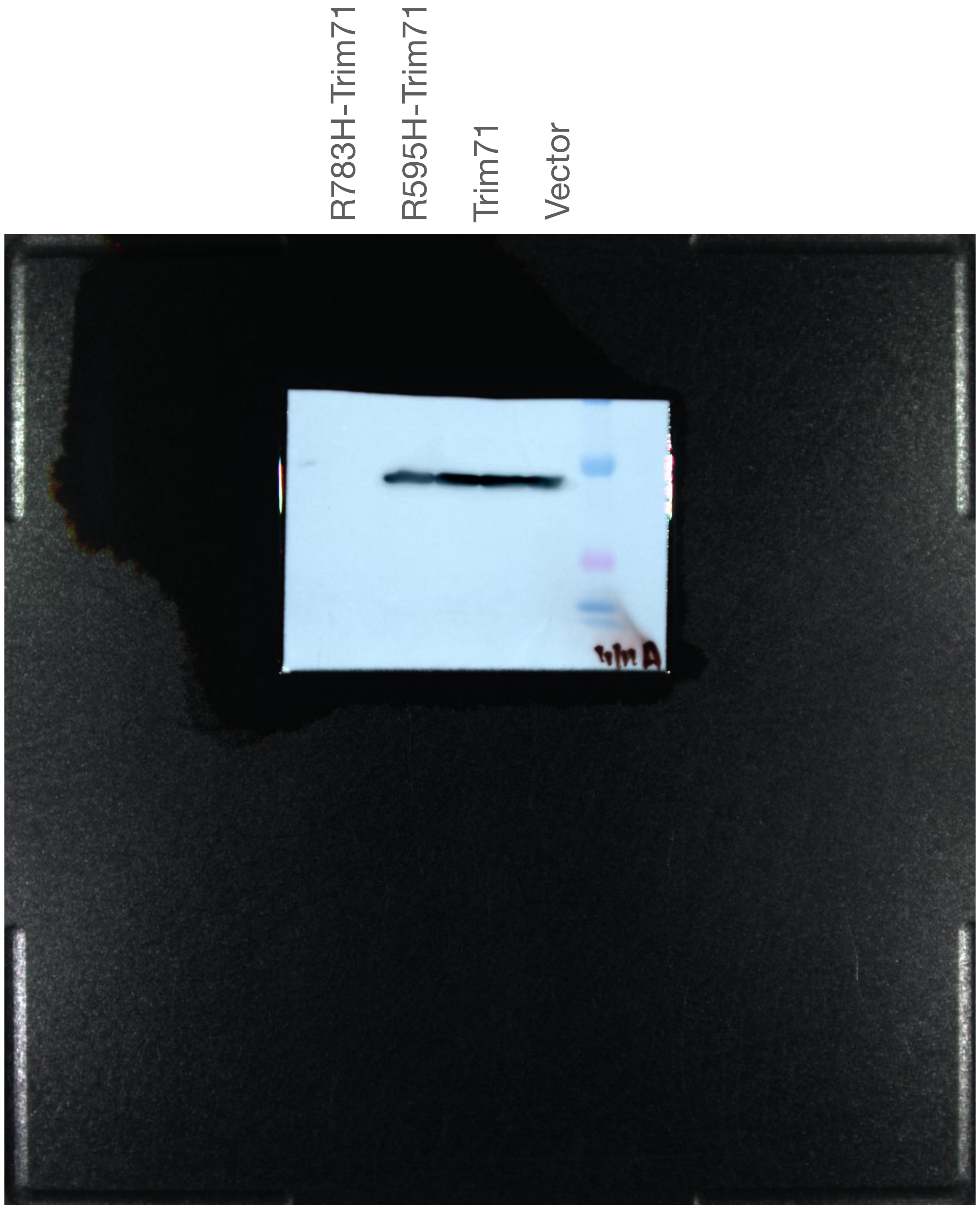

GAPDH
Note: the membrane was cut into halves
for probing proteins of different sizes

Figure 5B

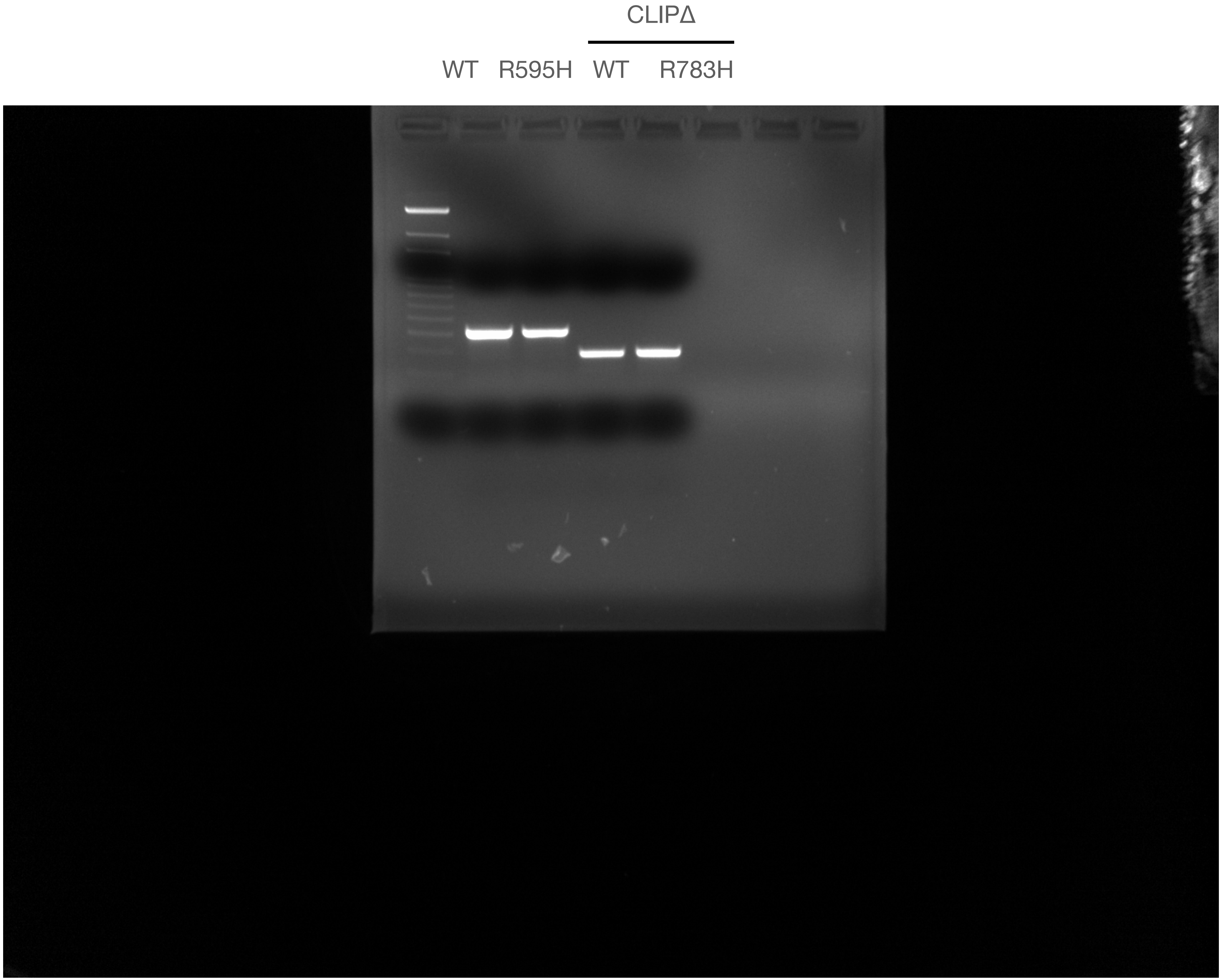

Figure 5D

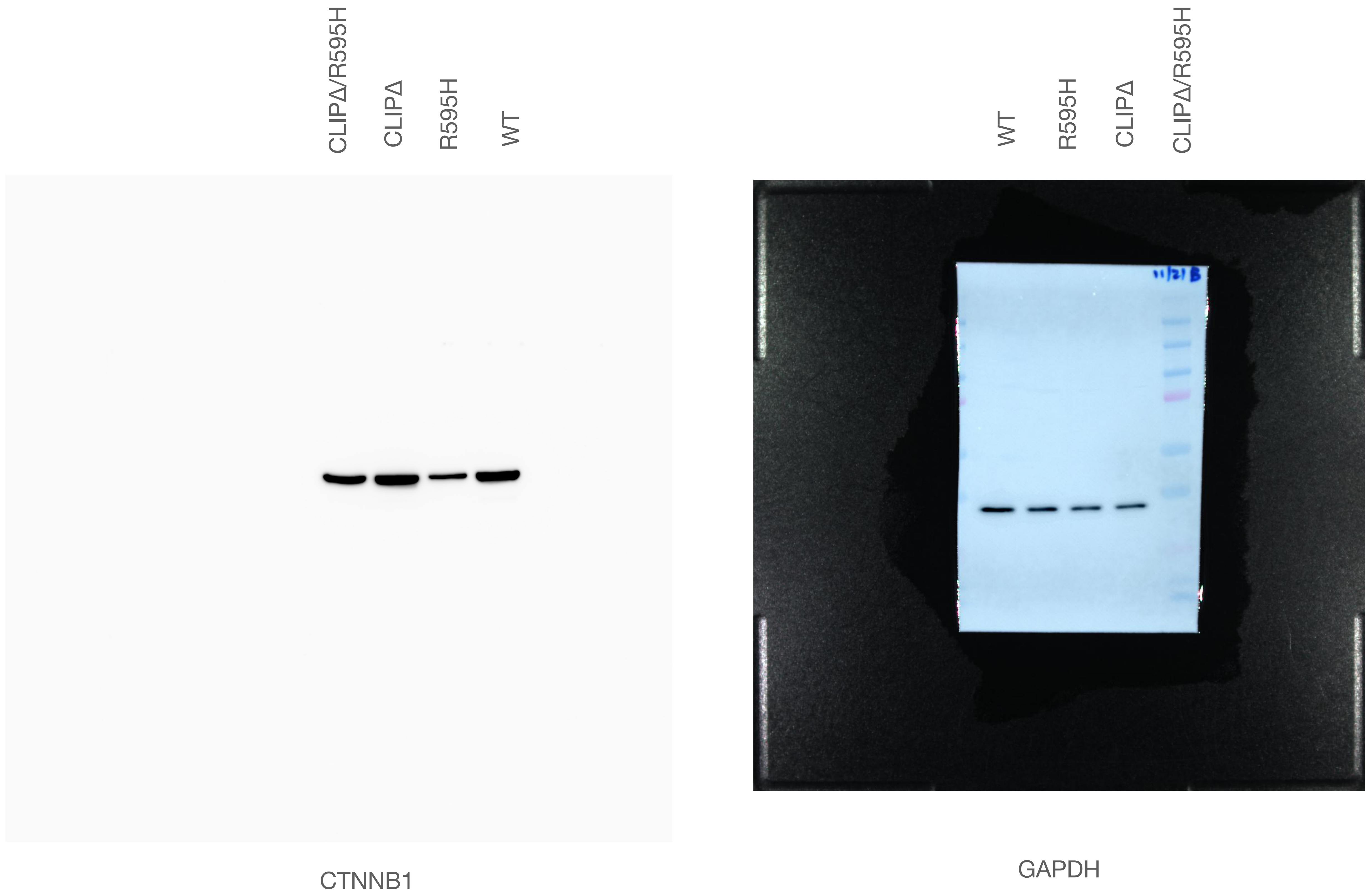
CLIPΔ/R595H
CLIPΔ
R595H
WT
WT
R595H
CLIPΔ
CLIPΔ/R595H
CTNNB1
GAPDH

Figure 5H

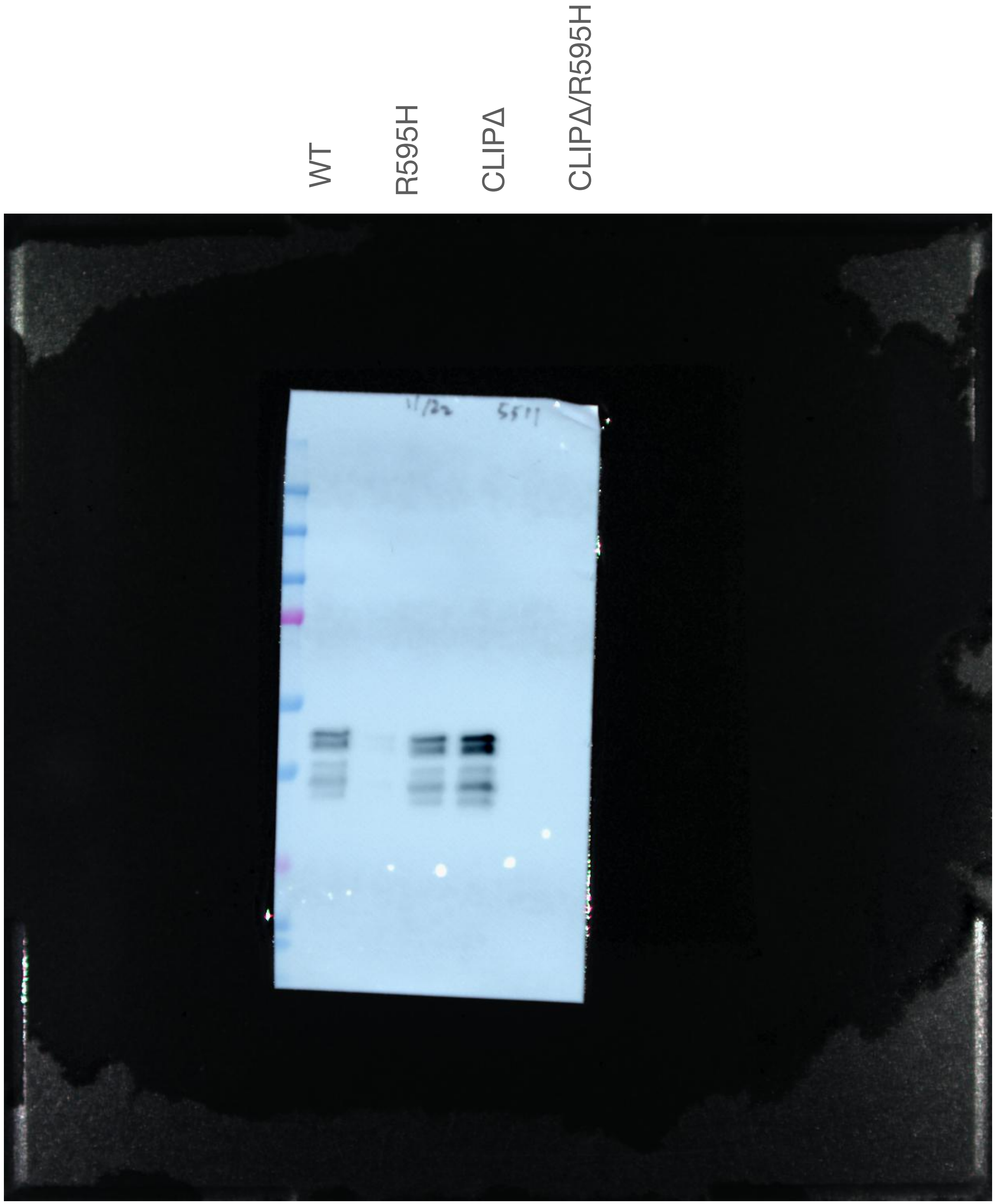

NANOG

Figure 5H

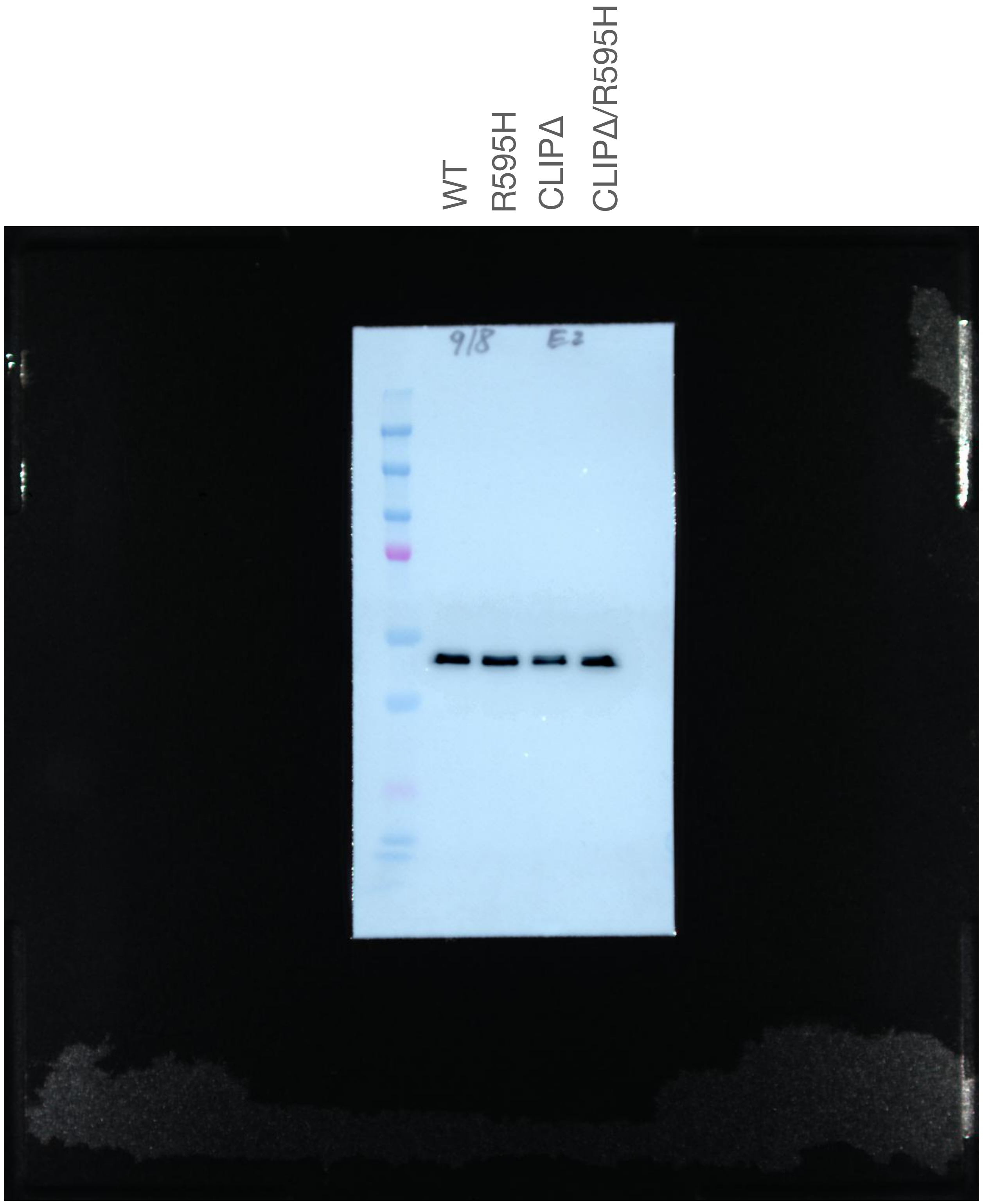

OCT4

Figure 5H

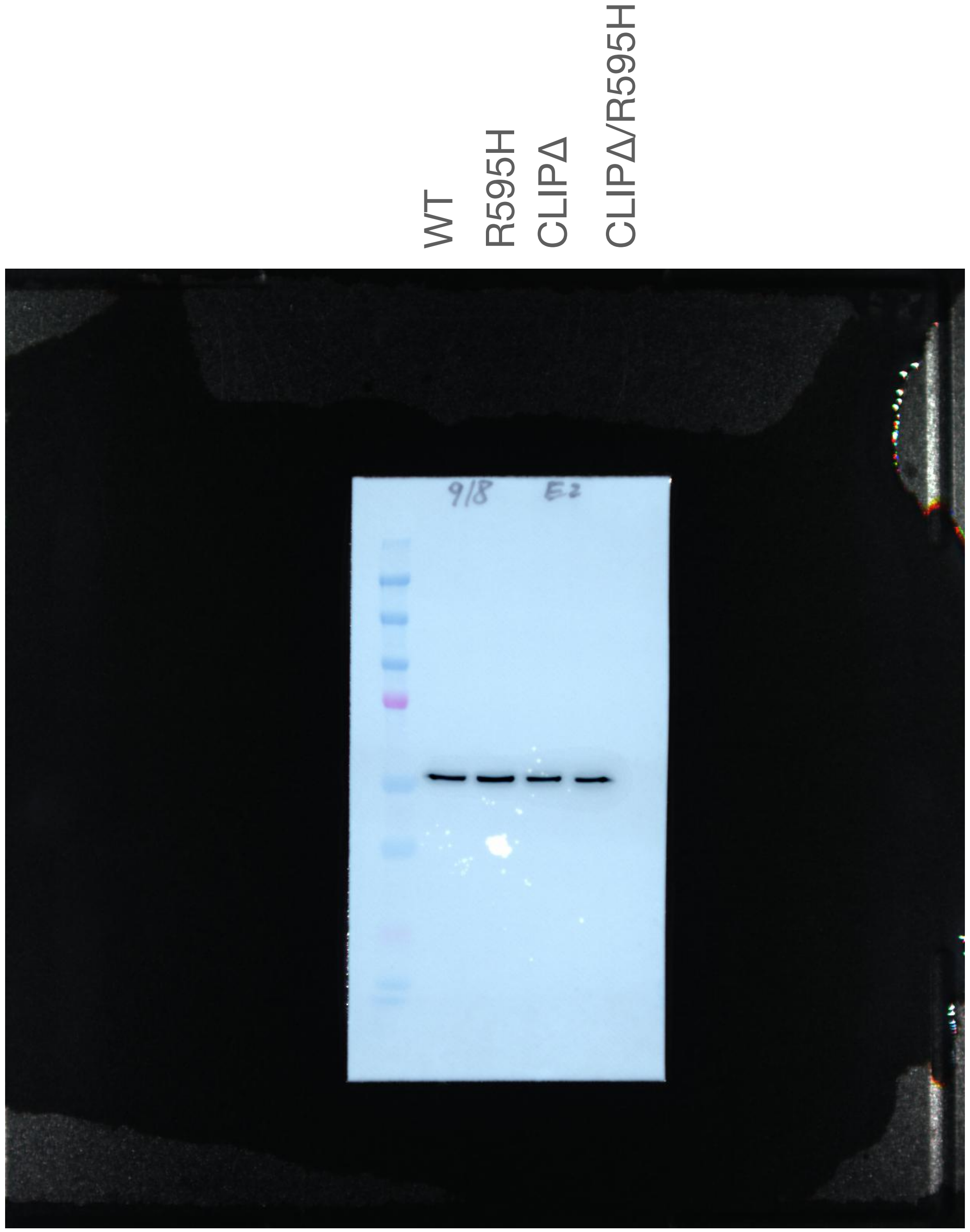

beta-Tubulin

Figure 5I

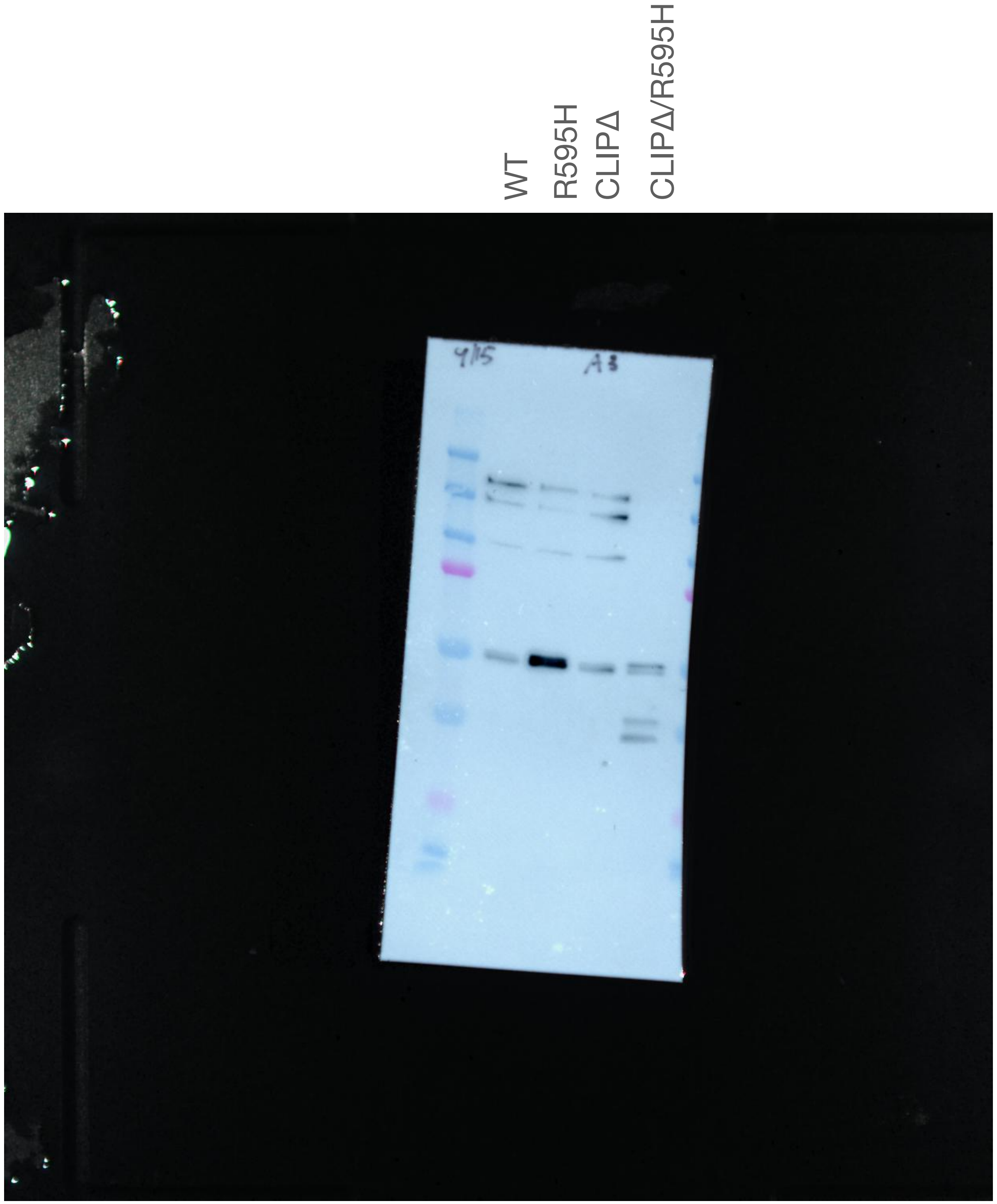

Pax6

Figure 5I

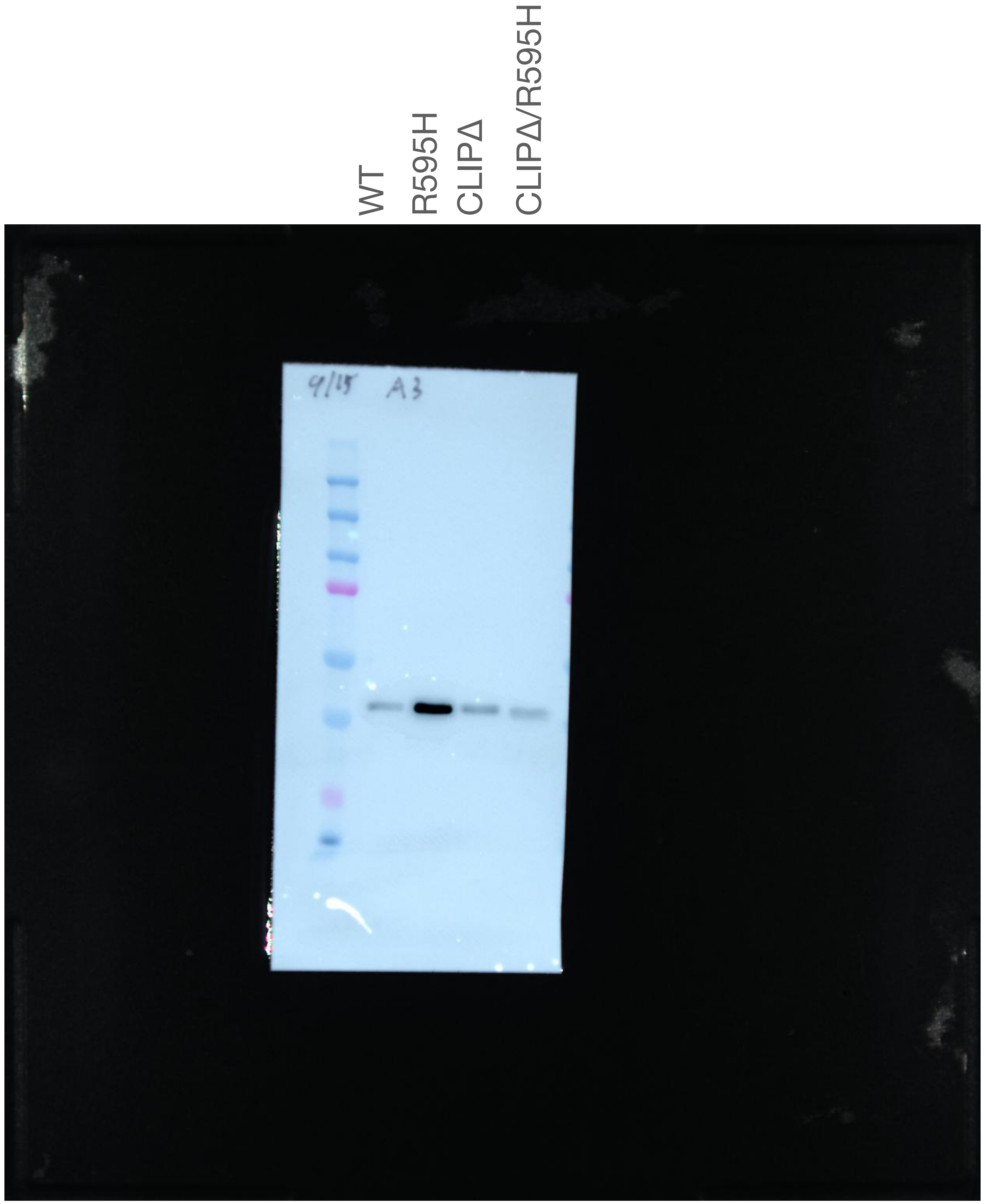

SOX1

Figure 5I

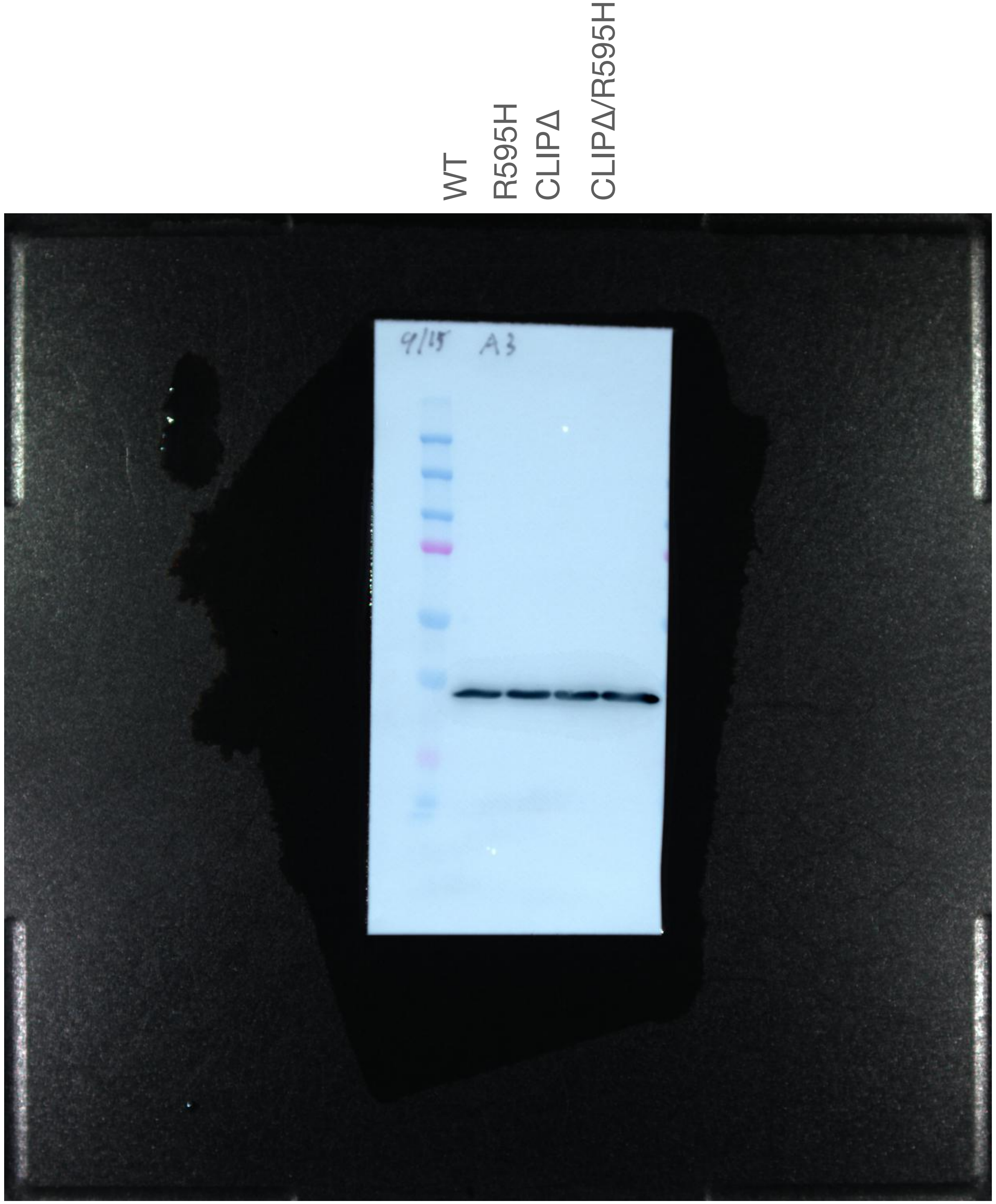

GAPDH

Figure 6B

+ Wnt Agonist

WT R595H R783H WT R595H R783H

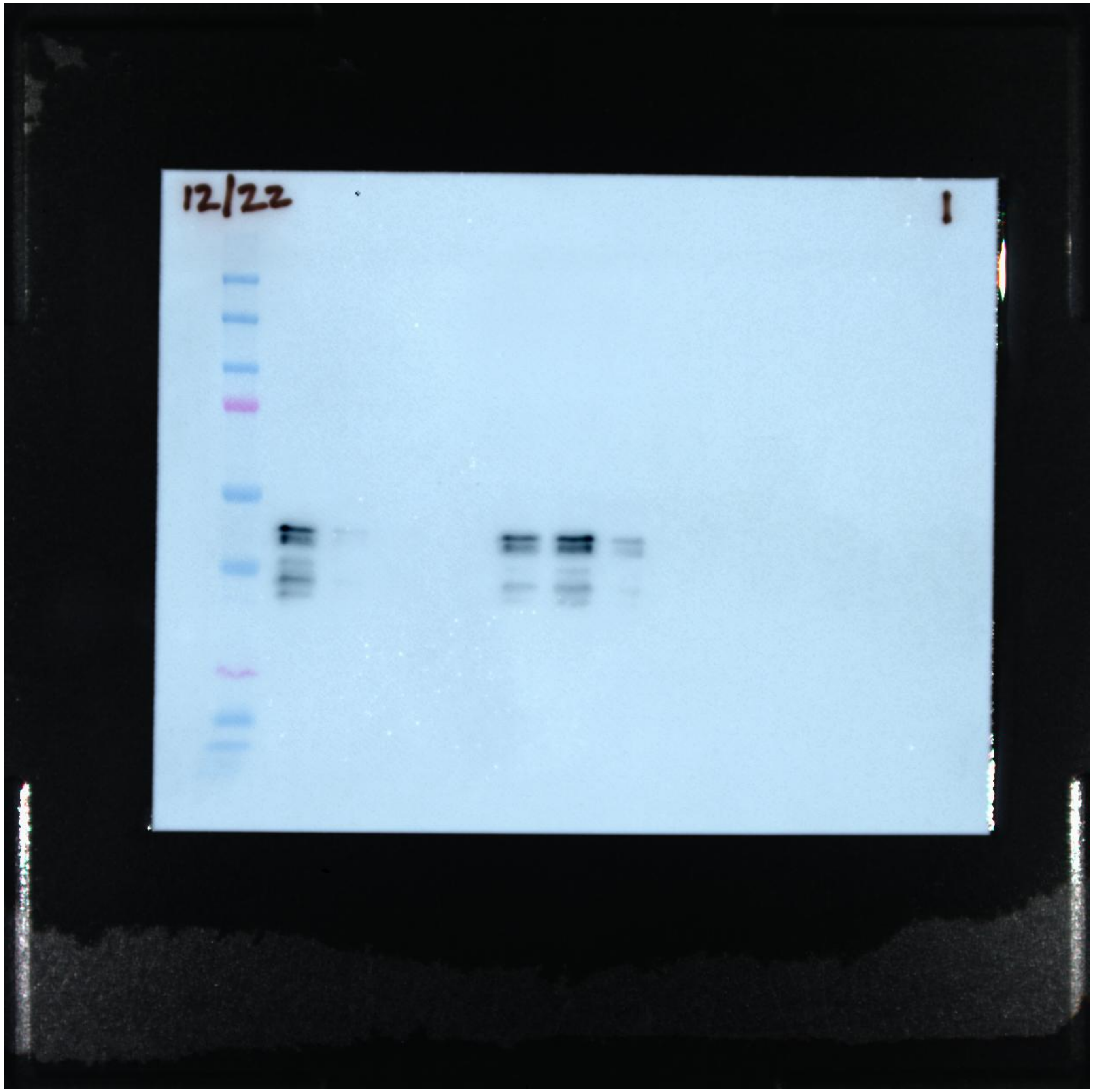

NANOG

Figure 6B

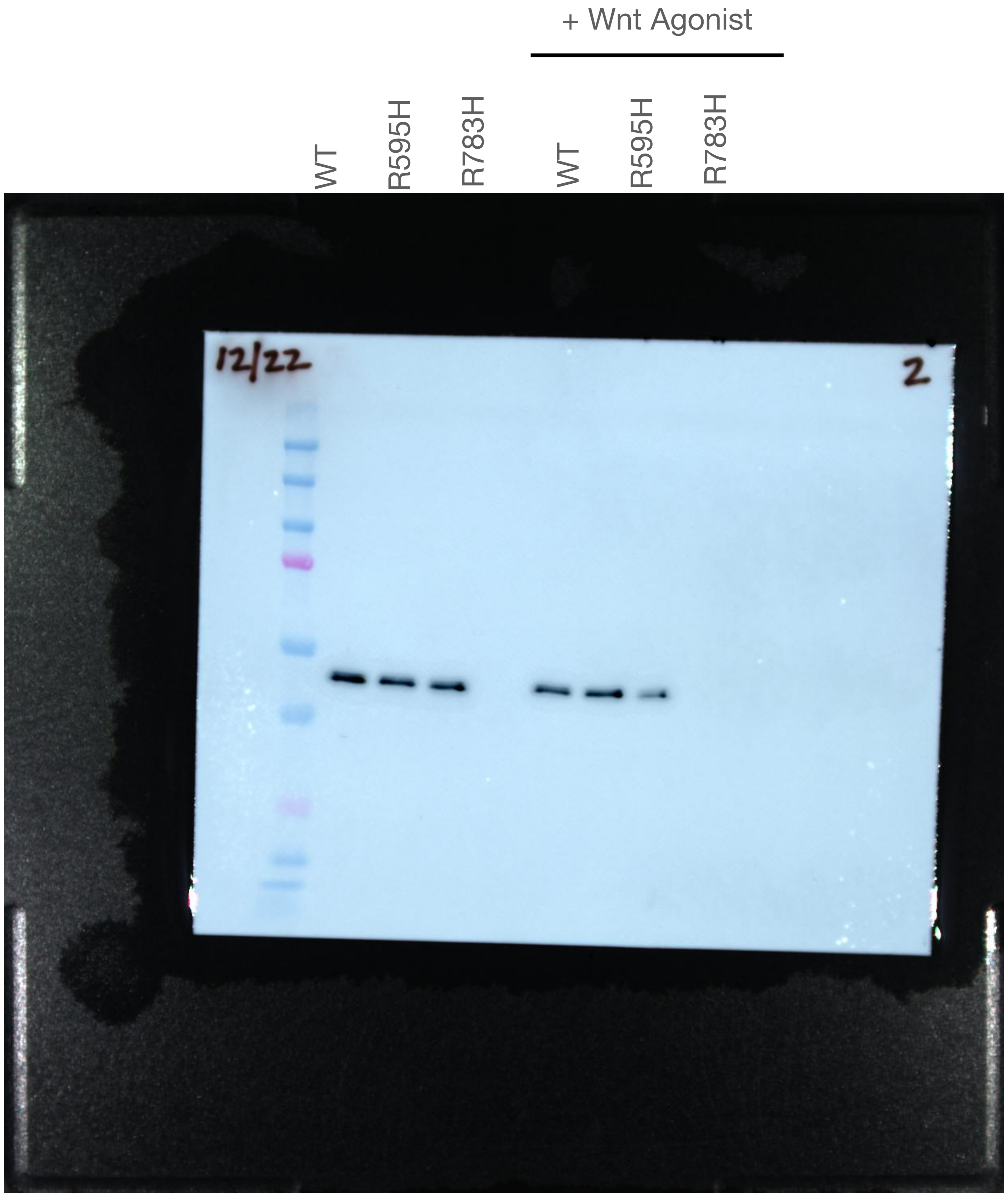

OCT4

Figure 6B

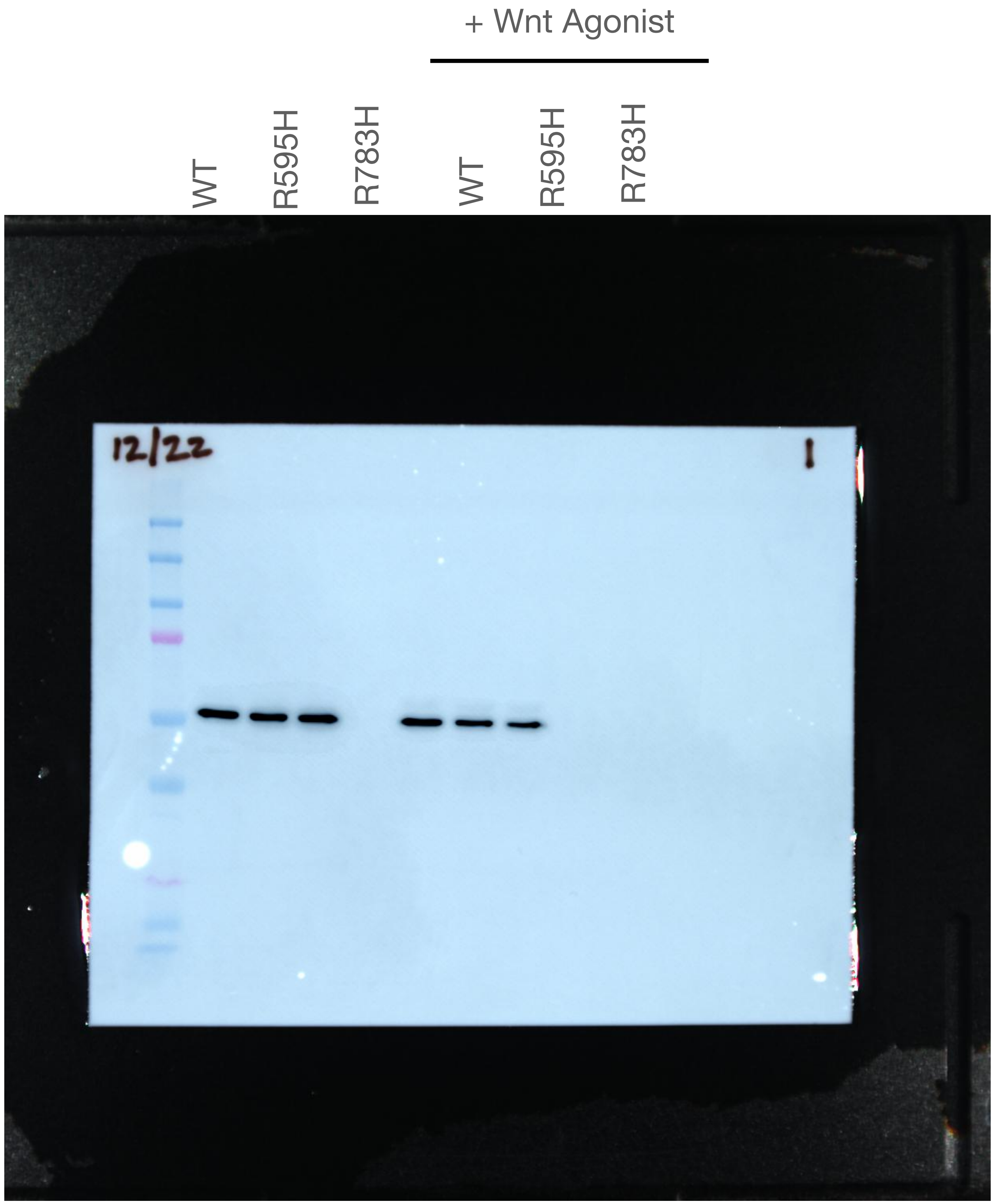

beta-TUBULIN

Figure 6C

Vector		Ctnnb1-GFP					
WT	R595H	WT	R595H	X	X	X	X

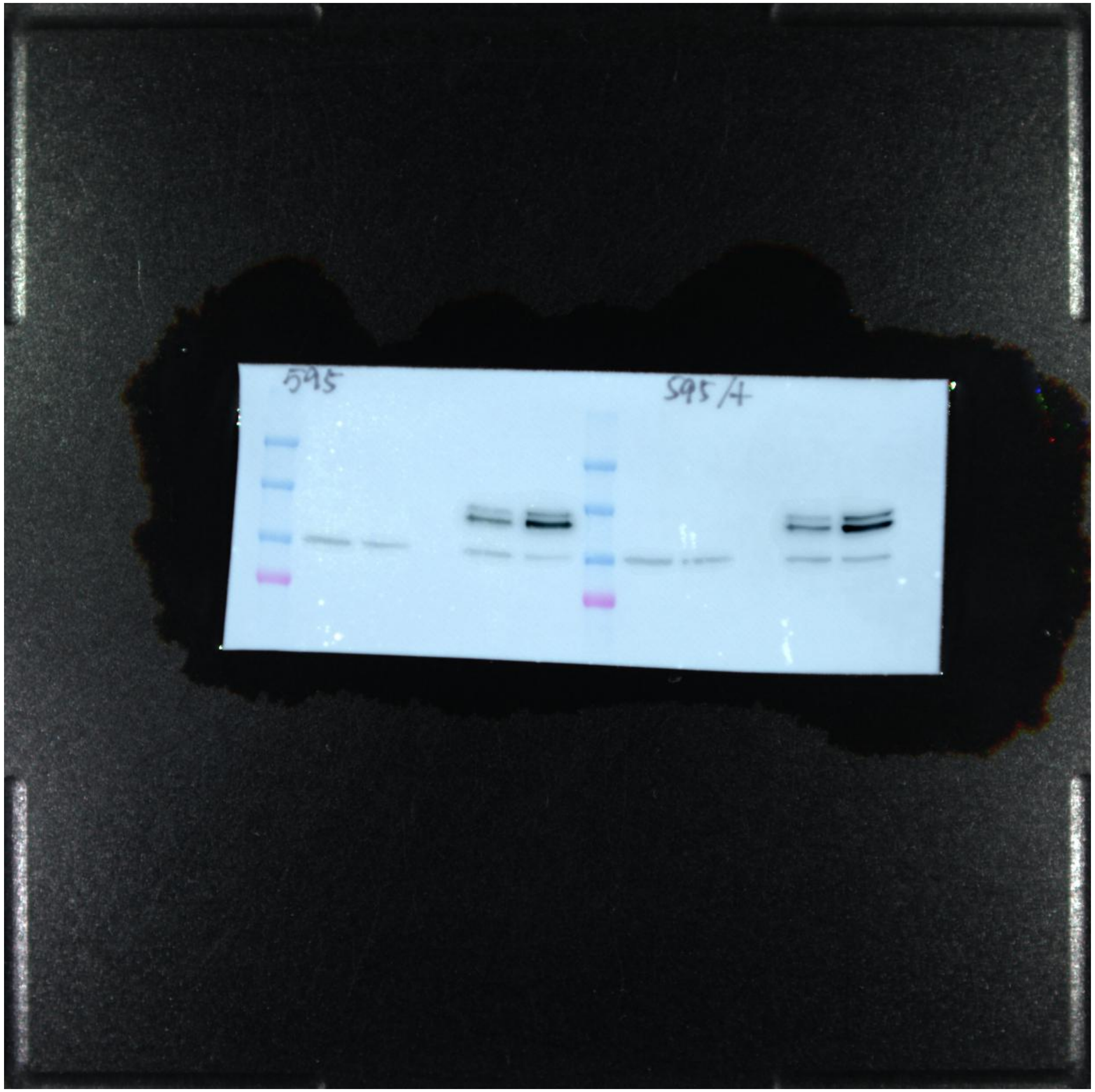

CTNNB1

Note: the gel was cut into halves for probing Ctnnb1 and Gapdh, respectively

Figure 6C

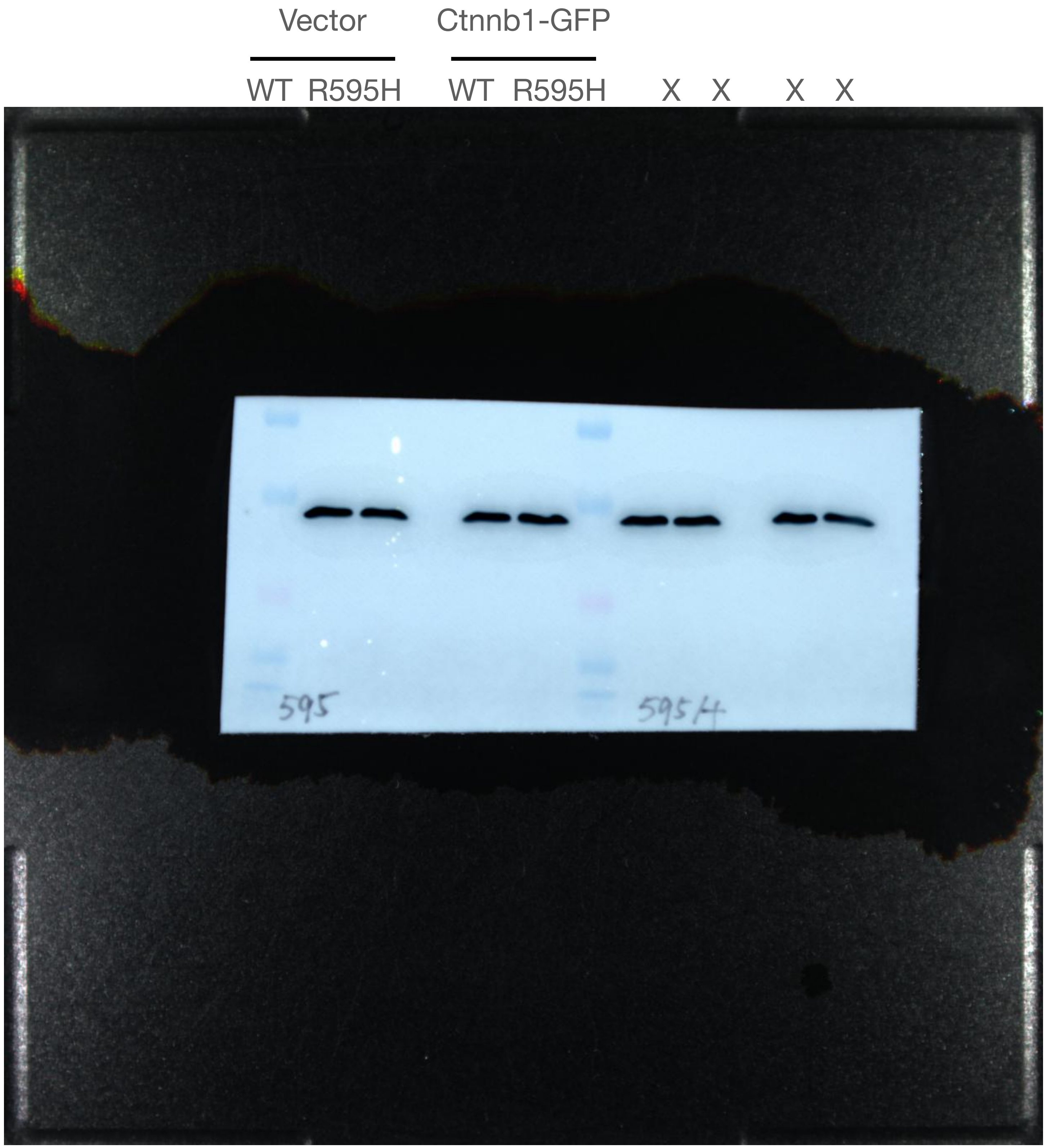

GAPDH

Note: the gel was cut into halves for probing Ctnnb1 and Gapdh, respectively

Figure 6E

Vector Ctnnb1-GFP

WT R595H WT R595H

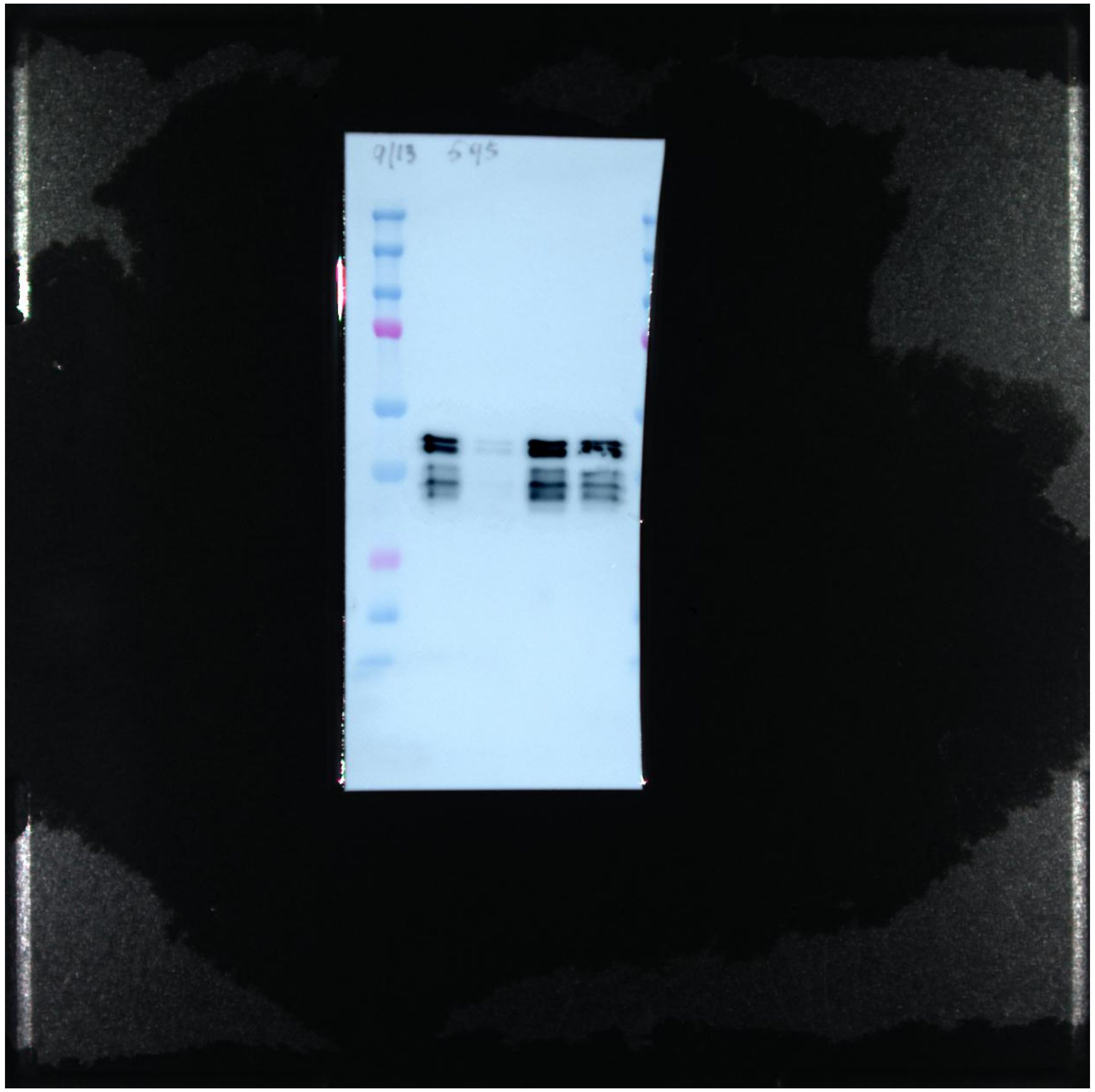

NANOG

Vector Ctnnb1-GFP

WT R595H WT R595H

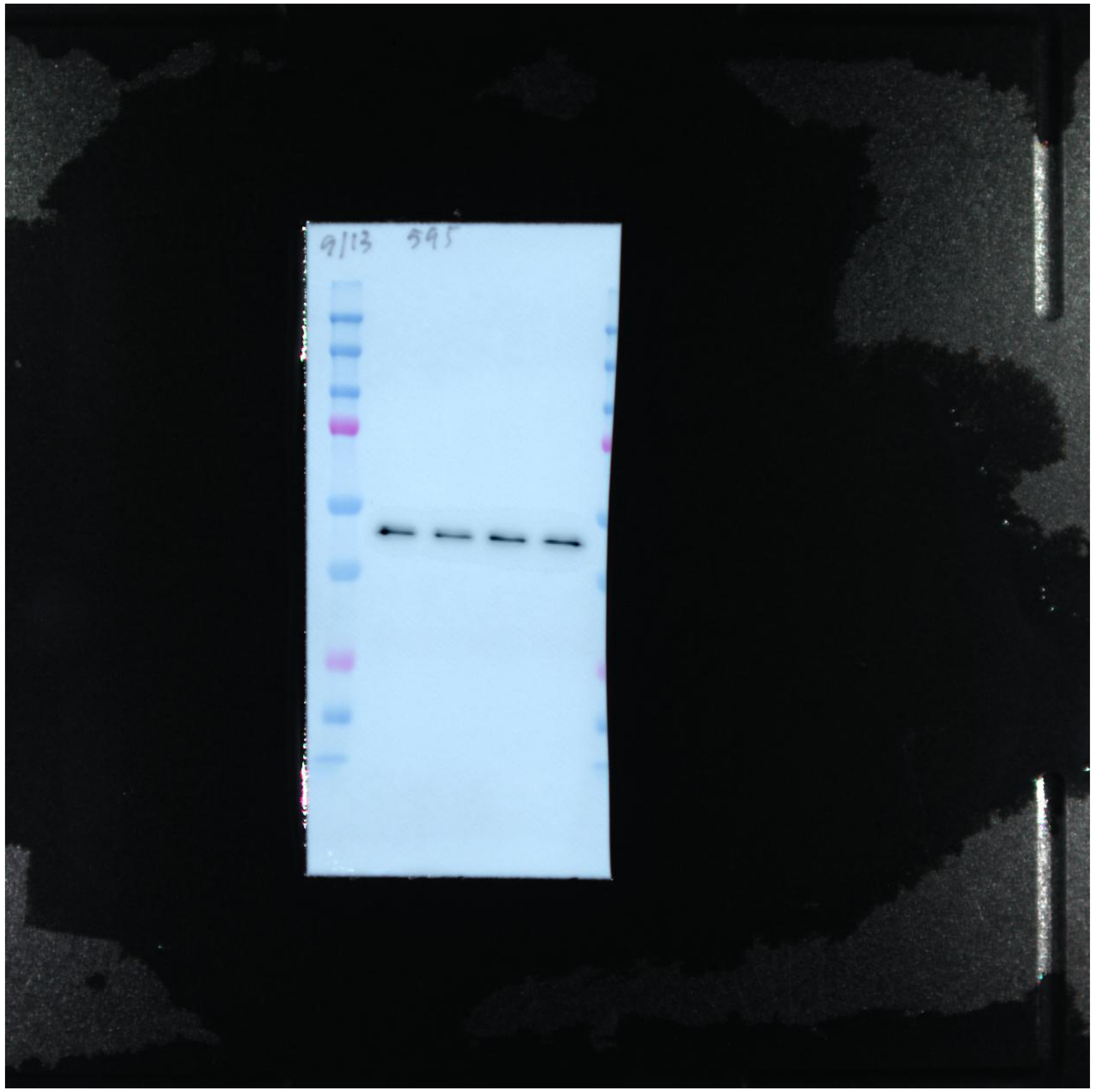

OCT4

Figure 6E

Vector Ctnnb1-GFP

WT R595H WT R595H

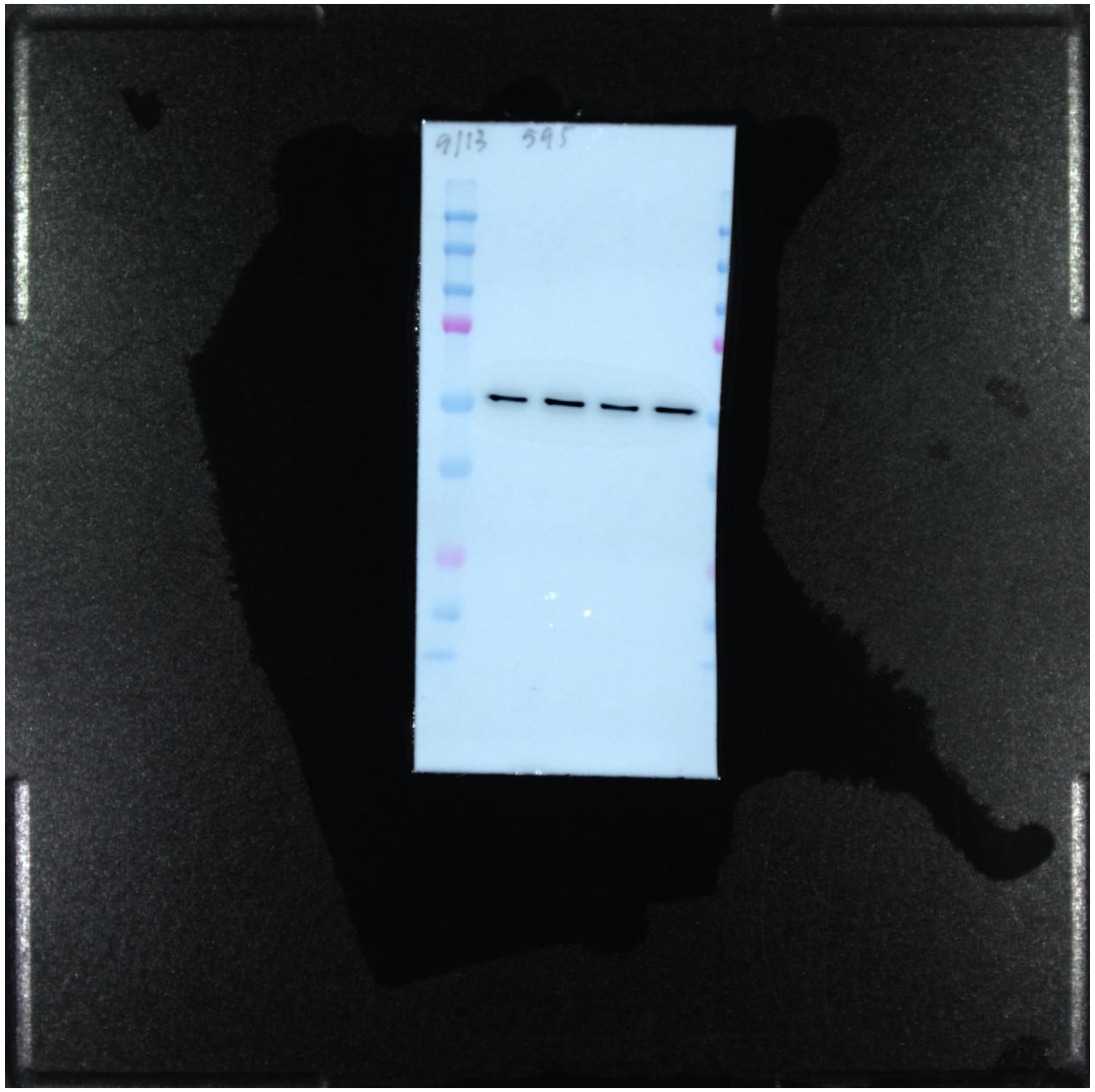

beta-TUBULIN

Figure 6F

Vector Ctnnb1-GFP

WT R595H WT R595H

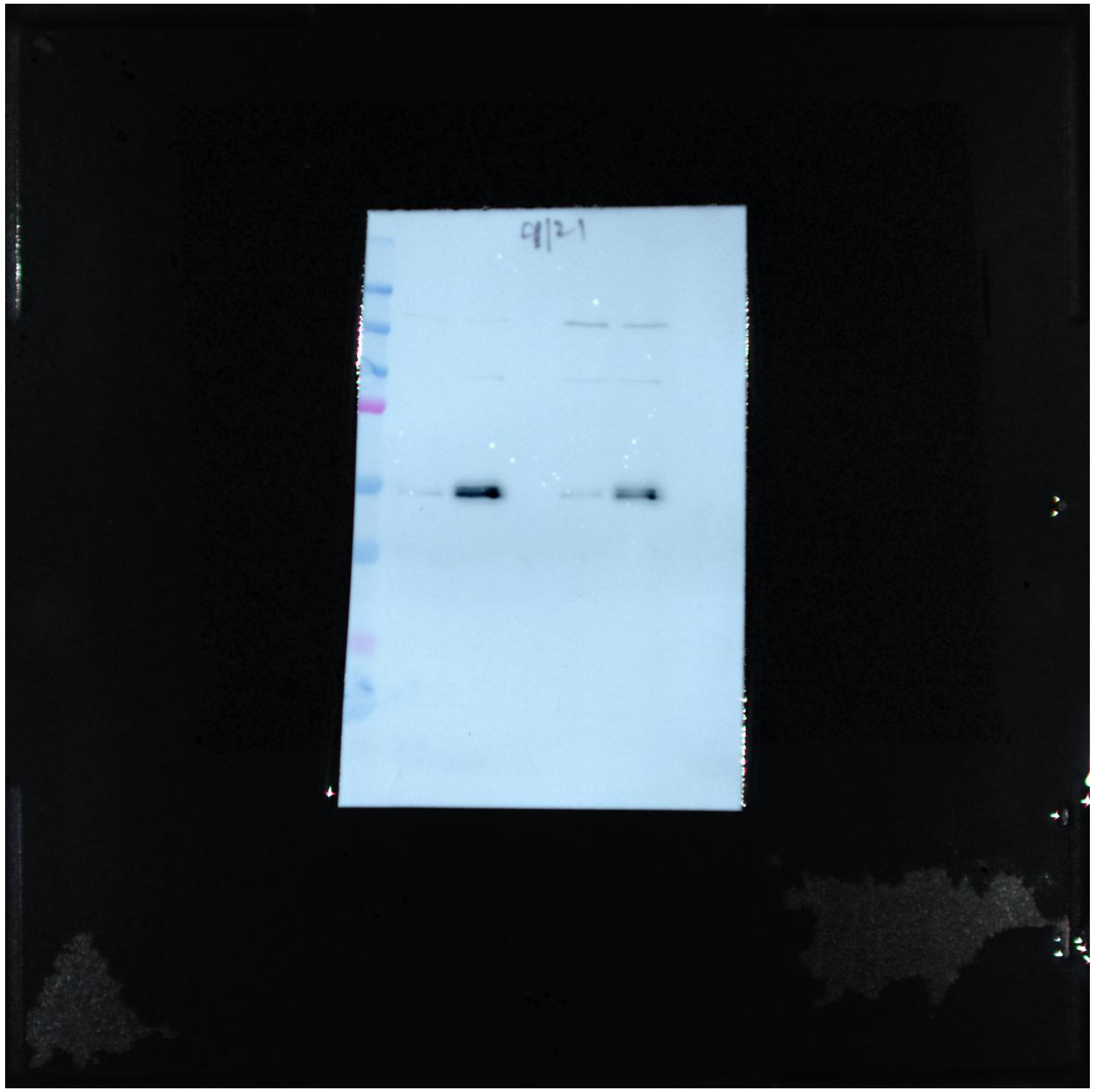

PAX6

Vector Ctnnb1-GFP

WT R595H WT R595H

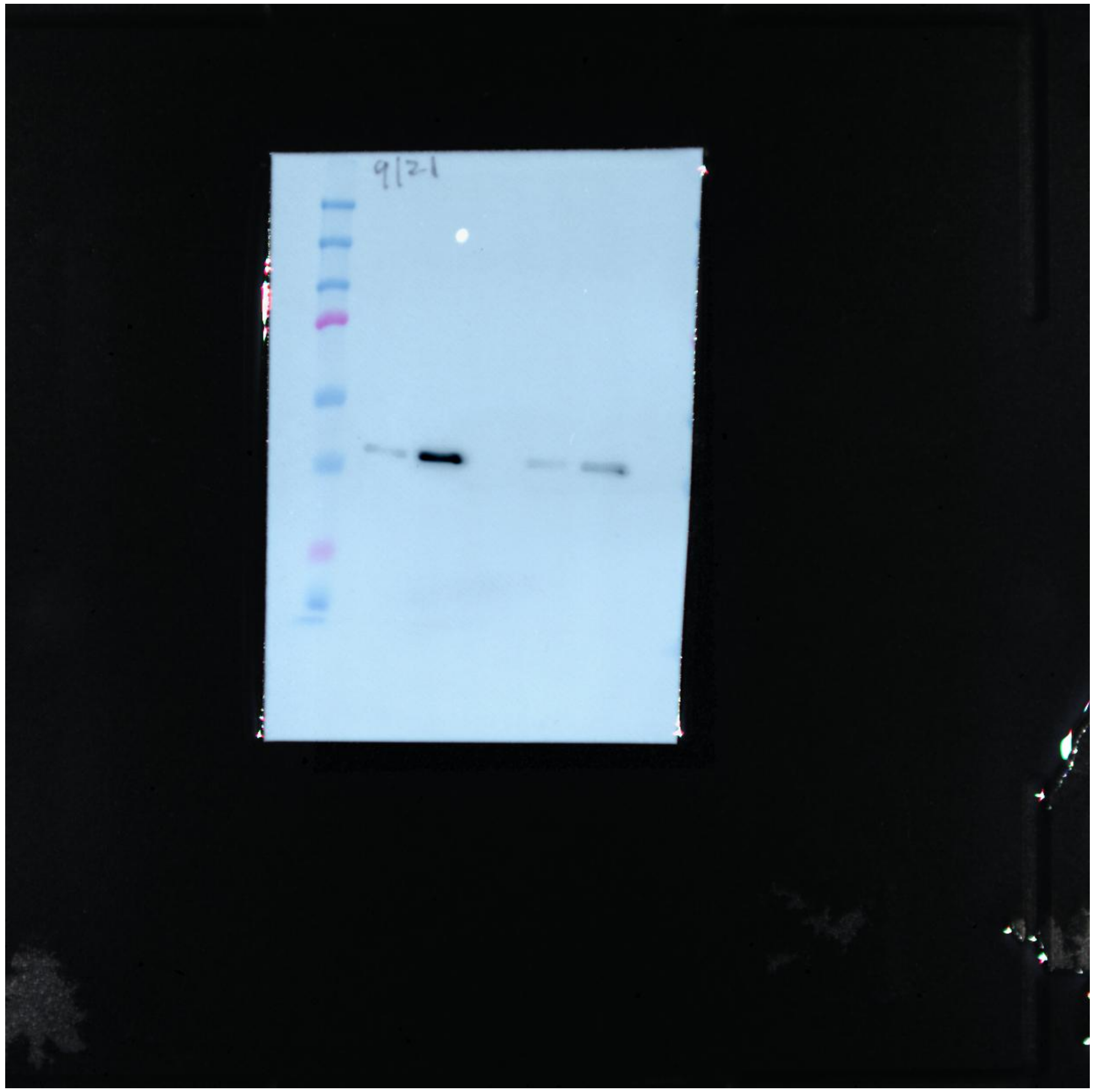

SOX1

Figure 6F

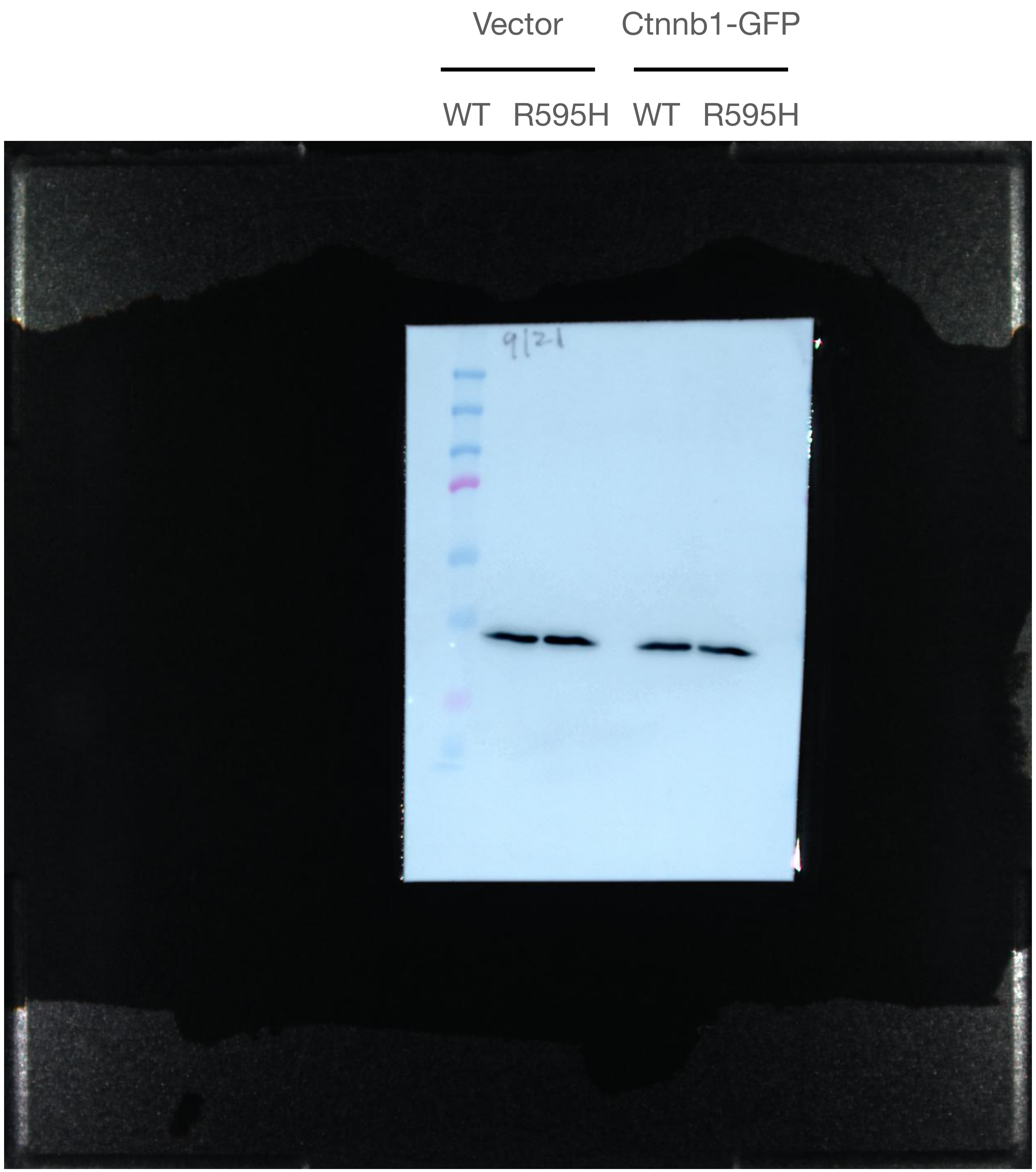

GAPDH

Figure 8A

WT R595H/+

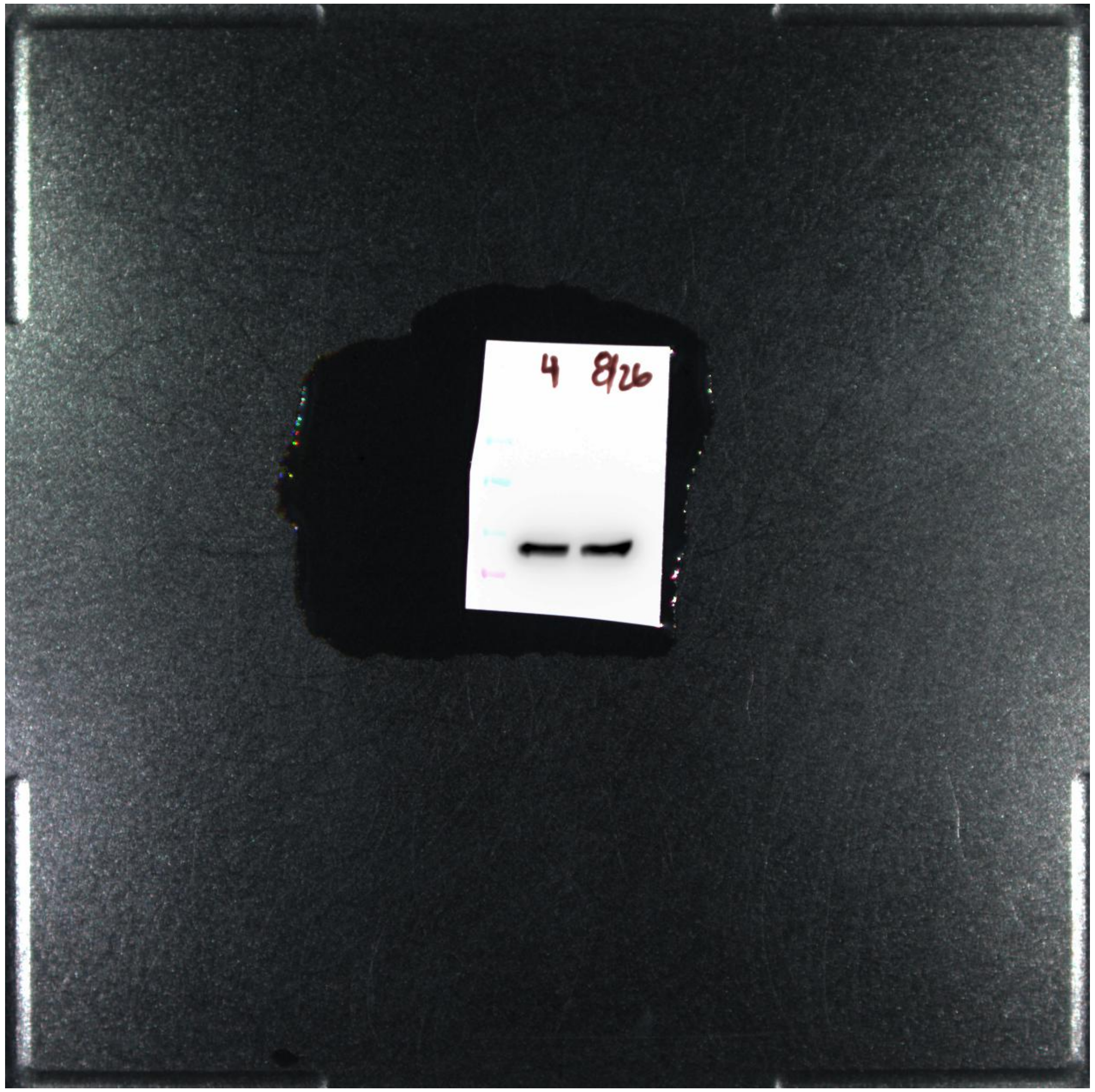

TRIM71

WT R595H/+

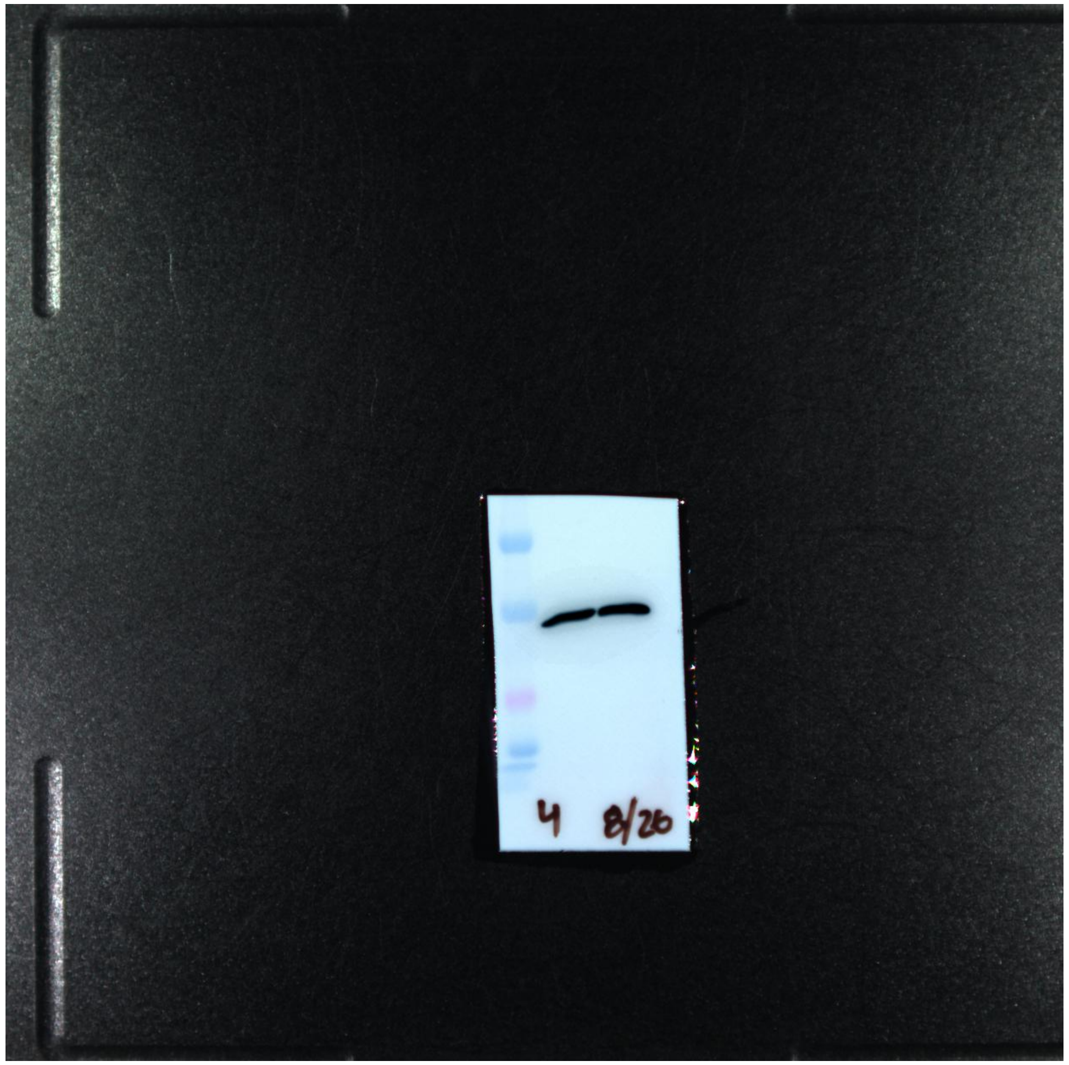

GAPDH

Note: the membrane was cut into halves for probing TRIM71 and GAPDH respectively

Figure 8A

WT R595H/+

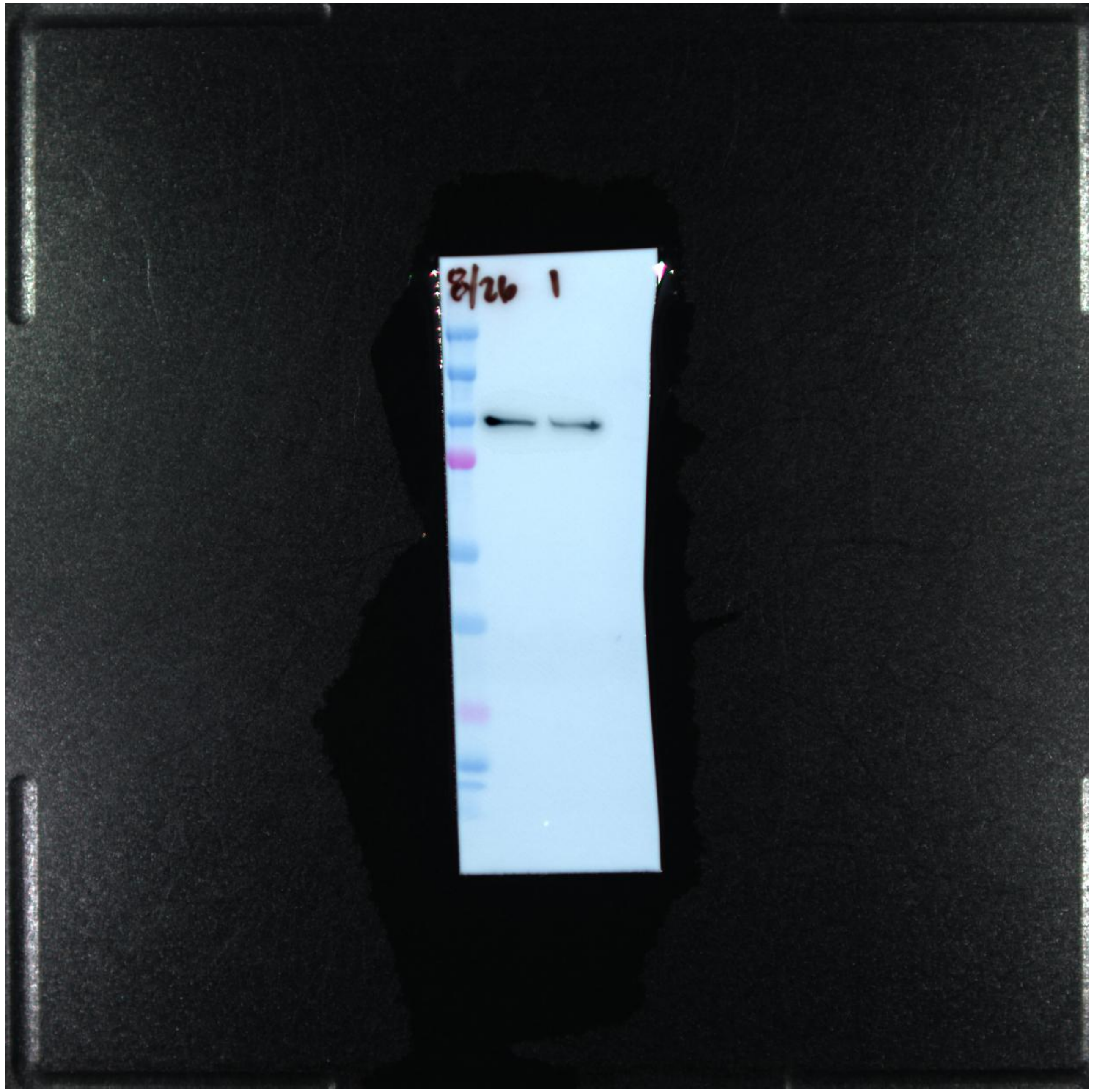

CTNNB1

Figure 8C

WT R595H/+

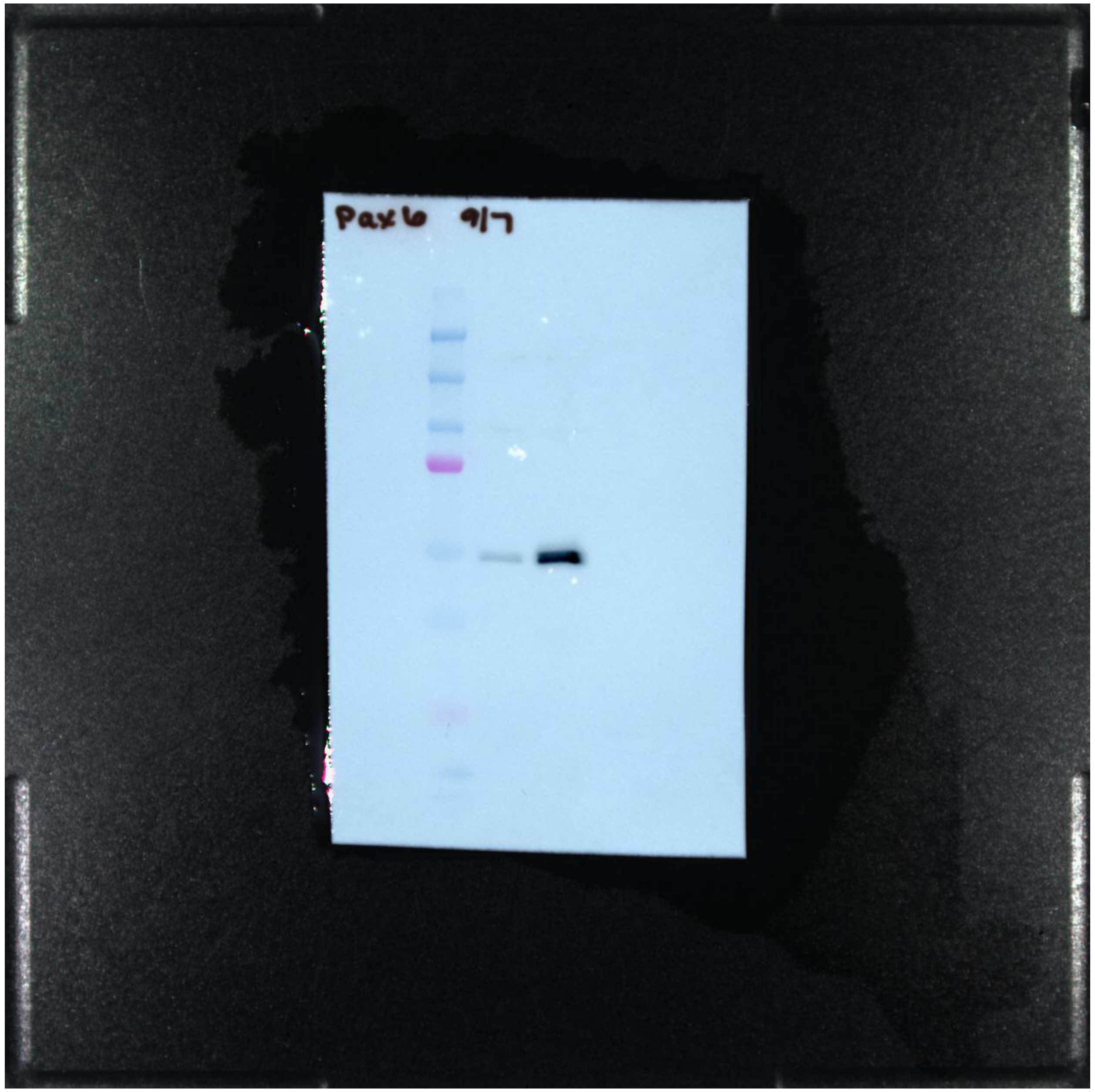

Pax6

Figure 8C

WT R595H/+

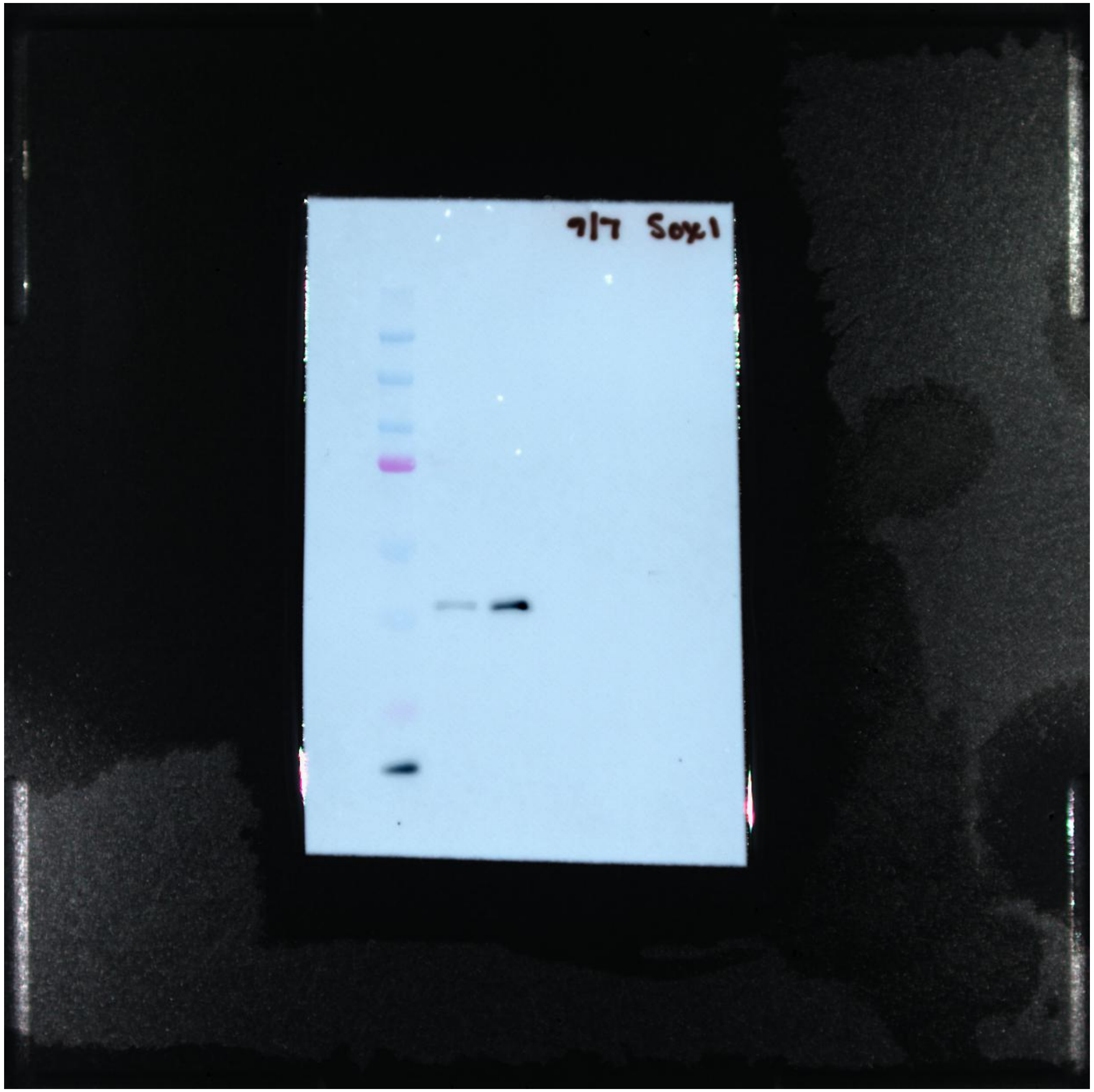

Sox1

Figure 8C

WT   R595H/+

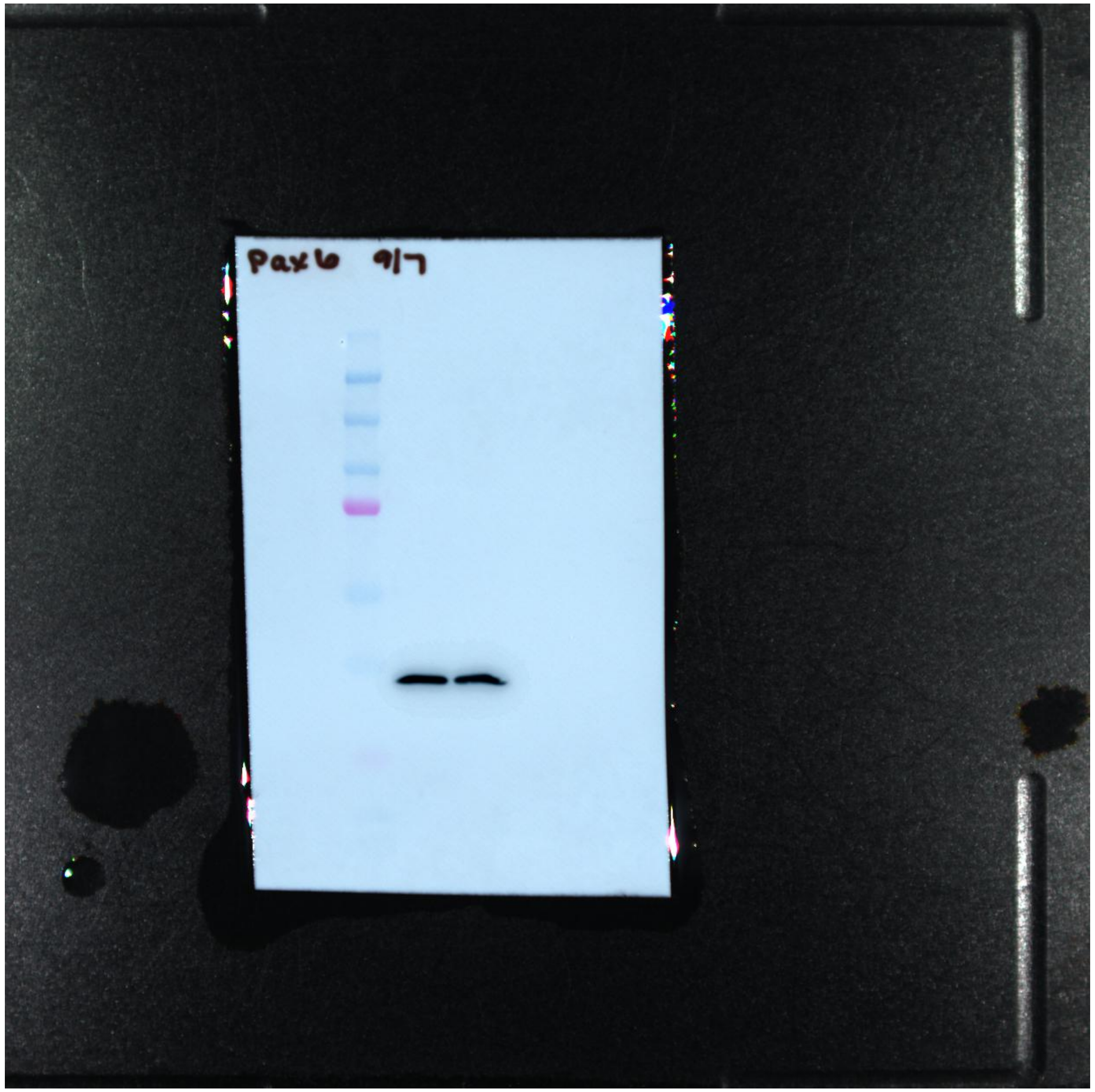

GAPDH

Figure 8D

				Vector		Ctnnb1-GFP	
X	X	X	X	WT	R595H/+	WT	R595H/+

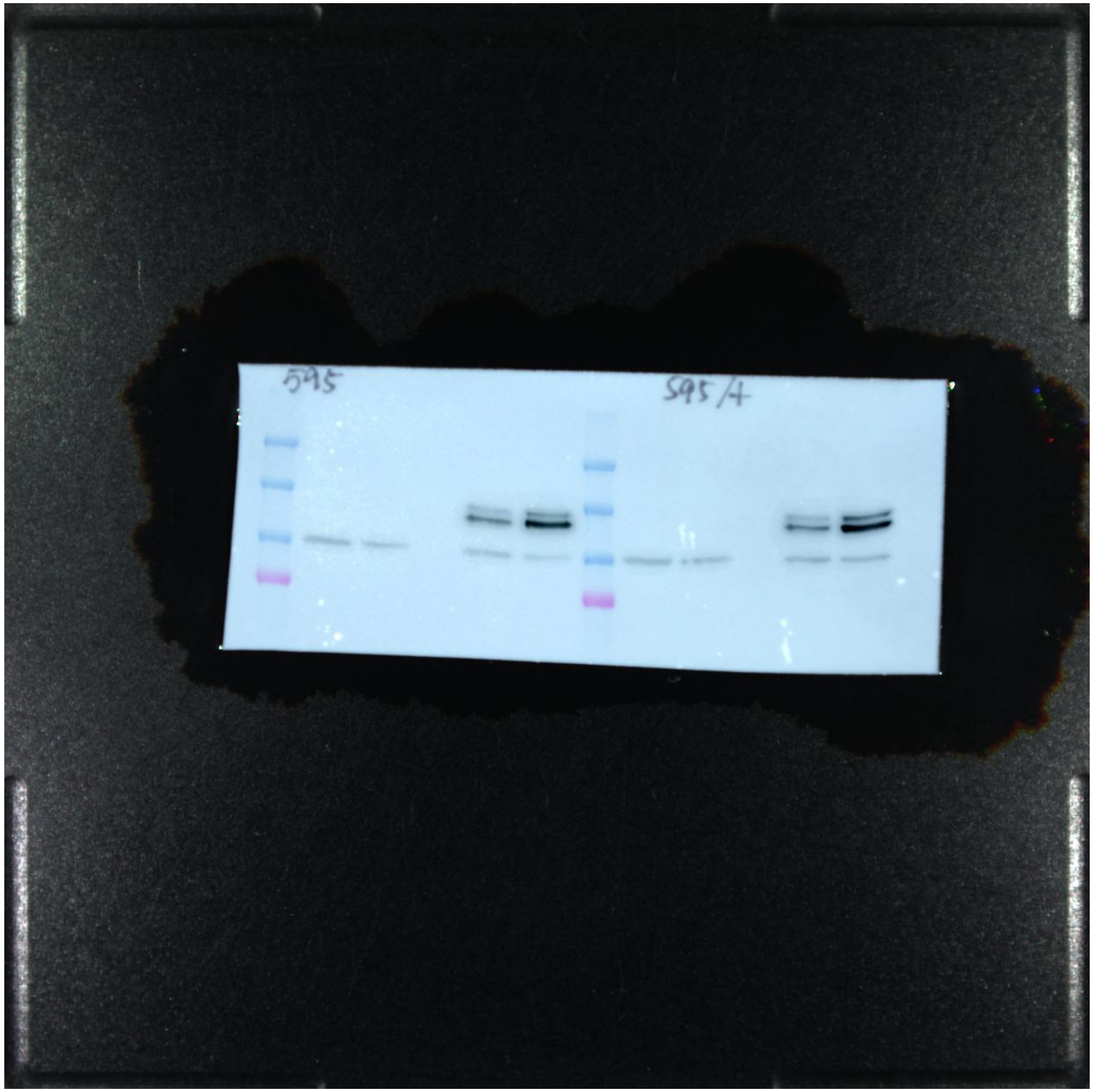

CTNNB1

The membrane was cut into halves for probing CTNNB1 and GAPDH, respectively

Figure 8D

Vector | Ctnnb1-GFP

X X X X WT R595H/+ WT R595H/+

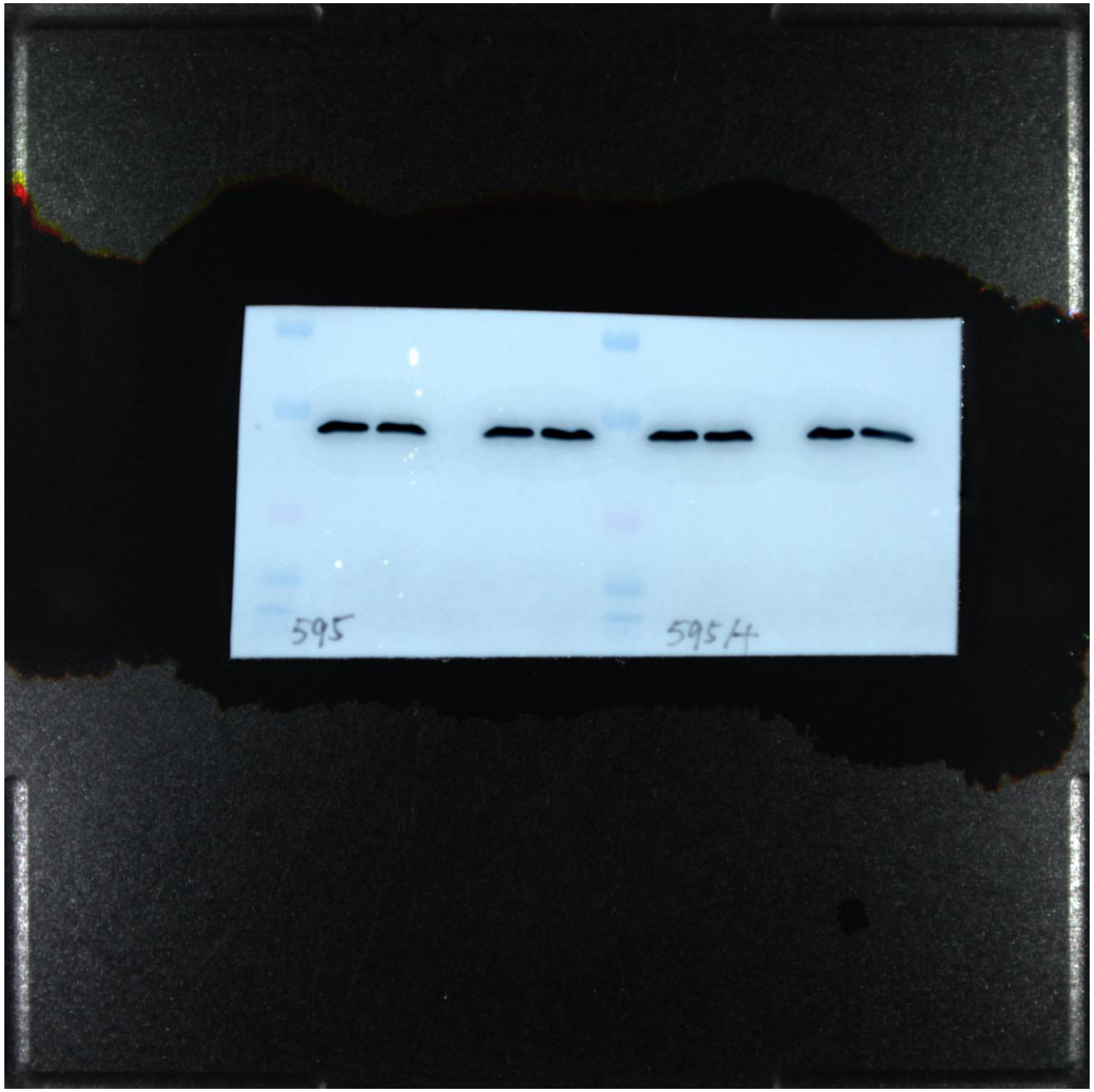

GAPDH

The membrane was cut into halves for probing CTNNB1 and GAPDH, respectively

Figure 8F

Vector | Ctnnb1-GFP

WT R595H/+ WT R595H/+

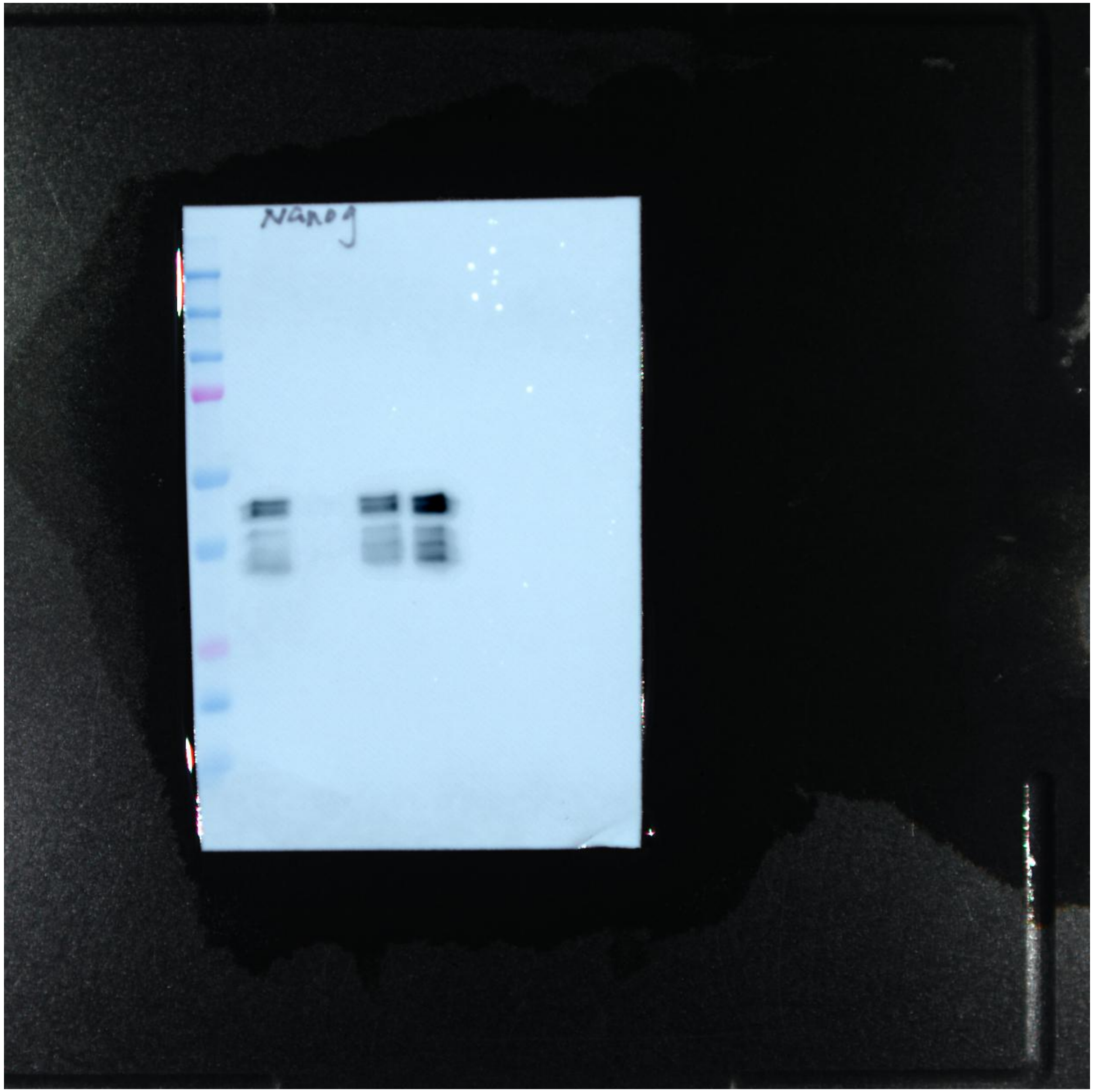

NANOG

Figure 8F

Vector | Ctnnb1-GFP

WT R595H/+ WT R595H/+

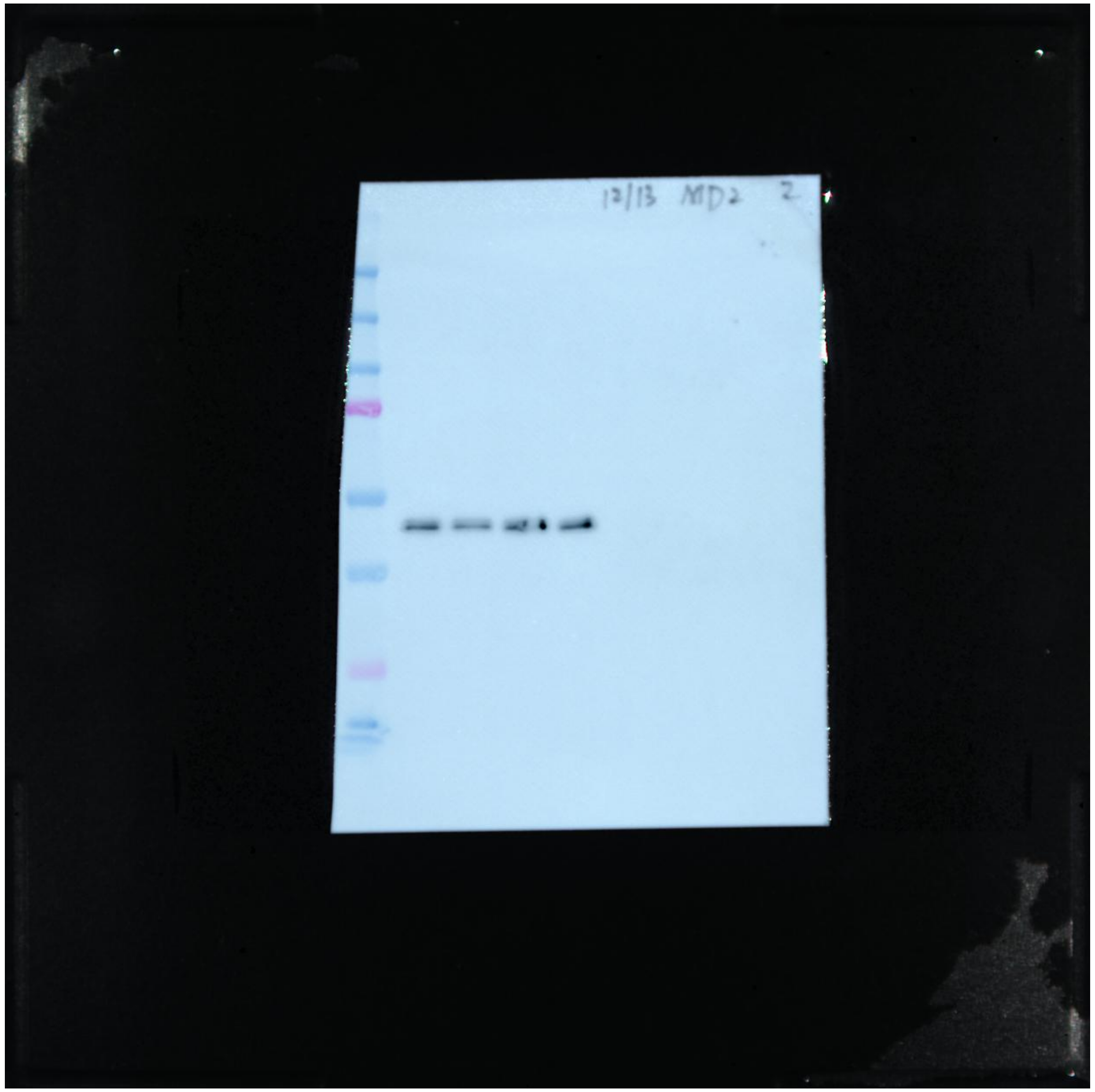

OCT4

Figure 8F

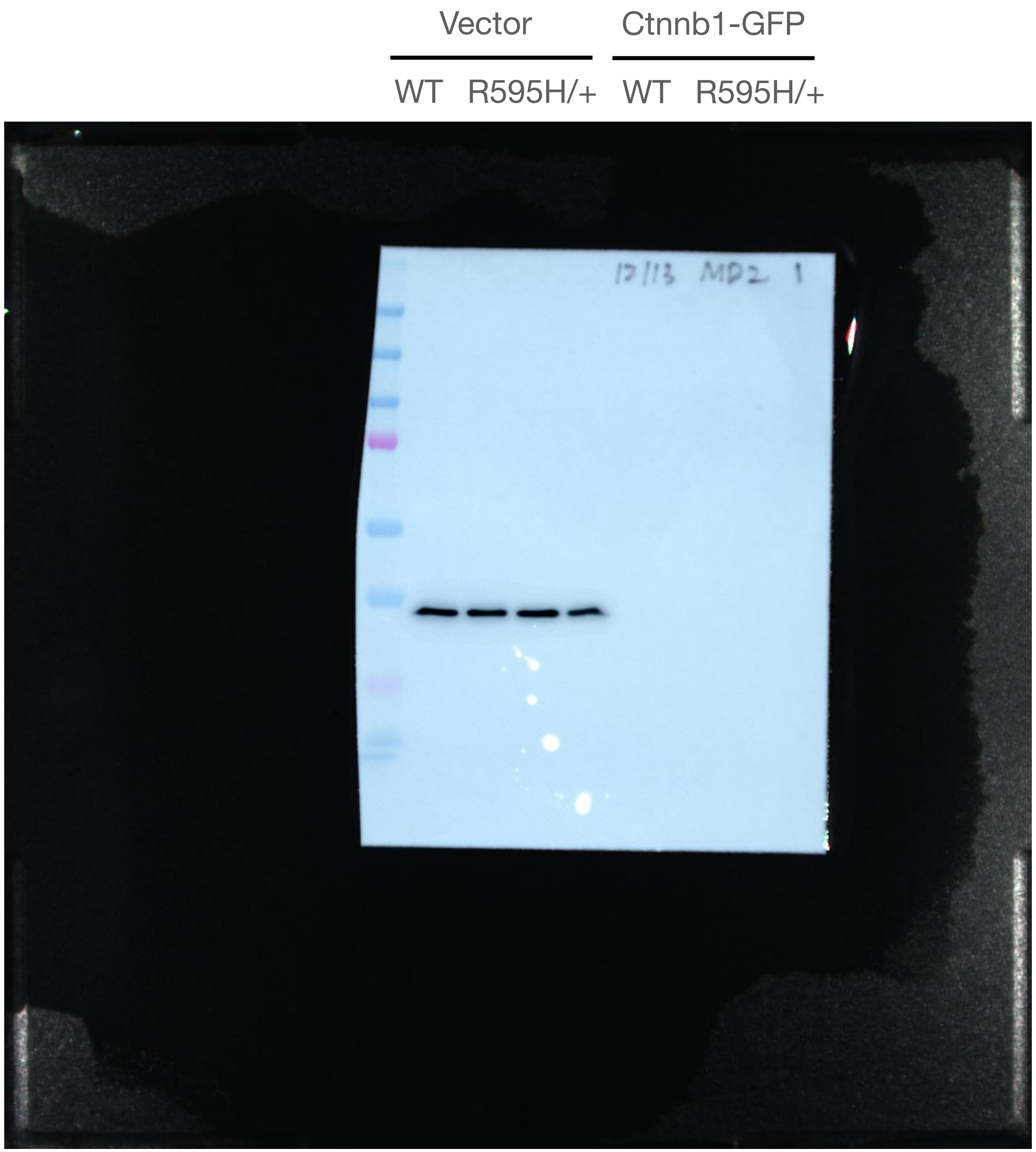

GAPDH

Figure 8G

Vector		Ctnnb1-GFP	
WT	R595H/+	WT	R595H/+

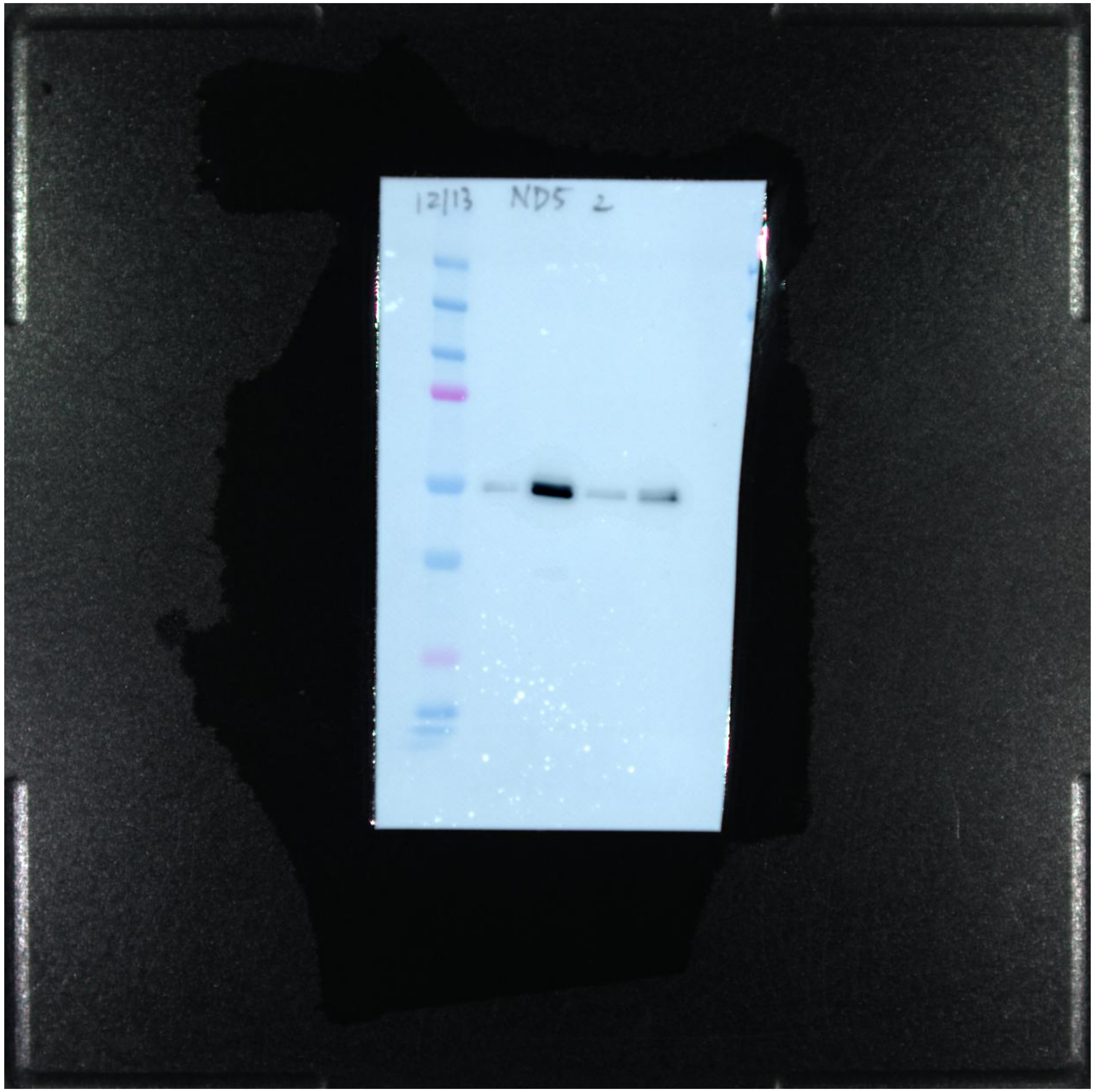

PAX6

Figure 8G

Vector Ctnnb1-GFP

WT R595H/+ WT R595H/+

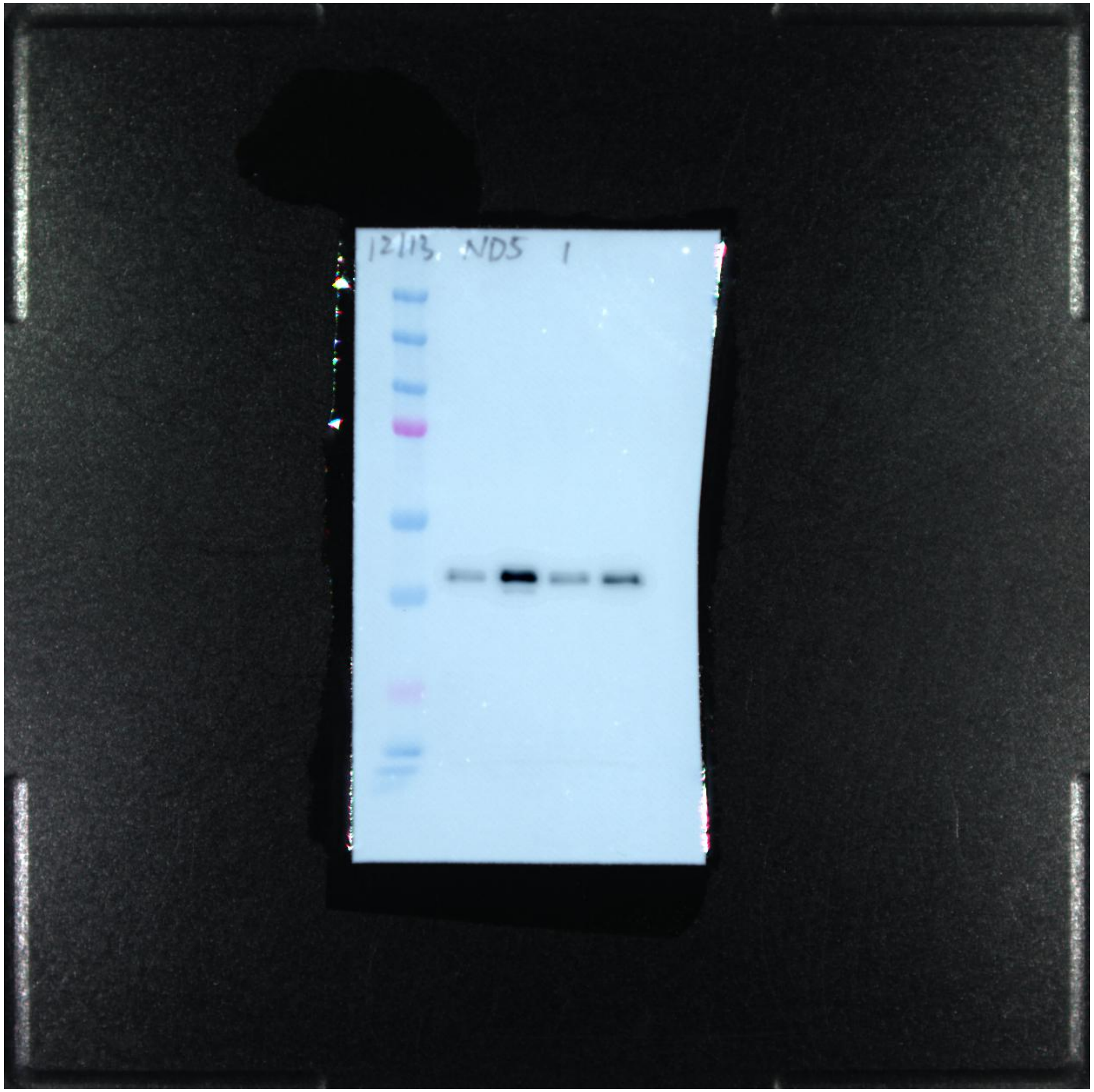

SOX1

Figure 8G

Vector | Ctnnb1-GFP

WT R595H/+ WT R595H/+

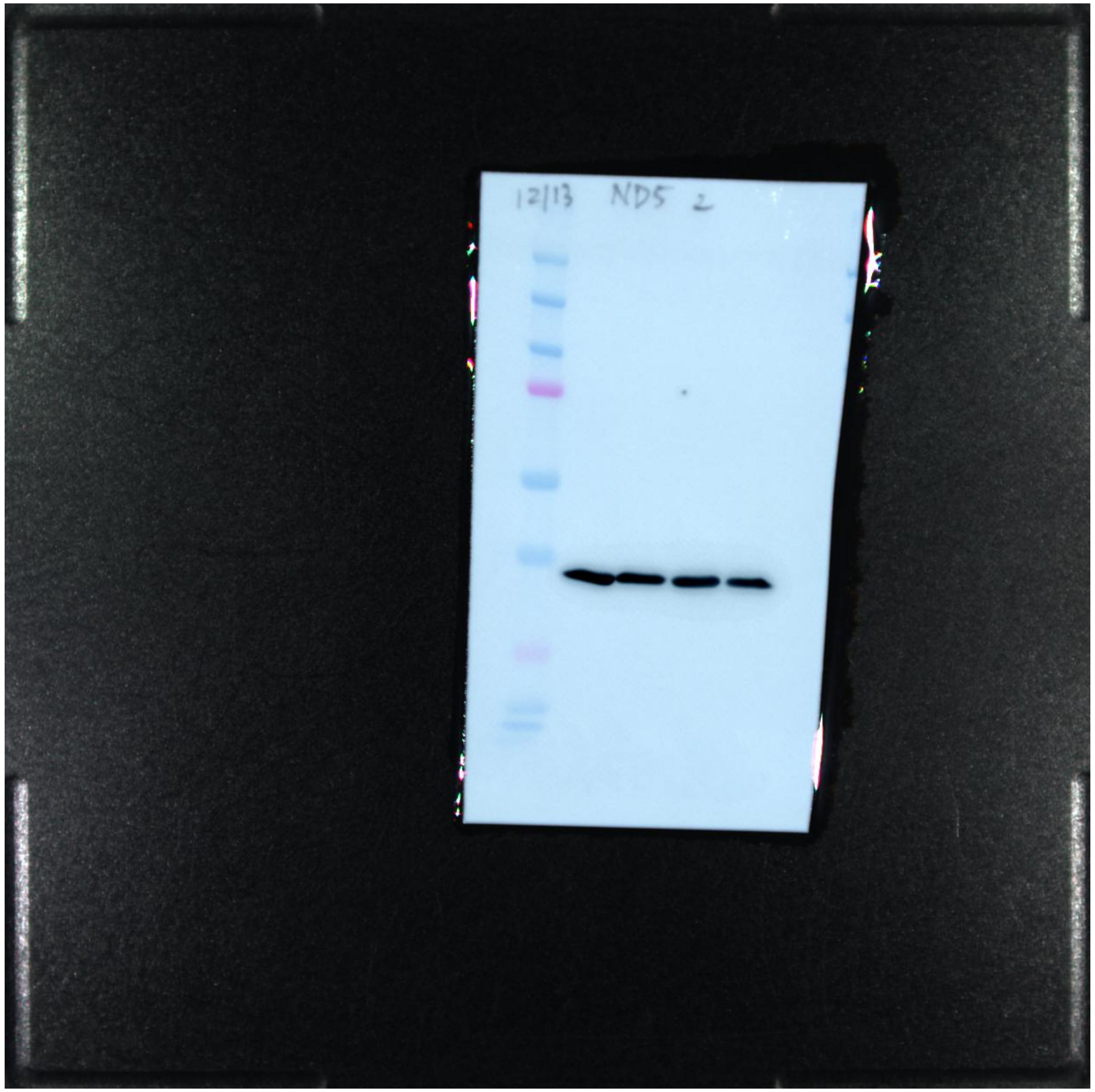

GAPDH

Figure S6A

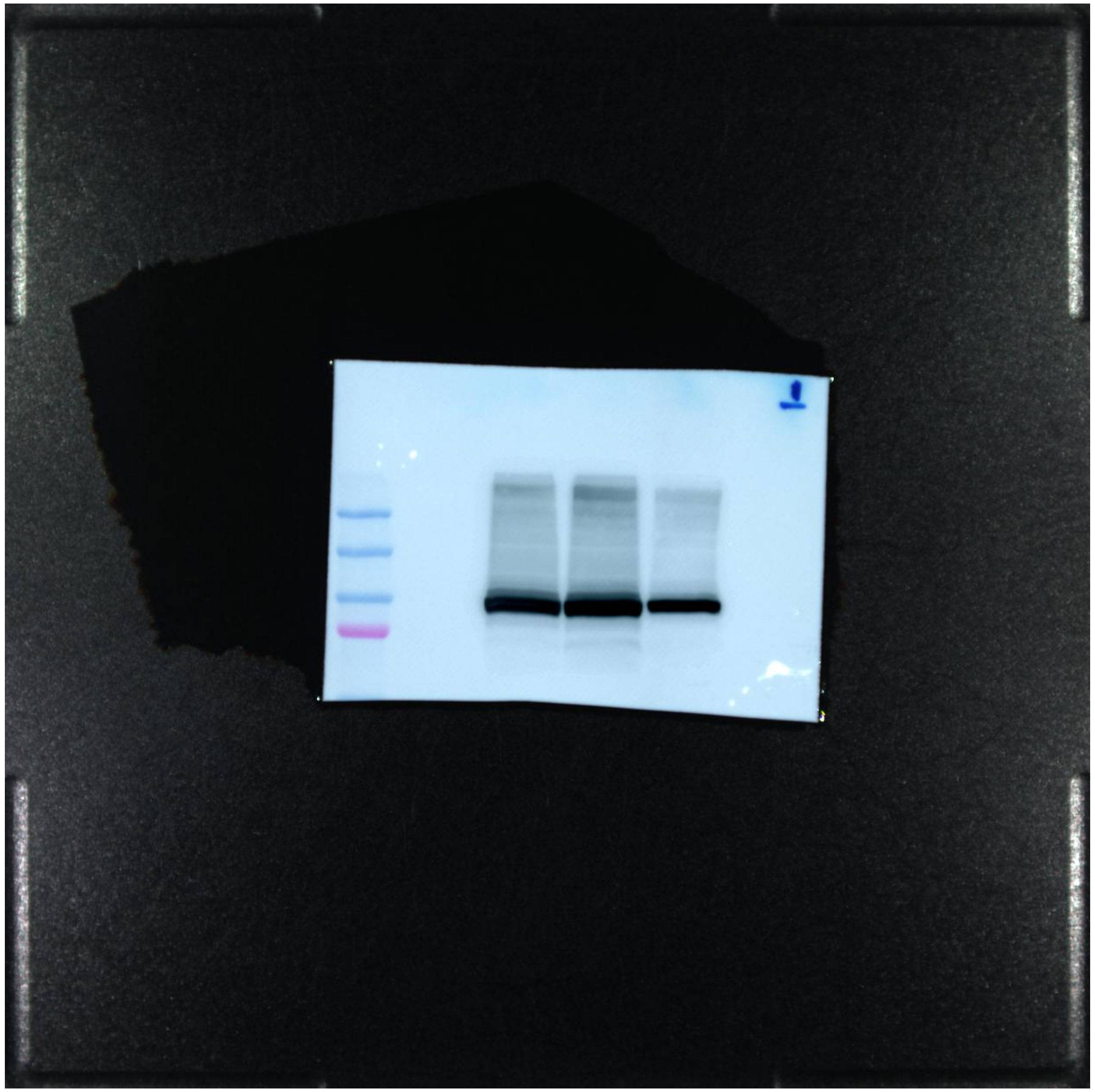

anti-HA

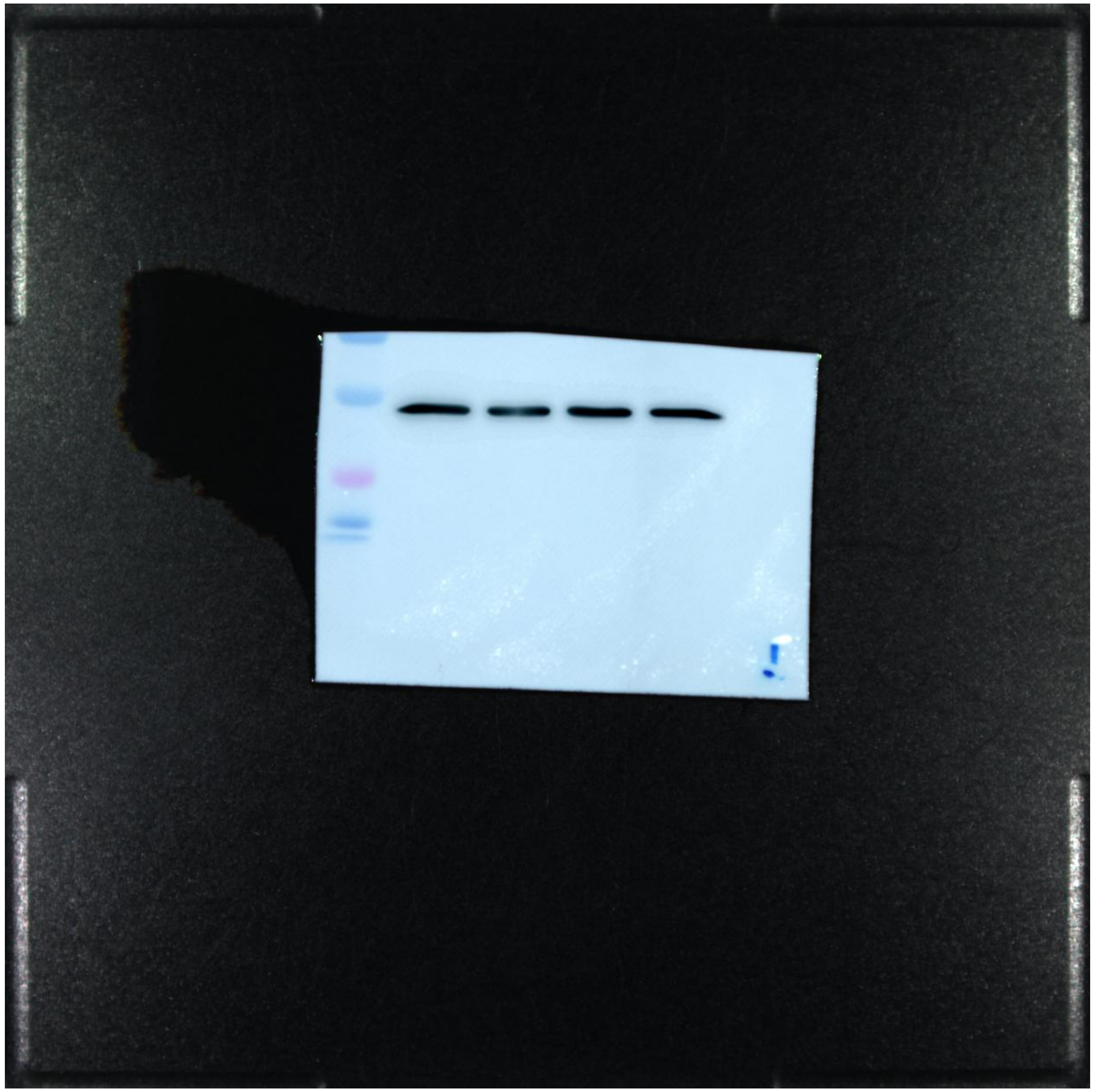

GAPDH

Note: the membrane was cut into halves for probing anti-HA and GAPDH, respectively

Figure S6A

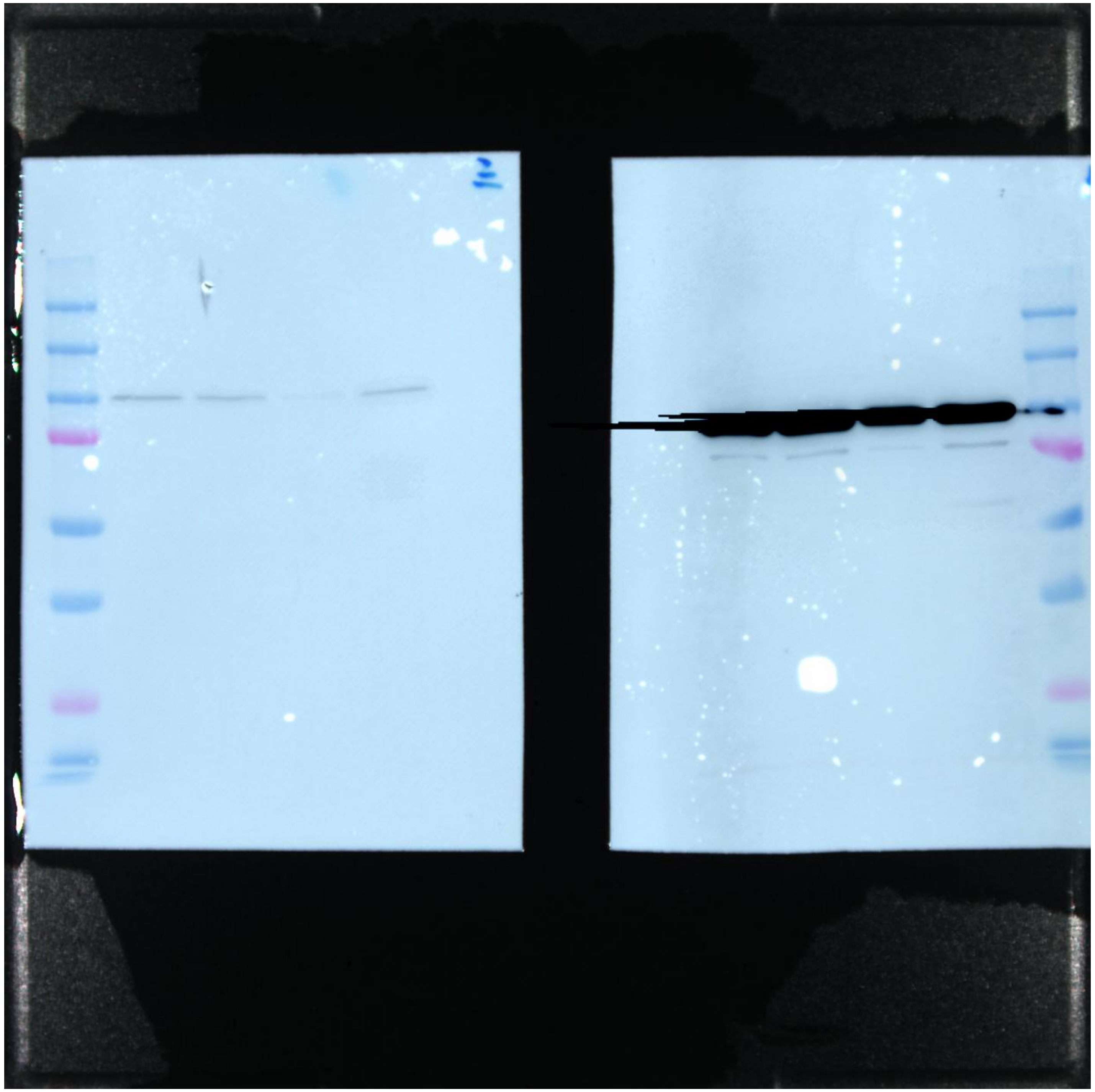

CTNNB1

Figure S6A

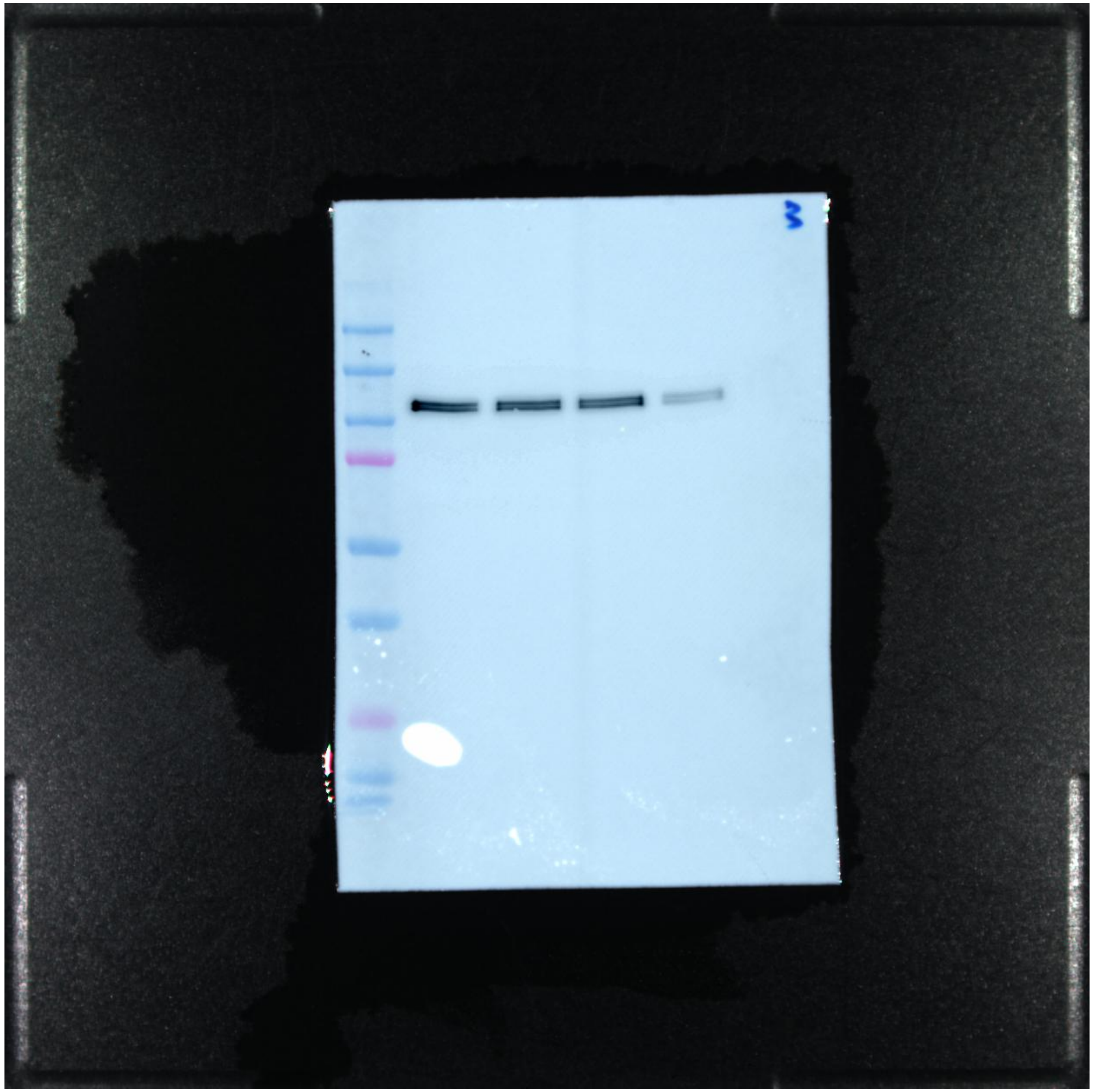

LSD1
